# Structural mechanism of histone H2A.Z exchange by human SRCAP-CFDP1 holoenzyme

**DOI:** 10.1126/sciadv.aei7728

**Published:** 2026-07-31

**Authors:** Giho Park, Carl Wu, Robert K. Louder

**Affiliations:** ^1^ Biochemistry, Cellular and Molecular Biology Graduate Program, Johns Hopkins University School of Medicine, Baltimore, MD 21205, USA.; ^2^ Department of Molecular Biology and Genetics, Johns Hopkins University School of Medicine, Baltimore, MD 21205, USA.; ^3^ Department of Biology, Johns Hopkins University, Baltimore, MD 21218, USA.

## Abstract

The conserved yeast SWR1 and human SRCAP chromatin remodeling complexes catalyze exchange of nucleosomal histone H2A for H2A.Z, but the underlying mechanism has remained obscure. Here, we show that histone exchange by SRCAP requires the transient activator CFDP1 and resolve nine cryo–electron microscopy structures of the SRCAP-CFDP1 holoenzyme that define the stepwise exchange mechanism. CFDP1 recognizes the conformation of the fully engaged SRCAP-nucleosome complex through interactions with multiple subunits—including direct contact with the ATPase domain—and induces conformational transitions that drive extensive DNA unwrapping, eviction of the H2A-H2B dimer, and insertion of the H2A.Z-H2B dimer, all without necessarily requiring hydrolysis of bound ATP. Collectively, these findings provide unprecedented insight into the mechanism of activator- and nucleotide-driven histone exchange from nucleosomal H2A to H2A.Z.

## Teaser

Cryo-EM structures reveal the stepwise mechanism of histone H2A.Z exchange by the human SRCAP chromatin remodeling complex.

## INTRODUCTION

Chromatin organization is integral for genome metabolism (*1*, *2*). Nucleosomes, the fundamental unit of chromatin, comprise 146 base pairs (bp) of DNA encircling eight histone proteins—two copies each of canonical histones H2A, H2B, H3, and H4 (*3*, *4*). Adenosine 5′-triphosphate (ATP)–dependent chromatin remodeling enzymes generally mobilize nucleosomes at regulatory DNA elements, creating accessible DNA to facilitate assembly of the DNA processing machineries (*5*, *6*). Structural advances over the past decade have provided insights on the molecular basis of nucleosome mobilization by SWI/SNF (SWItch/Sucrose Non-Fermentable), ISWI (Imitation SWItch), CHD (Chromodomain Helicase-like DNA-binding), and INO80 (INOsitol requiring protein 80) remodeler families, illuminating a potentially shared mechanism of ATP hydrolysis–driven DNA translocation to reposition nucleosomes without compositional change of the final product (*5*–*7*). However, structural insights on remodeler-driven alterations of histone composition within the octameric nucleosome core, as found biochemically for the ATP-dependent yeast SWR1 (SWI2/SNF2-related 1) complex (*8*), have been elusive. SWR1 and its conserved orthologs are required for deposition of the universally conserved histone H2A.Z variant, a key epigenetic mark at eukaryotic promoters, enhancers, and other genomic sites where H2A.Z serves important functions for transcription, DNA repair, and DNA replication (*9*–*12*).

In humans, the SRCAP (SNF2-Related CREBBP Activator Protein) complex, homolog of the yeast SWR1 complex, has been implicated in ATP-dependent exchange of nucleosomal H2A for H2A.Z (*8*, *13*, *14*). However, while SRCAP is required for H2A.Z deposition at promoters and enhancers in vivo (*14*–*16*), highly purified human SRCAP complex displays no detectable histone exchange activity in vitro [this study and (*17*)]. Thus, unlike constitutively active yeast SWR1, the requirements for enzymatic activation of SRCAP have been elusive. Furthermore, structural insights from other chromatin remodelers offer limited guidance, as H2A.Z exchange entails the eviction of internal histones from the canonical octamer and reintegration of variant histone without net DNA translocation—a mechanistically distinct process.

Here, we show that CFDP1 (CranioFacial Development Protein 1) is an essential activator for SRCAP-mediated histone exchange. We further resolve nine major structural intermediates of the reaction by cryo–electron microscopy (cryo-EM), including a pre-CFDP1, fully engaged state of SRCAP-nucleosome complex that marks the entry point to holoenzyme activation. From this state, CFDP1 binds and triggers an orchestrated series of conformational changes over the entire 15-component complex, leading to extensive DNA unwrapping, coordinated eviction of the canonical histone H2A-H2B dimer, and insertion of the H2A.Z-H2B variant. These findings define the molecular pathway of histone H2A.Z exchange.

## RESULTS

### CFDP1 is an essential, transient activator of SRCAP for histone H2A.Z exchange

**Fig. 1.**
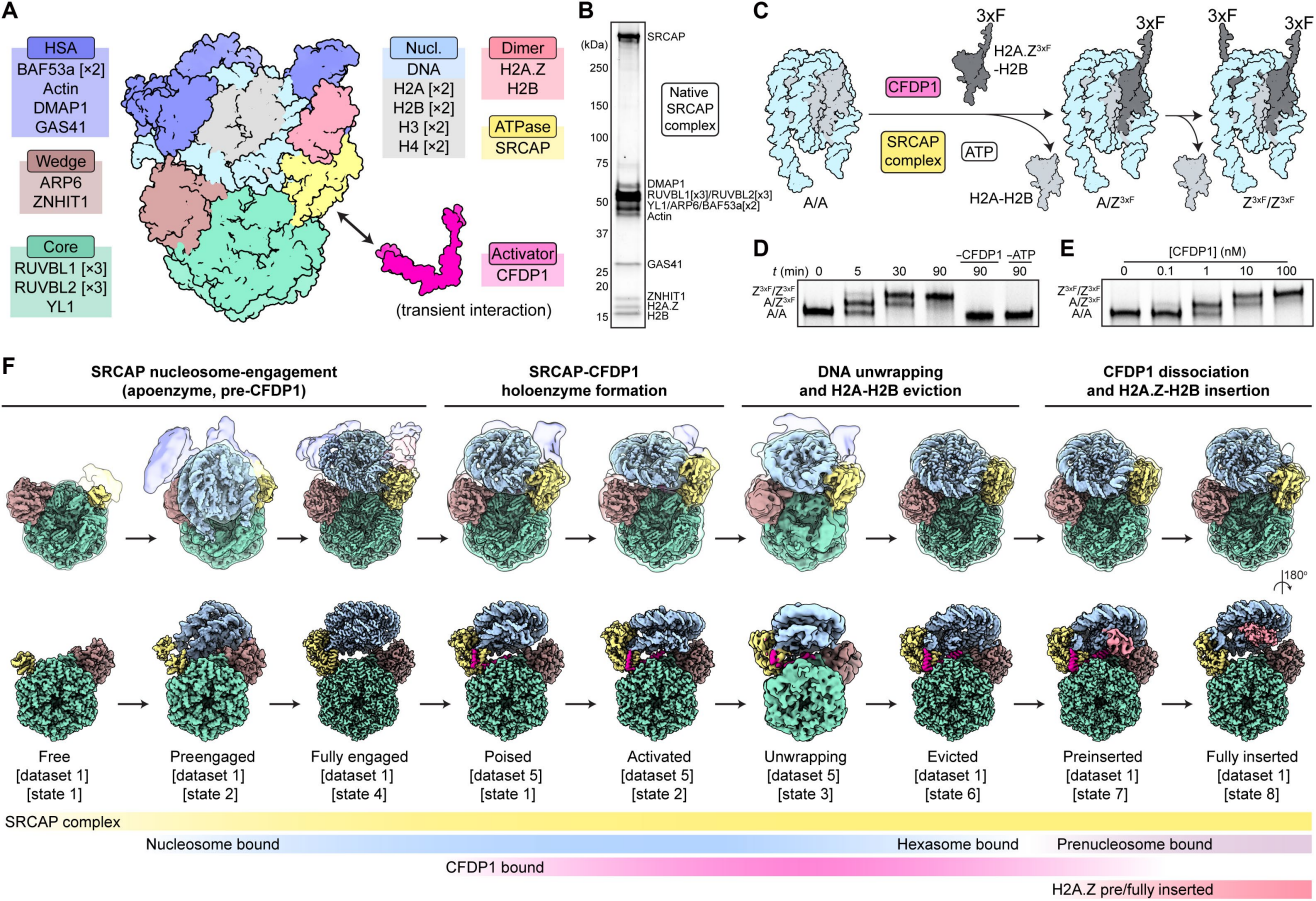
Cryo-EM visualization of the SRCAP-CFDP1 histone H2A.Z exchange reaction. (**A**) Cartoon schematic of the SRCAP-CFDP1-nucleosome ternary complex organized into modules. (**B**) SDS-PAGE of endogenously purified native SRCAP complex. (**C**) Cartoon schematic of histone exchange reaction. Components required for the reaction (A/A nucleosome, SRCAP, CFDP1, H2A.Z^3xF^-H2B, and ATP) and generated products (A/Z^3xF^ nucleosome and Z^3xF^/Z^3xF^ nucleosome) are shown. (**D**) Histone exchange assay results at different time points (0, 5, 30, and 90 min) with all components (lane 1 to 4), without CFDP1 (lane 5, 90 min), and without ATP (lane 6, 90 min). Incorporation of H2A.Z^3xF^-H2B into the nucleosome slows the mobility in native PAGE and separates exchanged from unexchanged nucleosomes. (**E**) Histone exchange assay with different concentrations of CFDP1 (0, 0.1, 1, 10, and 100 nM), showing concentration-dependent activation of SRCAP by CFDP1. (**F**) Cryo-EM reconstructions of SRCAP apoenzyme and SRCAP-CFDP1 holoenzyme resolved throughout the process of nucleosome engagement and histone H2A.Z exchange. Low-pass filtered (transparent, top row only) and high-resolution composite (solid) maps are shown for each state. Bars (bottom) represent sample composition and position of H2A.Z.

To investigate the endogenous SRCAP complex, we purified to homogeneity the native 12-subunit, 1.0-MDa assembly from a gene-edited K562 cell line (Fig. 1, A and B; fig. S1; and Materials and Methods). While yeast SWR1 can be purified with the full complement of subunits required for the ATP-dependent histone exchange reaction (*8*), purified human SRCAP complex shows no histone exchange activity in vitro [this study and (*17*)]. Of the four yeast-specific SWR1 subunits absent from human SRCAP—yeast Swc3, Swc5, Swc7, and Bdf1—only Swc5 is essential for the histone exchange activity of SWR1 (*18*, *19*) and has been specifically shown to stimulate Swr1 adenosine triphosphatase (ATPase) activity and to chaperone the evicted H2A-H2B dimer (*20*, *21*). The human homolog of Swc5 is CFDP1 (also called BCNT/Bucentaur) (*22*–*25*).

To examine the role of CFDP1 in histone exchange, we incubated purified full-length human CFDP1 with native SRCAP complex, canonical nucleosomes containing two copies of H2A-H2B dimer (A/A nucleosomes), 3xFLAG-tagged H2A.Z-H2B dimers, and ATP (Fig. 1, C and D, and fig. S2, A to H) (*8*, *26*, *27*). Subsequent to the reaction, excess H2A.Z-H2B dimer is necessarily adsorbed by addition of lambda carrier DNA and samples are analyzed by native polyacrylamide gel electrophoresis (PAGE) (fig. S3, A to C) (*27*). The stepwise exchange of the canonical dimer for H2A.Z-H2B retards the electrophoretic mobility of the nucleosome product due to the presence of one or two 3xFLAG tags on H2A.Z-H2B, allowing the facile identification of unreacted A/A, heterotypic A/Z, and homotypic Z/Z nucleosomes (Fig. 1, C and D) (*26*).

The addition of CFDP1 activates SRCAP for robust histone exchange in a concentration- and ATP hydrolysis–dependent manner (Fig. 1, D and E); no activity is observed with ATPγS, adenosine 5′-diphosphate (ADP), or without nucleotide (fig. S3D). Despite this clear requirement for CFDP1 in the H2A.Z exchange reaction, the interaction between CFDP1 and the SRCAP complex appears to be highly labile. CFDP1 is undetectable in our native SRCAP purification from human cell extracts (Fig. 1B), and SRCAP neither copurifies with overexpressed CFDP1 from human cells (fig. S3E), nor comigrates stoichiometrically upon glycerol gradient centrifugation (fig. S3F). We confirmed the lability of association by three-color electrophoretic mobility shift assay (EMSA) with labeled nucleosomes, SRCAP, and CFDP1 that fails to detect a stable ternary complex (fig. S3G).

### Cryo-EM visualization of the SRCAP histone exchange reaction

To capture the mechanism of SRCAP-CFDP1 “holoenzyme” formation and activation, we collected and analyzed 12 experimentally distinct cryo-EM datasets from systematically varied mixtures of purified SRCAP, CFDP1, nucleosomes, and ATP (or nonhydrolyzable analogs), yielding a large and comprehensive series of structural intermediates (Fig. 1F, figs. S4 to S23, tables S1 to S8, and Materials and Methods). To preserve reaction intermediates, we eschewed further purification of reaction mixtures and conducted extensive three dimensional in silico classification of the heterogeneous cryo-EM data to resolve distinct structural states adopted throughout the process of nucleosome engagement and histone exchange (Fig. 1F, figs. S4 to S23, tables S1 to S8, and Materials and Methods). Together, the structural information from 12 distinct cryo-EM datasets, together with supporting biochemical results, converges to yield a unified mechanistic model of the stepwise SRCAP-CFDP1–mediated histone H2A.Z exchange reaction and elucidates the role of ATP binding and hydrolysis in regulating the process.

### Apo-SRCAP engages the nucleosome and initiates ARP6-ZNHIT1–driven DNA unwrapping

**Fig. 2.**
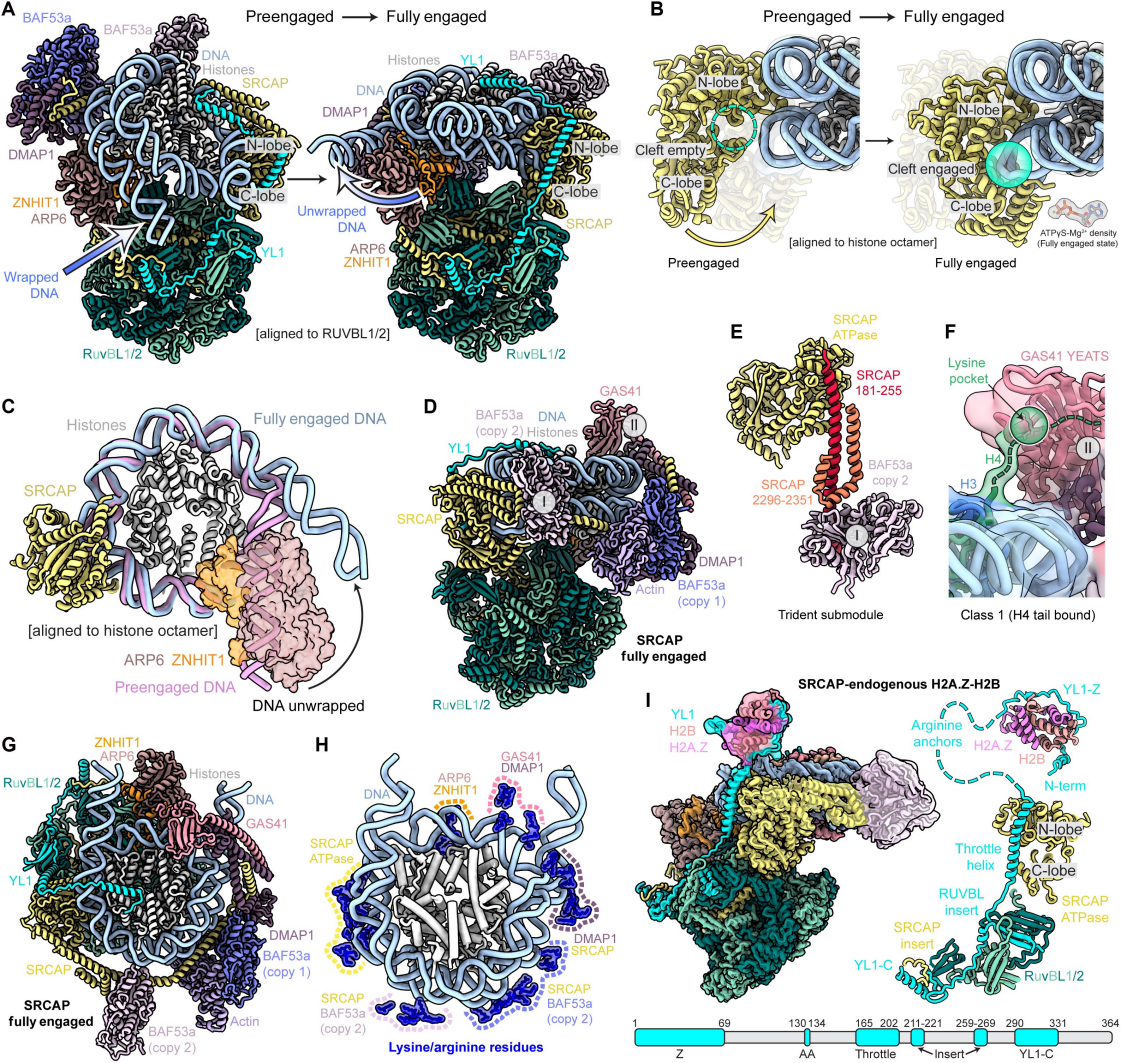
SRCAP transition from pre- to fully engaged disrupts histone-DNA contacts. (**A**) Cryo-EM structures of the pre- and fully engaged SRCAP-nucleosome complexes shown aligned to the RUVBL1/2 module. The wrapped DNA in the preengaged state and unwrapped DNA in the fully engaged state are highlighted with blue arrows. (**B**) Conformational shift of the SRCAP ATPase in the transition from preengaged to fully engaged. The empty and DNA bound ATPase cleft are labeled with a dashed and solid green sphere, respectively. The cryo-EM density of ATPγS-Mg^2+^ in the N-lobe nucleotide pocket is shown (bottom right). Atoms are colored by elements (red: oxygen, blue: nitrogen, orange: phosphate, green: magnesium). (**C**) During the transition from preengaged (pink) to fully engaged (blue), the ARP6-ZNHIT1 subunits wedge between the histone core and unwrap ∼20 bp of DNA. (**D**) Complete cryo-EM structure of the fully engaged SRCAP-nucleosome complex. Roman numerals are used to label different regions of the complex. (**E**) Isolated view (region I) of the trident submodule with SRCAP ATPase, N-terminal HSA helix, and C-terminal region colored yellow, red, and orange, respectively. (**F**) Cryo-EM density (region II) of the H4 tail bound class is shown with the lysine pocket of GAS41 YEATS domain highlighted green. The H4 tail is shown as a dashed green line. (**G**) Structure of the fully engaged SRCAP-nucleosome complex [as in (D)] viewed top-down. (**H**) Lysine and arginine atoms of SRCAP that likely contact the nucleosome DNA are shown. The submodules and the corresponding subunits are indicated with dashed lines. (**I**) Putative docking of YL1-H2A.Z-H2B crystal structure (Protein Data Bank: 5FUG) into cryo-EM density adjacent to the SRCAP ATPase and HSA helix (left). Isolated view of YL1 subunit organization (right). Domain map of YL1 is shown below. The chaperone domain is labeled “Z’ and arginine anchors as “AA.”

We first describe structural states of the SRCAP apoenzyme (before CFDP1 binding), which reveal the mechanism of nucleosome engagement, partial DNA unwrapping, and the adoption of a conformation primed for CFDP1 recruitment and holoenzyme assembly. The Snf2-type ATPase of SRCAP is split into N-terminal and C-terminal lobes (Fig. 2, A and B), between which a DNA binding cleft is formed. We identified a distinct state of nucleosome bound SRCAP in which the ATPase is contacting the catalytically active SHL2 position but without the DNA fully bound within the cleft (Fig. 2, A and B, and movie S1). In this state, the ATPase is splayed wide open and only the N-lobe interacts with SHL2 and SHL6 (Fig. 2, A and B). In addition, a positively charged loop of ZNHIT1 contacts the H2A-H2B dimer near the acidic patch (fig. S24A). We term this state “preengaged” (“dataset 1 state 2” and figs. S6 and S13C), as the ATPase has targeted SHL2 but is not yet fully engaged. Notably, in this state, the nucleosome is spatially distant from the SRCAP core module and the nucleosomal DNA remains unperturbed (Fig. 2A). Furthermore, the N terminus of the DMAP1 (DNA Methyltransferase 1-Associated Protein 1) subunit is bound by ARP6 through a hydrophobic pocket that accommodates L86 of DMAP1 (fig. S24, C and D). This interaction is disrupted upon nucleosome engagement as histone H3 competes for the same pocket (fig. S24D and Supplementary Text).

In the subsequent, fully engaged state (“dataset 1 state 5” and figs. S6 and S13A), the SRCAP ATPase cleft is stably bound to SHL2, the nucleosome is positioned closer to the RUVBL1/2 core, and ∼20 bp of DNA are unwrapped near SHL6 to SHL7 (Fig. 2, A to C; fig. S24B; and movie S1). Presumably, the engagement of the ATPase cleft of SHL2 DNA induces conformational changes of the connected HSA module, spatially shifting the nucleosome closer to the core module to attain the fully engaged position (Fig. 2, A to C, and fig. S24D). During this transition, the nucleosome is physically wedged by the ZNHIT1-ARP6 subunits, detaching ∼20 bp of DNA from the histone octamer (Fig. 2C and movie S1).

Given that the two lobes of the SRCAP ATPase in the preengaged state exhibit a more “open” configuration compared to the fully engaged state, we speculated that nucleotide binding facilitates engagement. A distinct cryo-EM dataset of SRCAP-nucleosome complex assembled in the absence of any nucleotide contained only the preengaged state (“dataset 2” and fig. S8A), confirming that the transition from the preengaged to the fully engaged state is contingent on the presence of nucleotide. Accordingly, we propose that SRCAP transitions from the preengaged state to the fully engaged state as a mechanism to disrupt histone-DNA contacts independent of ATP hydrolysis.

### HSA module of SRCAP encircles the nucleosome through extensive multivalent interactions

The predominant fully engaged state (dataset 1 state 4, figs. S6 and S13A, and movie S1) resembles previously reported SWR1-nucleosome structures, with the ATPase stably engaged on SHL2 (Fig. 2D). Overall, the human complex is organized similarly as previously reported yeast SWR1-nucleosome structures (*28*, *29*), featuring the two key nucleosome binding modules, SRCAP/YL1 and ZNHIT1/ARP6, positioned on opposite sides of the RUVBL1/2 heterohexamer, into which the SRCAP insert domain is incorporated (Fig. 2D and fig. S25, A and B). Further classification of the cryo-EM data reveals the partially engaged state (dataset 1 state 3 and figs. S6 and S13B), which we further discuss in Supplementary Text.

Beyond the SRCAP core module, the N-terminal 255 amino acids of SRCAP subunit includes the HSA (helicase/SANT-associated) domain, which serves as a structural scaffold for the assembly of a 250-kDa module composed of GAS41 (Glioma-Amplified Sequence 41), DMAP1, actin, and two copies of BAF53a (Fig. 2D and movie S1). Strikingly, in the fully engaged state, the SRCAP HSA extended α helix arcs ∼80° to envelop the nucleosome (Fig. 2D). This HSA module is divided into three structural submodules. The first trident-shaped submodule includes the C-terminal end of SRCAP HSA helix that extends outward from the ATPase and interacts with BAF53a (copy 2) and another conserved C-terminal segment of the SRCAP subunit (Fig. 2E and fig. S25C). Notably, in a previous cryo-EM study (*17*), this region was modeled as actin rather than BAF53a (fig. S25C). This difference potentially stems from the use of ectopically overexpressed SRCAP (*17*) rather than the endogenous complex (this study). The second A submodule consists of the extended HSA of SRCAP, actin, BAF53a (copy 1), and DMAP1 (Fig. 2D and fig. S25D). The third GAS41 submodule forms a tetrameric coiled-coil structure with DMAP1, GAS41, and the N terminus of the SRCAP subunit (Fig. 2, D and F, and fig. S26A).

Intriguingly, within the nucleosome-bound complex, GAS41 is positioned near the histone H4 N-terminal region (Fig. 2, D and F and Supplementary Text). Further classification of the cryo-EM data revealed a map with connecting density between the YEATS domain of GAS41 and the H4 histone core, indicative of the unmodified H4 tail (Fig. 2F and fig. S26B). While we can trace the H4 N-terminal tail from the nucleosome core to the lysine pocket and speculate that the unmodified H4K16 is most likely bound to the YEATS domain (Fig. 2F and fig. S26, C and D), we cannot rule out interactions with more N-terminal lysines such as H4K5/8/12.

In this state, the SRCAP complex establishes extensive electrostatic contacts with the DNA through multiple subunits and modules (Fig. 2, G and H). The SRCAP ATPase binds to SHL2 and SHL6. ZNHIT1 binds to the dyad at SHL0. The trident submodule contacts SHL3 and SHL−4. The A submodule contacts SHL4 to SHL5. SRCAP and DMAP1 contact SHL5 and SHL6. Last, the GAS41 and DMAP1 contact SHL−2 to SHL−1.

In addition, we observe cryo-EM density for the endogenously copurified H2A.Z-H2B dimer (Fig. 2I and fig. S2, A and B). The N-terminal region of YL1 contains the H2A.Z-H2B chaperone domain for which the crystal structure has been previously determined (*30*, *31*). We could putatively dock this YL1-H2A.Z-H2B structure into low-resolution cryo-EM density adjacent to the SRCAP ATPase (Fig. 2I). Notably, the catalytic SRCAP subunit also contains a second H2A.Z-H2B chaperone domain adjacent to the N-lobe of the ATPase that may function cooperatively with the YL1 chaperone domain (fig. S26, E and F) (*32*). Our docking reveals the close proximity of H2A.Z-H2B with the SRCAP ATPase and SRCAP HSA helix, suggesting a coupling between ATP hydrolysis and dimer release for histone exchange (Fig. 2I) (*33*). The fully engaged SRCAP apoenzyme now adopts a structural conformation preconfigured for CFDP1 incorporation and holoenzyme formation as discussed below.

### Structural mechanism of SRCAP-CFDP1 holoenzyme formation

**Fig. 3.**
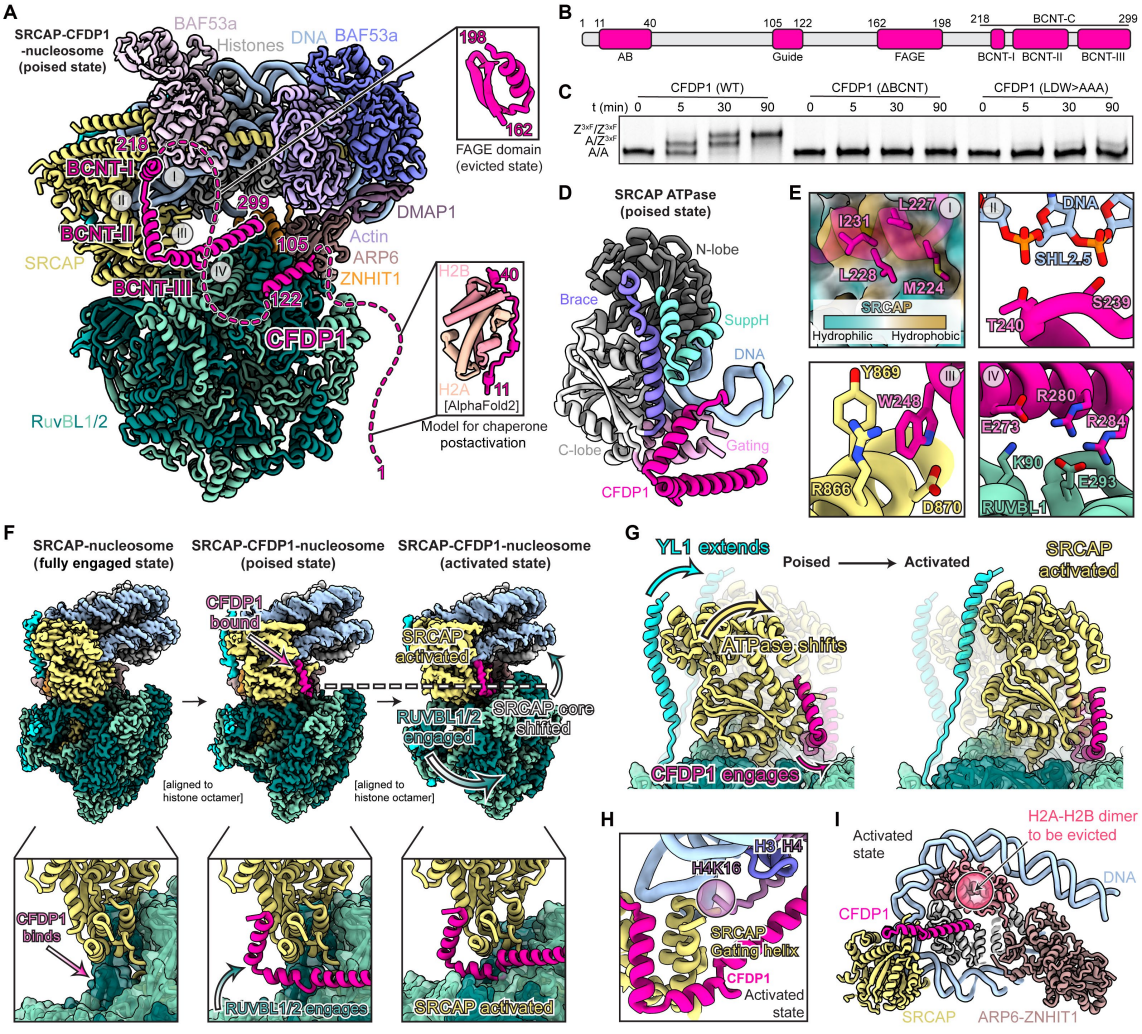
Structural basis for SRCAP-CFDP1 holoenzyme formation and activation. (**A**) Cryo-EM structure of nucleosome-bound SRCAP-CFDP1 holoenzyme in the poised state, with unobserved flexible linkers between structured CFDP1 domains shown as dashed lines. Roman numerals labeling different regions of the complex match panels in Fig. 3E. Top inset: Structure of the FAGE domain in the evicted state. Bottom inset: AlphaFold2 model for the N-terminal histone H2A-H2B chaperone domain of CFDP1. The histone octamer is intact in the poised state and the bottom inset model represents chaperone function post-eviction. (**B**) Domain diagram of human CFDP1. (**C**) Histone exchange assay at different time points (0, 5, 30, and 90 min) with WT, ∆BCNT, and LDW alanine substitution mutant (L^247^D^248^W^249^→A^247^A^248^A^249^) for CFDP1. (**D**) Isolated view of the SRCAP ATPase colored by domain (N-lobe: dark gray; C-lobe: light gray; SuppH: Cyan; Brace: Purple; Gating: light pink). (**E**) Close-up views of CFDP1 interactions with SRCAP HSA module (I), DNA (II), SRCAP gating helices (III), and RUVBL1 (IV). (**F**) Cryo-EM composite maps of SRCAP-nucleosome in the engaged (pre-CFDP1), poised, and activated states (maps aligned to histone octamer), showing stepwise activation through CFDP1 binding and subsequent engagement of RUVBL1/2. The white dashed line serves as a reference for visualization of the shift of the RUVBL1/2 core between the poised and activated states. Zoom-in views of the SRCAP^ATPase^, CFDP1^BCNT-II^, and interacting RUVBL1/2 core are shown at bottom. (**G**) Conformational transition of the SRCAP^ATPase^ and YL1 throttle helix in the poised and activated states (aligned to RUVBL1/2 core). (**H**) Zoom-in view showing histone H4 tail interaction with CFDP1 and SRCAP gating helices near SHL2. (**I**) Isolated view of the nucleosome showing the H2A-H2B dimer to be evicted (labeled with a transparent red sphere).

Next, we characterize structural states of the SRCAP-CFDP1 holoenzyme that reveal how CFDP1 binds and primes SRCAP for activation. Despite biochemical lability, we were able to capture the SRCAP-CFDP1 holoenzyme by assembling reactions at micromolar protein concentrations with mild glutaraldehyde crosslinking, and vitrification preserved these otherwise unstable interactions for cryo-EM analysis (see Materials and Methods). We first describe the poised state of the SRCAP-CFDP1-nucleosome ternary complex (“dataset 5 state 1” and figs. S9A and S15D), which structurally resembles the fully engaged apo-SRCAP state but now includes several domains of CFDP1, which form multiple contacts with key components of the SRCAP complex (Fig. 3A).

### CFDP1 BCNT-C helices recognize fully engaged SRCAP through multiple distinct interactions to form the poised state

The most well-resolved domain of CFDP1 in the poised state cryo-EM structure (dataset 5 state 1) corresponds to the highly conserved BCNT-C domain, which can be structurally divided into three subdomains we term BCNT-I, BCNT-II, and BCNT-III, that interact with the SRCAP, BAF53a (copy 2), and RUVBL1/2 subunits (Fig. 3, A and B; fig. S27, A to G; and movie S2). This BCNT-C domain is required for histone exchange (Fig. 3C). The BCNT-III domain comprises a 26-residue α helix on the C-terminal end of CFDP1 that binds along the β-stalk of the RUVBL1 subunit proximal to the SRCAP^ATPase^ and the OB-fold of the adjacent RUVBL2 subunits (Fig. 3, A, B, and E, IV).

BCNT-III is connected via a short loop to the BCNT-II domain, which comprises two short α helices that envelope the gating helices of the SRCAP^ATPase^ that insert into the DNA minor groove near SHL2.5 on nucleosome engagement (Fig. 3, A and D, and movie S2). The first α helix of the BCNT-II domain contains the highly conserved LDW motif (*20*) that is required for histone exchange (Fig. 3C), with the tryptophan of the motif (W248 in human CFDP1) appearing to be especially important in forming the interaction with the SRCAP gating helices (Fig. 3E, III). This BCNT-II helix additionally contacts the brace element (*34*) of the SRCAP^ATPase^ and is preceded by a loop that interacts with the SRCAP^ATPase^ SuppH element (*35*) and nucleosomal DNA at SHL2.5 (Fig. 3, A, D, and E, II).

Last, the BCNT-I domain immediately preceding the BCNT-II domain is composed of a short α helix that interacts with the SRCAP trident submodule [Figs. 2E and 3, A and E (I); Supplementary Text; and movie S2] (*29*). Notably, the short linker connecting the BCNT-I and BCNT-II helices is only compatible with simultaneous binding to their respective sites on the HSA module and SRCAP^ATPase^ in the fully engaged SRCAP conformation, as these sites are too far apart to allow CFDP1 binding to the preengaged state of SRCAP (fig. S28, A and B).

Consistent with the biochemically labile SRCAP-CFDP1 interaction, we do not observe CFDP1 binding to SRCAP in any structural states preceding the fully engaged SRCAP-nucleosome state (figs. S4 and S5). Together, these results suggest that the conformation of this state presents a higher-affinity interface for CFDP1^BCNT^; with BCNT-III interacting with the RUVBL1/2 subunits; with BCNT-II recognizing the specific conformation of the gating, brace, and SuppH elements of the SRCAP^ATPase^ in addition to the bound nucleosomal DNA; and with BCNT-I binding sensitive to the positioning of the SRCAP HSA module relative to the SRCAP^ATPase^.

Beyond the core BCNT domain, the N-terminal two-thirds of CFDP1 contain three additional, evolutionarily conserved domains: the AB chaperone domain (residues 11 to 40), the guide helix (residues 105 to 122), and the FAGE domain (residues 162 to 198) (Fig. 3, A and B, and fig. S27, A to E). The structural and biochemical details of these conserved CFDP1 domains are described in the Supplementary Text.

### CFDP1 induces an activated SRCAP conformation that further promotes nucleosome destabilization

In the poised state, the conformation of the catalytic SRCAP^ATPase^ bound to CFDP1 remains virtually unchanged relative to its fully engaged state lacking CFDP1 (Fig. 3F). By contrast, transition to the activated state (‘”dataset 5 state 2”) involves a shift in the RUVBL1/2 heterohexamer that sandwiches the second BCNT-II helix of CFDP1 between RUVBL1 and the SRCAP^ATPase^ (Fig. 3F and movie S2). The throttle helix of the YL1 subunit partially uncoils and extends to accommodate this movement of the SRCAP^ATPase^ relative to the RUVBL1/2 core (Fig. 3G). This SRCAP^ATPase^ shift positions the HSA module and nucleosome closer to the RUVBL1/2 core (Fig. 3F; fig. S28, E and F; and movie S2). Accordingly, the C-terminal BCNT-III helix of CFDP1 forms additional interactions with RUVBL1 and is positioned near the histone octamer core between the H2A-H2B dimer to be evicted and the H3-H4 tetramer (Fig. 3I).

The BCNT domain additionally sequesters the H4 tail, which extends to interact with the SRCAP^ATPase^ gating helices (Fig. 3H). This H4 tail interaction differs from other chromatin remodelers for which the H4 tail interacts with the acidic surface of the RecA2 ATPase domain on the opposite side of the gating helices (fig. S29, A and B) (*36*–*38*). Notably, in the fully engaged state (“dataset 1 state 5”), the GAS41 subunit binds to the H4 tail on the other face of the nucleosome (Fig. 2, D and F, and fig. S29, A, C, and D), suggesting that the two H4 tails may separately regulate SRCAP at the recruitment and activation stages.

In the transition from pre- to fully engaged, the ARP6-ZNHIT1 wedge drives the unwrapping of ∼20 bp of DNA on the side of the nucleosome where histone exchange occurs (Fig. 2, A and C). Upon activation, the nucleosome is drawn closer to the RUVBL1/2 heterohexamer, and this ARP6-ZNHIT1 wedge unwraps DNA by several additional bp to further expose the H2A-H2B dimer near SHL5 (Fig. 3I and movie S2). This transition from poised to activated state requires full-length CFDP1, as a cryo-EM sample with a truncated CFDP1 mutant protein lacking part of the BCNT-III domain (Δ284 to 299) adopts only the poised state (“dataset 8” and figs. S10C and S16D). Intriguingly, ATP hydrolysis is not required because a distinct cryo-EM dataset with ADP substituting for ATP shows a comparable fraction of the activated state (41%, compared to 62% with ATP) [“datasets 5 and 7” and figs. S9A, S10B, S15 (D and E), and S16 (B and C)]. Thus, this poised-to-activated conformational transition, which further destabilizes the nucleosome and primes the H2A-H2B dimer for eviction, is driven by multiple CFDP1 interactions with SRCAP.

### Formation and stabilization of the hexasome intermediate by SRCAP-CFDP1 holoenzyme

**Fig. 4.**
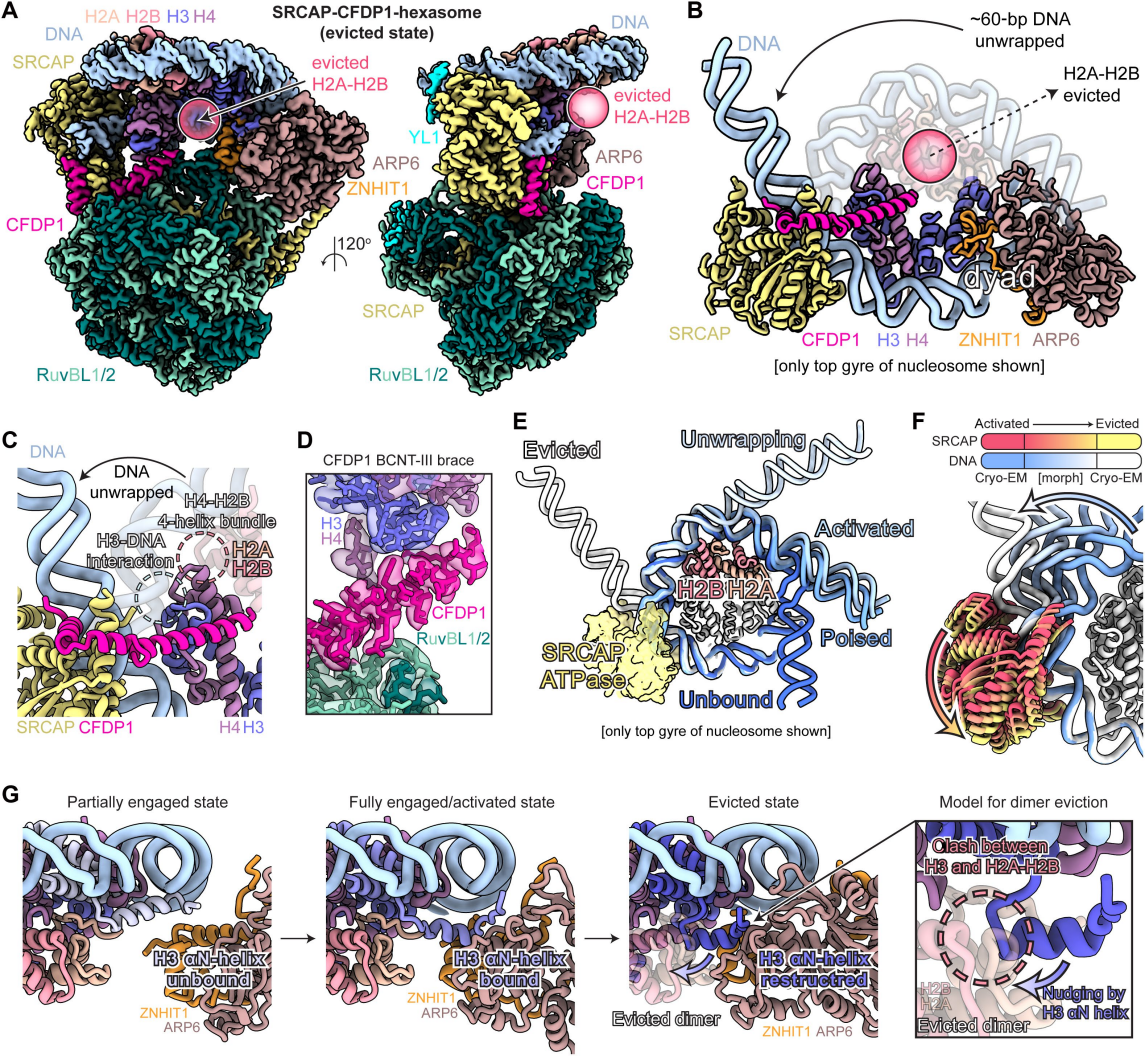
SRCAP-CFDP1 unwraps nearly a full gyre of DNA and restructures H3 αN helix to evict H2A-H2B. (**A**) Cryo-EM composite map of SRCAP-CFDP1-hexasome complex in the evicted state. Two 120° rotated views are shown. The evicted H2A-H2B dimer is labeled with a transparent red sphere. (**B**) Superposition of the de novo unwrapped hexasome (evicted state) and nucleosome (transparent, activated state). The two states were aligned to the histone octamer and only the top gyre of the hexasome/nucleosome is shown. Approximately 60 bp of DNA are unwrapped and the H2A-H2B dimer (transparent red sphere) is evicted. (**C**) Zoom-in view of CFDP1 BCNT domain shows interaction with the H3-H4 tetramer in the hexasome, which has lost stabilizing DNA and H2A-H2B interactions (indicated with dashed circles). (**D**) Cryo-EM density and model of CFDP1 BCNT-III brace which is sandwiched between the H3-H4 tetramer and the RUVBL1/2 core. (**E**) Superposition of the nucleosomal DNA observed in the unbound (free nucleosome), poised, activated, unwrapping, and evicted state cryo-EM structures (all structures aligned to histone octamer). The SRCAP^ATPase^ positioned at SHL2 and the evicted H2A-H2B dimer are highlighted, and only the top gyre of the hexasome/nucleosome is shown. (**F**) Structural transition from the activated to evicted state, showing SRCAP^ATPase^ conformational change and DNA unwrapping. The cryo-EM structures and interpolated morph models are colored according to the key above. (**G**) Zoom-in view of H3 αN helix in the partially engaged (unbound), fully engaged/activated (bound), and evicted (restructured) states. Inset shows clash between restructured H3 αN helix in the evicted state and an overlay of H2A-H2B dimer position before eviction.

Subsequent to CFDP1 activation, we observe an evicted state, corresponding to a de novo generated hexasome as a bona fide reaction intermediate of histone exchange (“dataset 1 state 6,” figs. S7 and S14B, and Materials and Methods). Here, the SRCAP-CFDP1 holoenzyme is bound to a hexasome in which not only ∼60 bp of DNA are unwrapped but also the histone H2A-H2B dimer is absent (Fig. 4, A and B, and movie S3). The nucleosome samples used for cryo-EM analysis contained only full nucleosomes and not preassembled hexasomes (figs. S2C, S6, and S7, and Materials and Methods), and the evicted state was observed exclusively in the combined presence of SRCAP and CFDP1, consistent with biochemical findings (Fig. 1D). This evicted state can also be observed in a distinct ATP-containing active-reaction sample, indicating that it is a truly functional reaction intermediate (“dataset 12 state 1,” figs. S12A and S16H, and Materials and Methods).

In the evicted state, the hexasome intermediate is drawn even closer to the RUVBL1/2 core and remains tightly bound by the SRCAP-CFDP1 holoenzyme through interactions with the SRCAP^ATPase^, CFDP1, ARP6, and ZNHIT1 subunits (Fig. 4, A and B). The ZNHIT1 “dyad helix,” critical for nucleosome engagement (fig. S30B), is sterically displaced and absent from this structure, and ARP6 forms new interactions with the hexasome, with ARP6 K^33^ and R^36^ inserting into the DNA major groove near SHL−1 (fig. S30D). Notably, the de novo generated hexasome DNA is unwrapped to a far greater extent than a free hexasome (fig. S30A). The CFDP1 BCNT-III helix and ZNHIT1 HIT-type zinc finger each contact different copies of H3 and H4 to cooperatively stabilize the H3-H4 tetramer, which has lost stabilizing interactions with nucleosomal DNA and H2A-H2B dimer (Fig. 4, B to D; fig. S30C; and movie S3). These interactions likely safeguard the integrity of the hexasome reaction intermediate to prevent errant disassembly of the entire nucleosome substrate.

### Conformational transitions of SRCAP-CFDP1 coordinate extensive DNA unwrapping with H2A-H2B eviction

Before CFDP1 binding, the SRCAP^ATPase^ in the fully engaged SRCAP-nucleosome structure adopts an open ATPase conformation (*39*–*43*) despite the unambiguous presence of ATPγS (fig. S30G). In contrast, for the evicted state of the ternary SRCAP-CFDP1-nucleosome complex, the SRCAP^ATPase^, also in the presence of ATPγS, now assumes a closed conformation and tightly clamps onto nucleosomal DNA (fig. S30G). Unlike other chromatin remodelers for which ATP binding is sufficient to drive the open-to-closed conformation (*39*–*43*), SRCAP^ATPase^ additionally requires conformational activation by CFDP1 to adopt the closed state. Aligning the activated (open^ATPase^) and evicted (closed^ATPase^) states relative to the histone octamer reveals the extraordinary motion of SRCAP^ATPase^-driven DNA unwrapping from the histone core (Fig. 4, E and F). Mechanistically, SRCAP-CFDP1 nucleosome engagement in the activated state initiates unwrapping of ∼20 bp of DNA through the ARP6-ZNHIT1 wedge (Figs. 2, A and C, and 4E), while the ensuing conformational change of the SRCAP^ATPase^ in the evicted state unwraps an additional ∼40 bp (i.e., 60 bp in total) to fully expose the H2A-H2B dimer for eviction (Fig. 4, E and F, and movie S3).

The conformational change of the SRCAP^ATPase^ in the activated to evicted state transition is transmitted to the ARP6-ZNHIT1 wedge through the RUVBL1/2 heterohexamer, causing the wedge to be driven further in toward the nucleosome. This stabilizes the interaction between ARP6 and the histone H3 N terminus and repositions the H3 N-terminal α helix toward the histone octamer core (Fig. 4G, fig. S30H, and movie S3). Strikingly, when superimposed on a canonical histone octamer structure, the restructured H3 αN helix is sterically incompatible with the evicted H2A-H2B dimer (Fig. 4G, inset). This H3-ARP6 interaction occurs primarily through insertion of H3 Y41E into a hydrophobic pocket on the ARP6 surface (fig. S31A). To investigate the importance of this interaction, we generated mutant nucleosomes (H3_Y41E) and tested histone exchange activity (fig. S31, B and C). SRCAP had substantially lower histone exchange activity on H3 mutant nucleosomes [22% compared to 51% for wild type (WT)] (fig. S31, B and C). These results suggest that SRCAP-CFDP1 holoenzyme restructuring of the H3 αN helix physically nudges the H2A-H2B dimer from the histone core, thereby coupling histone eviction with DNA unwrapping (movie S3).

A small subset of particles from the initial active-reaction cryo-EM data adopt an unwrapping intermediate for which dynamic DNA unwrapping is captured in action (“dataset 5 state 3”; Fig. 4E; and figs. S9A, S15F, and S31, D and E). While low resolution precludes atomic modeling, we could nonetheless use rigid body docking to visualize the SRCAP HSA module tracking with the unwrapped nucleosomal DNA as the H2A-H2B dimer is exposed before eviction, suggesting that the HSA module could contribute to DNA unwrapping once SRCAP becomes activated by CFDP1 and ATP (fig. S31, D and E, and movie S3).

### YL1 subunit of SRCAP chaperones H2A.Z-H2B dimer onto the hexasome

**Fig. 5.**
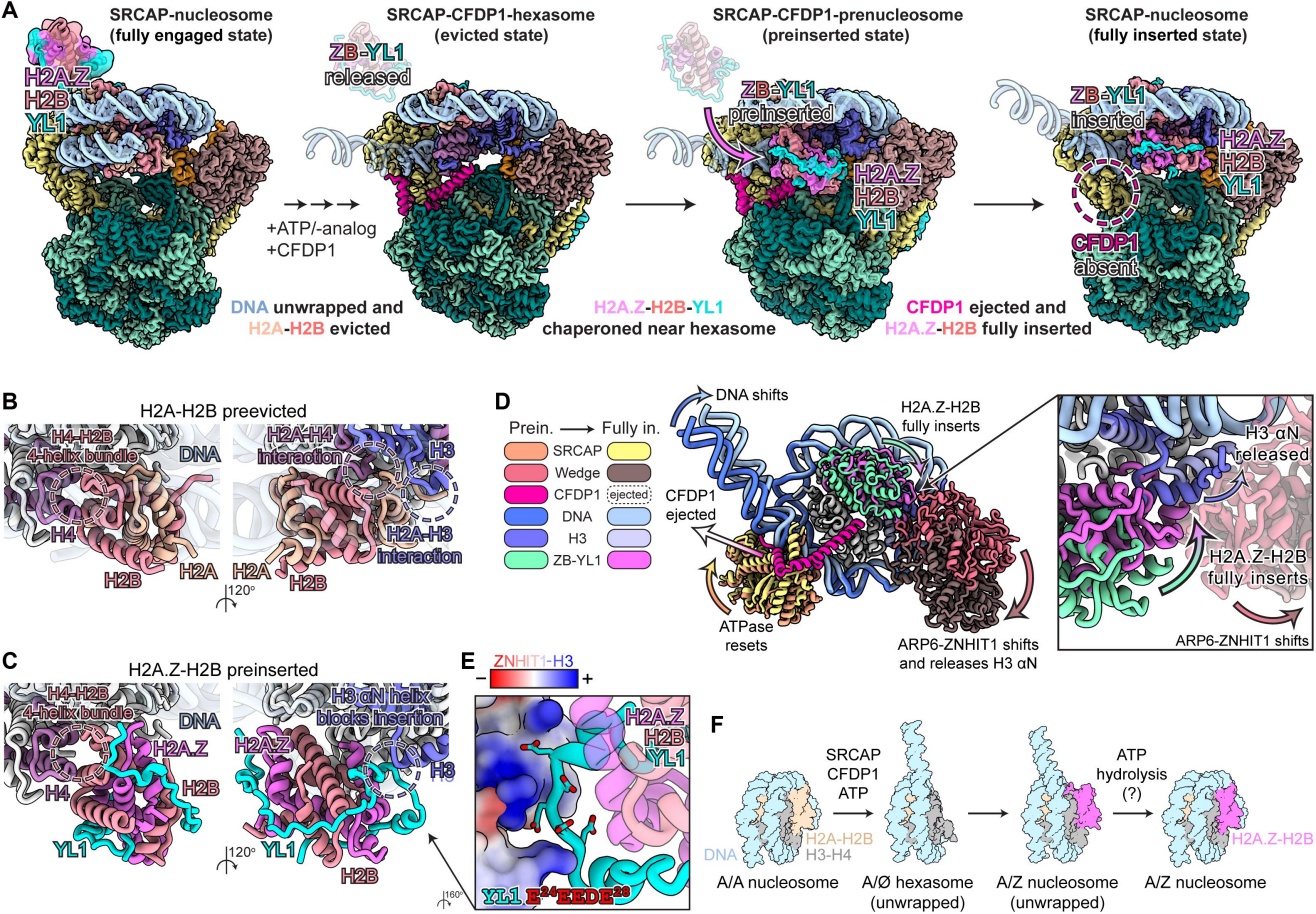
H2A.Z-H2B insertion into hexasome requires CFDP1 dissociation. (**A**) Cryo-EM maps (transparent) and fitted atomic models of SRCAP in the engaged, evicted, preinserted, and fully inserted states. For the engaged state, the cryo-EM density is a composite map of the H2A.Z-H2B density and high-resolution SRCAP core density. The SRCAP pre-CFDP1 engaged state corresponds to the state with density for the endogenous H2A.Z-H2B-YL1 subcomplex positioned proximal to the SRCAP^ATPase^. The SRCAP^ATPase^ proximal H2A.Z-H2B-YL1 density is not observed in the evicted state and reobserved in the preinserted state. CFDP1 is absent in the fully inserted state, and the H2A.Z-H2B-YL1 subcomplex is fully docked to the hexasome to generate a heterotypic A/Z nucleosome. (**B**) Histone-histone contacts of the H2A-H2B dimer before eviction (canonical histone octamer structure). The dimer is stabilized by the H4-H2B 4-helix bundle on one side and by the H2A C-terminal interactions with H3 and H4 on the other side. (**C**) Histone-histone contacts of the H2A.Z-H2B dimer in the preinserted state. The dimer only forms the H4-H2B 4-helix bundle interactions and the H2A.Z C terminus is sterically blocked by YL1 and ARP6-restructured H3 αN helix. (**D**) Conformational transition of SRCAP^ATPase^, ARP6-ZNHIT1 wedge, CFDP1, DNA, H3, and H2A.Z-H2B-YL1 subcomplex in the preinserted and fully inserted states (aligned to histone octamer). The inset shows a zoom-in view of H3 αN helix release upon CFDP1 dissociation and subsequent full insertion of H2A.Z-H2B-YL1. (**E**) Positive patch of ZNHIT1 and H3 colored according to Coulombic electrostatic potential [red: −10 kcal/(mol·e), blue: +10 kcal/(mol·e)]. The acidic loop of YL1 is shown with negatively charged residues shown in stick representation. The chaperoned H2A.Z-H2B dimer is shown transparent for clarity. (**F**) Cartoon schematic of the nucleosome compositions observed in our structures (A/A nucleosome, unwrapped A/Ø hexasome, and unwrapped A/Z nucleosome) and the A/Z nucleosome product presumptively released upon ATP hydrolysis.

Further sorting of the evicted state revealed a subset of particles exhibiting a preinserted state, where the YL1 subunit chaperones the H2A.Z-H2B dimer near the hexasome, thus forming a prenucleosome (Fig. 5A, “dataset 1 state 7,” figs. S7A and S14C, Materials and Methods, and Supplementary Text). This structurally resolved H2A.Z-H2B dimer is a stoichiometric component of the native enzyme complex without the addition of exogenous dimer during sample preparation (Fig. 1B and figs. S3, A and B, and S32, B and C). The structurally distinct L1 loop of H2A.Z (*44*) and N terminus of YL1 allow us to differentiate the observed dimer as endogenous H2A.Z-H2B rather than the evicted H2A-H2B dimer (fig. S32, B and C, and Supplementary Text). In addition, while the engaged-state (dataset 1 state 5) contains density proximal to the SRCAP^ATPase^ corresponding to H2A.Z-H2B (Figs. 2I and 5A, and fig. S32A), this density is absent from this intermediate, suggesting that the dimer is first sequestered and then released in the conformational transition to the evicted state (Fig. 5A, fig. S32A, and movie S4).

In intact nucleosomes, each histone H2A-H2B dimer is stabilized through two primary histone-histone interactions with the H3-H4 tetramer (Fig. 5B and fig. S33A) (*4*), involving on one side the H2B-H4 four-helix bundle and on the opposite side the H2A C-terminal tail. In the preinserted state, the YL1-chaperoned H2A.Z αC helix adopts an extended conformation that impedes the interaction with H3 and H4 (fig. S33, C to J, and movie S4) (*30*–*32*, *45*). In addition, the ARP6-restructured H3 αN helix sterically hinders H2A.Z C-terminal interactions (Fig. 5C and movie S4). Consequently, the YL1-H2A.Z-H2B complex forms only a partial H2B-H4 four-helix bundle, adopting a tilted conformation prior to stable incorporation (Fig. 5C, fig. S33A, and movie S4). In this tilted state, an acidic loop of YL1 (E^24^EEDE^28^) interacts with a positively charged patch formed by ZNHIT1 and H3, likely guiding H2A.Z-H2B for precise insertion (Fig. 5E and movie S4). Further sorting of the cryo-EM density reveals three distinct positions of the H2A.Z-H2B dimer in the prenucleosome, potentially depicting the trajectory of insertion (fig. S34, A and B).

### CFDP1 dissociation is coupled to completion of H2A.Z-H2B insertion in histone octamer

In the same cryo-EM sample, we resolved the ensuing inserted state (“dataset 1 state 8” and figs. S7A and S14D), wherein the DNA is unwrapped similarly to the preinserted state, but CFDP1 is dissociated from the SRCAP complex, and the H2A.Z-H2B-YL1 subcomplex is fully inserted into the hexasome, thus forming a heterotypic A/Z nucleosome (Fig. 5, A and D, and movie S5). The dissociation of CFDP1 concurs with our biochemical data indicating labile interactions with the SRCAP-nucleosome complex. Upon CFDP1 dissociation, the SRCAP^ATPase^ and YL1 throttle helix revert to the open conformation observed in the engaged and poised states (Fig. 5D and fig. S34C). The ZNHIT1 dyad helix also returns to its prior position, engaging the nucleosomal DNA (fig. S30B). ARP6 relieves the steric occlusion by the histone H3 αN helix, permitting full insertion of the H2A.Z-H2B dimer into the hexasome to generate a heterotypic H2A.Z-H2B histone octamer in the variant nucleosome (Fig. 5, A and D, and movie S5).

With the full insertion of H2A.Z-H2B, H3 and H4 interactions compete against residues 23 to 40 of YL1, and the extended H2A.Z αC helix uncoils to establish interactions as observed for intact nucleosomes (fig. S33, I and J, and movie S5). While the dimer is partially stabilized through interactions with the histone core, the DNA is still unwrapped, and YL1 shields the positively charged DNA binding surface of the H2A.Z-H2B dimer (Fig. 5, A and D, and fig. S34D). These results illustrate that while CFDP1 association is critical for conformational activation of SRCAP for histone eviction, its dissociation is required for histone insertion to complete the histone exchange reaction.

### SRCAP-CFDP1 can unwrap DNA and evict H2A-H2B without ATP hydrolysis

**Fig. 6.**
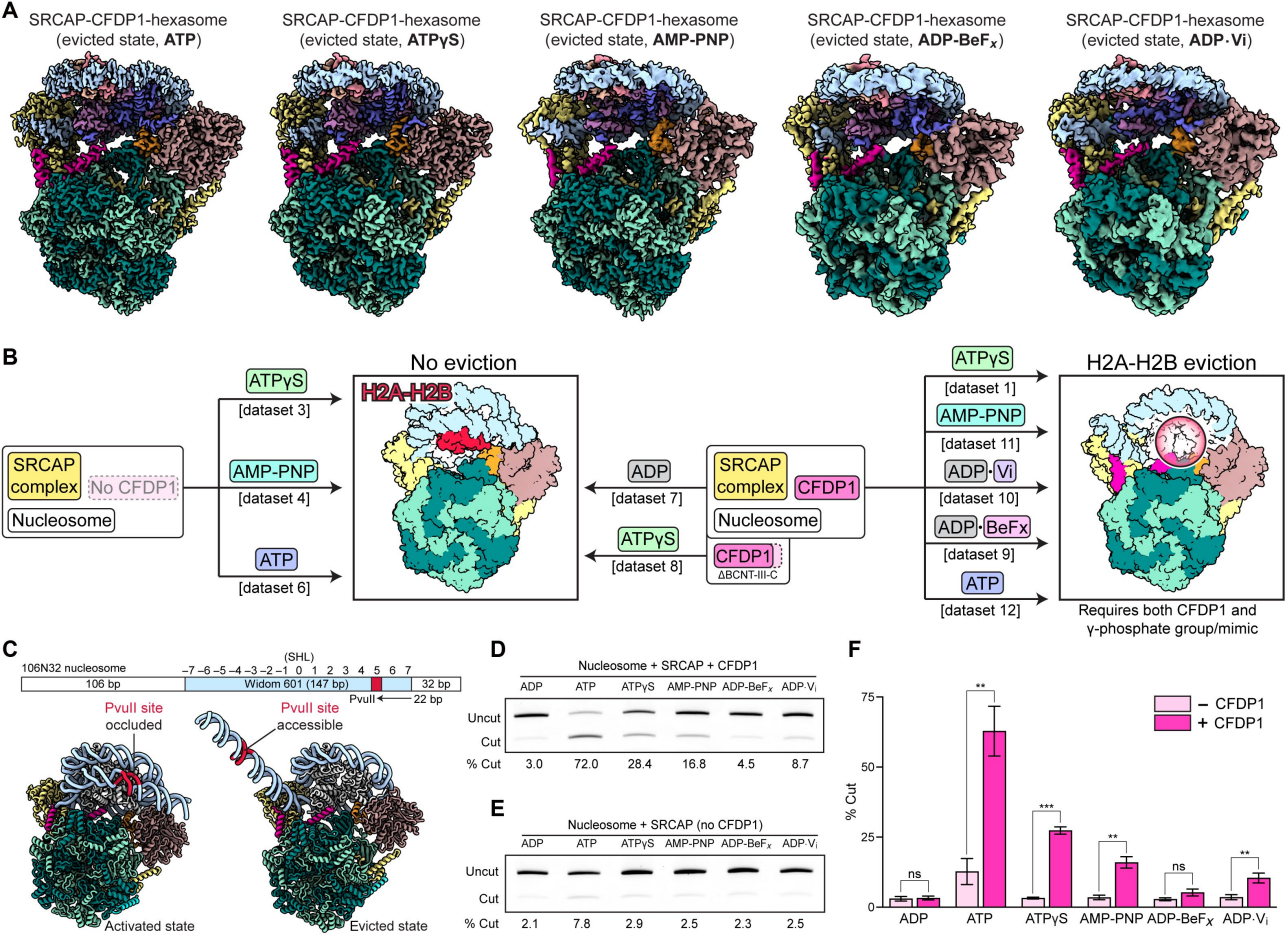
SRCAP-CFDP1 can unwrap DNA and evict H2A-H2B without ATP hydrolysis. (**A**) Cryo-EM maps of SRCAP-CFDP1-hexasome in the evicted state from five different cryo-EM samples assembled in the presence of either ATP, ATPγS, AMP-PNP, ADP·BeF*_x_*, or ADP·V_i_. (**B**) Diagram showing which cryo-EM datasets of indicated sample composition result in H2A-H2B eviction versus no eviction. The H2A-H2B dimer is colored red (no eviction) and the evicted H2A-H2B dimer is indicated with a transparent red sphere (‘H2A-H2B eviction’). Both full-length CFDP1 and the γ-phosphate group (or mimic) is required for H2A-H2B eviction. (**C**) Top: Schematic of the nucleosome used in the restriction enzyme assay wherein the restriction enzyme (PvuII) site is located near linker distal SHL5 of the nucleosome. Bottom: Position of the PvuII site mapped onto the structures of the activated and evicted states. (**D**) Restriction enzyme assay (see fig. S9 for replicates and controls) in the presence of nucleosomes, SRCAP, CFDP1, and various nucleotides. The top band corresponds to uncut DNA and the bottom band corresponds to PvuII cut DNA. Percent of cut DNA is indicated below each lane. (**E**) Restriction enzyme assay conducted as in Fig. 4C but without CFDP1. (**F**) Bar diagram showing PvuII digestion with and without CFDP1. Three technical replicates (*n* = 3) and the mean and SD are shown. Significance levels are represented as: **P ≤ 0.01 and ***P ≤ 0.001; ns, not significant. Statistical significance was calculated by two-tailed, unpaired *t* test with Welch’s correction; ADP ± CFDP1: ns, *P* = 0.6763; ATP ± CFDP1: **P = 0.0032; ATPγS ± CFDP1: ***P = 0.0005; AMP-PNP ± CFDP1: **P = 0.0038; ADP·BeF*_x_* ±CFDP1: ns, *P* = 0.0634 (ns); ADP·V_i_ ± CFDP1: **P = 0.0093.

We resolve the evicted state for the ternary complex assembled with ATPγS, a hydrolysis-resistant ATP analog that SRCAP is incapable of utilizing for in vitro histone exchange, even under extended reaction for 90 min at 37°C (far longer than the duration of cryo-EM sample preparation) (Fig. 6A and fig. S3D). Thus, the SRCAP-CFDP1 holoenzyme can apparently unwrap ∼60 bp of nucleosomal DNA and evict H2A-H2B without hydrolyzing bound ATP. We confirmed this finding with cryo-EM samples assembled in the presence of other nonhydrolyzable ATP analogs: ADP-BeF*_x_*, ADP-V_i_, and AMP-PNP (“datasets 9, 10, and 11”; Fig. 6, A and B; and figs. S11, A to C, S16, E to G, and S35, A and B).

Complexes assembled in the presence of ADP show no evicted states by cryo-EM, indicating that the γ-phosphate of ATP is required for the SRCAP^ATPase^ to adopt the necessary conformation for DNA unwrapping (“dataset 7”; Fig. 6B; and figs. S10B, S16, B and C, and S35, A and B). CFDP1 is also necessary for dimer eviction, as the binary SRCAP-nucleosome complex assembled in the presence of either ATP, ATPγS, or AMP-PNP yielded no evicted states, consistent with biochemical findings (“datasets 3, 4, and 6”; Fig. 6B; and figs. S8, B and C, S10A, S15, B and C, S16A, and S35, A and B). Furthermore, dimer eviction is dependent on the full BCNT domain, as the truncation of the last C-terminal α helix of CFDP1 does not inhibit ternary complex formation by the mutant protein but precludes advancement to the evicted state despite complex assembly with ATPγS (“dataset 8”; Fig. 6B; and figs. S10C, S16D, and S35, A and B).

To further investigate the role of ATP hydrolysis in DNA unwrapping, we designed a restriction digestion assay in which the restriction enzyme (PvuII) recognition site is located near SHL5 of the nucleosome where it is occluded or inaccessible before full DNA unwrapping by the SRCAP-CFDP1 holoenzyme (Fig. 6C). As expected, the nucleosome-only control shows a basal level of PvuII digestion (∼3%, likely attributed to a minor fraction of free DNA present in the nucleosome sample) (Fig. 6D). The addition of SRCAP without nucleotide (apo) or with ADP shows no increase in digestion (Fig. 6, D and F). However, ATP and ATP analogs ATPγS, AMP-PNP, ADP·BeF*_x_*, and ADP·V_i_ all show DNA unwrapping to different extents (Fig. 6, D and F). The addition of ATP to the reaction results in PvuII digestion of the majority of nucleosomes (∼63%), consistent with the histone exchange assay data (Fig. 6, D and F). ATPγS, AMP-PNP, and ADP·V_i_ also show a substantial fraction of PvuII digestion (27, 16, and 11%, respectively) over control (Fig. 6, D and F). CFDP1 is required for DNA unwrapping under all nucleotide conditions, in agreement with histone exchange biochemistry and cryo-EM data (Fig. 6, D to F, and figs. S35, A and B and S36, A to H). Thus, ATP hydrolysis–independent DNA unwrapping by the SRCAP-CFDP1 holoenzyme observed by cryo-EM can be verified by an orthogonal assay in solution. However, while we are able to observe the evicted state in the ADP·BeF*_x_*-assembled cryo-EM dataset (“dataset 9” and figs. S11A and S16E), the increase of PvuII digestion (5%) over the nucleosome control is small (Fig. 6, C to E), which could be attributed to inefficient BeF*_x_* complexation from NaF and BeSO_4_ precursors to yield the ATP mimic. In addition, while the other ATP analogs are sufficient for DNA unwrapping to some extent, the fraction of nucleosomes digested under these conditions are all lower compared to the reaction with ATP. Together, these results suggest that bulk nucleosomal DNA unwrapping for the 60-min restriction digestion reaction is enhanced by multiple cycles of ATP hydrolysis in the reaction.

### ATP hydrolysis is linked to SRCAP dissociation to allow DNA rewrapping

The final state we observe appears to represent a conformation just before SRCAP dissociation from the nucleosome (predissociated state; “dataset 1 state 9”; and figs. S7A, S14E, and S37, A and B). Here, ZNHIT1 is no longer engaged at the nucleosome dyad, and the unwrapped heterotypic nucleosome assumes a more distal position relative to the SRCAP core (fig. S37, A and B). As the primary remaining contact with the nucleosome is the catalytic SRCAP^ATPase^, we postulate that ATP hydrolysis is required for the final step of the histone exchange pathway—release of the catalytic SRCAP^ATPase^ subunit, and thus the entire complex, from DNA (Fig. 5F). The rewrapping of the nucleosomal DNA upon SRCAP^ATPase^ disengagement likely competes against the YL1-Z chaperone domain, allowing full dissociation of SRCAP from the nucleosome.

To biochemically test the role of ATP hydrolysis, we developed an ATP-chase dissociation assay in which the SRCAP-CFDP1-nucleosome ternary complex was assembled in the presence of low ATPγS (0.1 mM) and then challenged with ATP (1 mM) to induce disassembly (fig. S37C). As expected, SRCAP remained stably bound to the nucleosome in the presence of ATPγS and did not dissociate upon addition of excess lambda DNA, resulting in retention of SRCAP-nucleosome complex in the gel wells after nondenaturing gel electrophoresis (fig. S37, D and E, top). In contrast, the addition of ATP triggered rapid release of the nucleosome, as evidenced by its migration into the gel, indicating dissociation of SRCAP (fig. S37, D and E, bottom).

Thus, the requirement for ATP hydrolysis for efficient SRCAP dissociation helps explain the differences observed between our restriction digest and histone exchange assays. In the restriction digest assay, unwrapping—detected by increased DNA accessibility—requires SRCAP to generate and remain bound to the de novo hexasome but does not require its release from the substrate. In contrast, the histone exchange assay detects only free nucleosomes which migrate into the polyacrylamide gel and thus requires both histone exchange and subsequent SRCAP dissociation. Our cryo-EM datasets captured in the presence of hydrolysis-resistant ATP analogs (ATPγS, AMP-PNP, ADP·BeF*x*, and ADP·V_i_) further support this interpretation by revealing the evicted state in which SRCAP remains bound to the hexasome.

Collectively, our results support the following model: (i) ATP binding—but not hydrolysis—is sufficient for SRCAP-CFDP1–mediated DNA unwrapping and H2A-H2B eviction; (ii) ATP hydrolysis, while not strictly required for eviction, enhances the efficiency of DNA unwrapping through multiple rounds of binding and hydrolysis; and (iii) ATP hydrolysis is essential for SRCAP dissociation and completion of the histone exchange reaction. This provides an explanation for why ATPγS permits DNA unwrapping but does not support full histone exchange in our gel-based assay, since the exchanged nucleosome cannot be released without hydrolysis.

### Structural mechanism of histone H2A.Z exchange

**Fig. 7.**
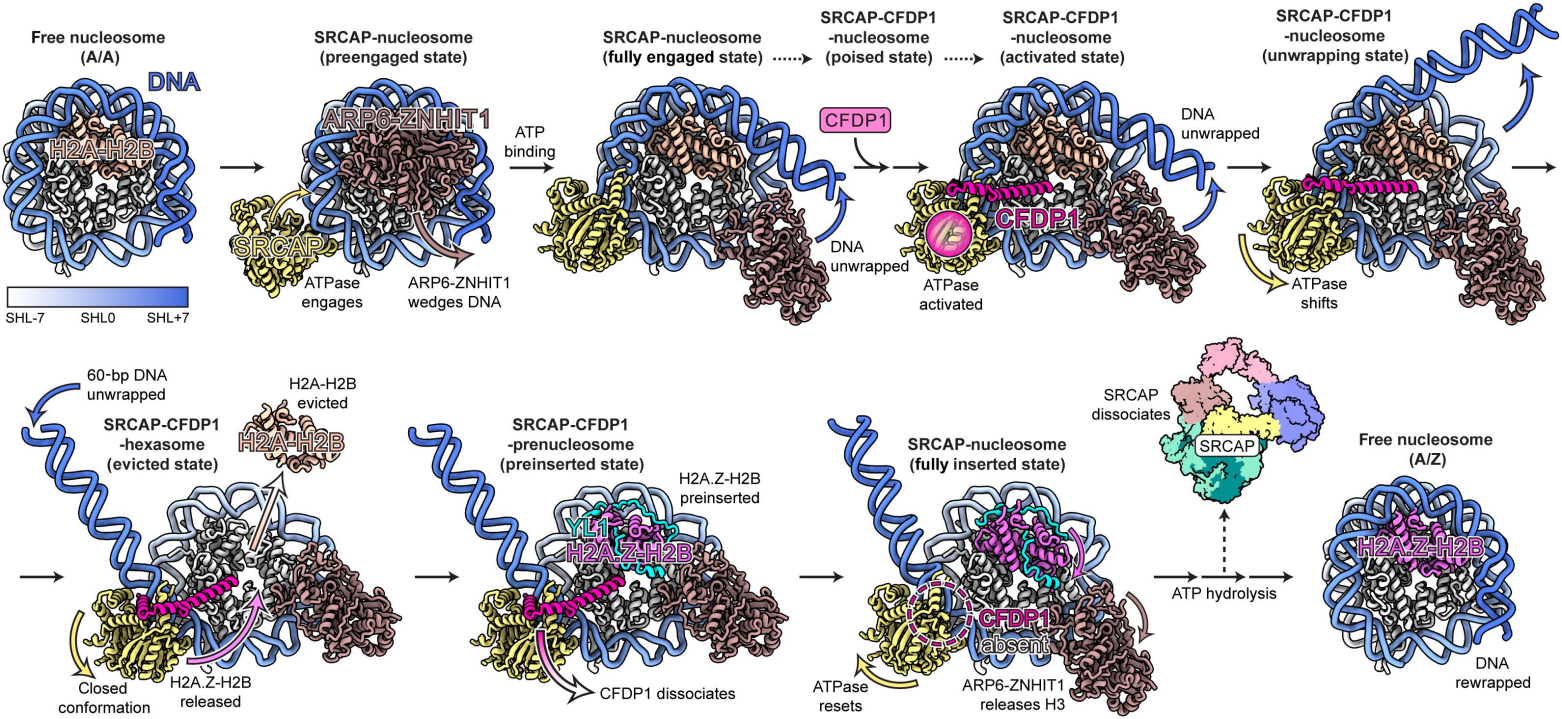
Mechanism of histone H2A.Z exchange through key reaction intermediates. Stepwise pathway of histone H2A.Z exchange by SRCAP-CFDP1 holoenzyme based on cryo-EM structures (free nucleosome, preengaged state, fully engaged state, activated state, unwrapping state, evicted state, preinserted state, and fully inserted state). The nucleosomal DNA is colored from SHL−7 to SHL+7 on a white to blue gradient. Some subunits are not shown for clarity. In the final step, we postulate, based on ensemble biochemistry, that ATP hydrolysis is required for SRCAP to dissociate from the heterotypic A/Z nucleosome and allow for either spontaneous or SRCAP mediated DNA rewrapping.

Collectively, our mechanistic analysis uncovers stepwise conformational transitions of the SRCAP-CFDP1 holoenzyme throughout the histone exchange pathway (Fig. 7 and movie S6). On initial nucleosome binding, ATP-bound SRCAP undergoes regulated transitions to nucleosome engagement in which the ARP6-ZNHIT1 wedge unwraps ∼20 bp of DNA. Once engaged, the SRCAP^ATPase^ adopts a conformation conducive to CFDP1 binding and holoenzyme formation. Subsequently, CFDP1 activates the SRCAP^ATPase^ to adopt the closed conformation with further nucleosome engagement of the holoenzyme, all in the context of bound ATP. Without ATP hydrolysis, the coordinated functions of the SRCAP^ATPase^, the linked HSA module, and ARP6 restructuring of H3 αN helix, concertedly unwrap an additional 40 bp of DNA and dislodge the exposed H2A-H2B dimer for eviction. The intermediate hexasome state is subsequently stabilized by CFDP1 and ZNHIT1, which protect the exposed H3-H4 tetramer. In addition, as SRCAP evicts the nucleosomal H2A-H2B dimer, the YL1-chaperoned H2A.Z-H2B dimer proximal to the SRCAP^ATPase^ is released and relocated near the hexasome, forming a prenucleosome with the restructured H3 αN helix preventing full H2A.Z-H2B dimer incorporation. Upon CFDP1 dissociation, ARP6 releases the H3 αN helix to permit full insertion of the H2A.Z-H2B dimer, but the DNA remains unwrapped and YL1 maintains protection of the DNA binding surface of the inserted dimer. Before SRCAP dissociation, the ZNHIT1 dyad helix detaches, leaving only the catalytic SRCAP^ATPase^ bound to the nascent heterotypic A/Z nucleosome. For the completion of the histone exchange cycle, the DNA rewraps and SRCAP lastly dissociates, a step we hypothesize requires the hydrolysis of the bound ATP.

## DISCUSSION

In this study, we report that human CFDP1 is an essential activator of the SRCAP complex that is strictly required for functional histone H2A.Z exchange in an in vitro reaction with purified components. We reveal the biochemical function of CFDP1 and elucidate at atomic resolution the molecular mechanism by which CFDP1 activates SRCAP. As CFDP1 does not copurify with SRCAP and only transiently interacts with the SRCAP-nucleosome complex during the histone exchange process, we conclude that the SRCAP apoenzyme, in the absence of CFDP1, represents the dormant or inactive state. This directly contrasts with an early claim (*13*) of histone exchange activity with the SRCAP apoenzyme immuno-purified from HeLa cells (under conditions that do not distinguish adventitious histone dimer association from true nucleosomal incorporation; see Supplementary Text).

Consistent with our SRCAP apoenzyme structure, previous structural and biophysical studies of the yeast SWR1 complex have shown that Arp6-Swc6 (yeast homologs of ARP6 and ZNHIT1) mediated partial DNA unwrapping independent of ATP hydrolysis (*28*, *29*, *33*). Notably, in contrast to our results and previous SWR1 studies, a recent study (*17*) reporting multiple cryo-EM structures of the SRCAP apoenzyme-nucleosome complex concluded that ATP hydrolysis is required for partial DNA unwrapping by ARP6-ZNHIT1 (further discussed in Supplementary Text).

Separately, a structure of the yeast SWR1 complex bound to a preformed hexasome to mimic an intermediate state was reported (fig. S38, A to G) (*46*). Several mechanistic differences are evident when compared to our human SRCAP-CFDP1 structures. The Swc5 BCNT-III helix is 10 amino acids longer than CFDP1 BCNT-III helix and proposed to be sterically incompatible with the evicted H2A-H2B dimer of the nucleosome (fig. S38D), whereas CFDP1 binds compatibly to both nucleosomes and hexasomes (fig. S38D). Yeast Arp6 also engages histone H3, but the conformation resembles the human activated state rather than the evicted state (fig. S38E) despite yeast SWR1 being bound to a preformed hexasome (fig. S38E). Last, the authors claim the conserved Swc5 BCNT-I domain interacts with the unwrapped DNA (fig. S38F). This is in direct conflict with previous biochemical (*47*) and structural (*29*) studies which report interaction with actin and Swr1 HSA helix. Here, we confirm that the conserved CFDP1 BCNT-I domain interacts with the homologous trident submodule composed of Baf53a and the HSA helix of SRCAP (fig. S38G).

The reported SWR1-hexasome structures (*46*) were derived from cryo-EM maps of limited interpretability (EMDB-18764, 18769, and 50297), with poorly defined density and low model validation scores, precluding detailed structural comparison. Moreover, as the authors used a biochemical mimic (SWR1 bound to preformed hexasome), there was no mechanistic insight into the central steps of histone exchange: DNA unwrapping, histone H2A-H2B eviction, or H2A.Z-H2B insertion. In contrast, our structures capture the human SRCAP-CFDP1 holoenzyme in multiple intermediate states, including the de novo generated hexasome as part of the bona fide histone exchange reaction. Thus, how the yeast SWR1 complex—which incorporates Swc5 (the yeast homolog of CFDP1) as a constitutive subunit—catalyzes H2A.Z exchange remains unclear, particularly given that CFDP1 acts as a transient activator rather than a stable component of human SRCAP complex.

Our results demonstrate that the SRCAP-CFDP1 holoenzyme can evict H2A-H2B without hydrolyzing ATP. This was confirmed with the use of four different ATP analogs: ATPγS, AMP-PNP, ADP-V_i_, and ADP-BeF*_x_*. Further, histone eviction is dependent not only on the gamma phosphate of the nucleotide but also the full BCNT domain of CFDP1. Collectively, our data indicate a model wherein 20 bp of DNA at SHL5 to SHL7 is initially unwrapped by the ZNHIT1-ARP6 wedge during nucleosome engagement, and this fully engaged state of SRCAP is specifically recognized by CFDP1, leading to holoenzyme formation. Subsequent CFDP1 activation drives the SRCAP^ATPase^ open to closed conformational transition, which applies a twist and strain on the nucleosomal DNA near SHL2, destabilizing histone-DNA contacts and detaching the remaining 40 bp of DNA for H2A-H2B eviction. This mechanism is distinct from previously proposed models of limited DNA translocation through multiple cycles of ATP hydrolysis (*5*, *17*, *28*, *34*, *40*, *48*, *49*). However, while not strictly required for histone dimer eviction, multiple rounds of ATP binding, hydrolysis, release, and rebinding are likely important for increasing the efficiency and productivity of DNA unwrapping. A similar mechanism has been observed for the related superfamily 2 DEAD-box proteins that can unwind RNA duplexes without ATP hydrolysis (*50*).

While SRCAP-CFDP1 can evict the histone dimer without hydrolyzing its bound ATP, the histone exchange cycle is not concluded, indicating that ATP hydrolysis is required for the final steps of DNA rewrapping and/or SRCAP dissociation. Long-lived DEAD-box family complexes in the presence of nonhydrolyzable ATP analogs have also been observed for DEAD-box helicases, Chd1, and SWR1 remodelers (*33*, *50*–*52*). In addition, chromatin remodelers have been shown to require ATP hydrolysis for efficient dissociation from chromatin in vivo (*53*, *54*). The elucidation of the mechanism of SRCAP dissociation and DNA rewrapping represent important areas for future investigation.

Our discovery of the dynamic activation of SRCAP by CFDP1 opens new avenues of research. CFDP1 does not copurify with endogenous SRCAP, even when exogenously added at high concentrations. By contrast, Swc5, the yeast homolog of CFDP1, also necessary for histone exchange, is stably integrated in the SWR1 complex (Supplementary Text) (*18*, *20*). This regulatory feature of CFDP1 is highly reminiscent of the transient association between general transcription factors TFIIA and TFIID, which recognize promoter elements in the assembly of the transcription preinitiation complex (*55*–*58*). TFIIA is not an integral part of the TFIID complex yet is required for ternary complex formation with promoter DNA (*55*–*58*). Similar to CFDP1, TFIIA has no other known function and does not stably associate with TFIID or DNA in the absence of the ternary complex (*55*–*58*). We postulate that dynamic cofactors such as TFIIA and CFDP1 have evolved as independent regulators through variations in expression levels, nuclear localization, and posttranslational modifications (*59*–*61*). CFDP1 is posttranslationally modified in vivo with phosphorylation sites clustered within the conserved BCNT domain, but the functional significance of this modification remains to be further explored (*62*, *63*).

Last, while our cryo-EM structures elucidate the fundamental mechanism of histone H2A.Z exchange on a canonical, unmodified nucleosome, questions remain regarding how other histone variants, histone posttranslational modifications, and higher-order chromatin structures may influence this process. In addition, gene-specific transcription factors, activators, and the myriad of nuclear processes occurring at promoter and enhancer regions may introduce additional layers of regulation for H2A.Z incorporation in vivo. These interactions with the nuclear milieu entail further studies to unravel the complex regulatory network of H2A.Z biology in health and disease.

## MATERIALS AND METHODS

### Generation of knock-in cell lines

Human K562 cells (American Type Culture Collection, #CCL-243) were cultured at 37°C and 5% CO2 in RPMI media supplemented with 10% fetal bovine serum and penicillin-streptomycin (10 U ml^−1^) and subcultured at a ratio of 1:10 to 1:15 every 2 to 4 days. WT K562 cells were cotransfected with a Cas9 plasmid [CBh-driven 3xFLAG-SV40NLS-hypaSpCas9-NLS; PGK-driven mVenus; U6-driven single-guide (sg) RNA] and a repair plasmid containing Halo-3xFLAG flanked by roughly 800 bp of genomic homology sequence to SRCAP on either side (4.5 μg of repair vector and 1.5 μg of Cas9 vector per T25 flask) using Lipofectamine 3000 (Thermo Fisher Scientific) according to the manufacturer’s protocol. The sgRNAs were designed using the Benchling CRISPR Guide RNA Design Tool (www.benchling.com/crispr), cloned into the Cas9 vector, and cotransfected with the repair vector individually. After 48 hours, transfected cells were combined and sorted using fluorescence-activated cell sorting for mVenus fluorescence. mVenus-sorted cells were grown for 7 days and labeled with 50 nM Halo-JF552, and cell populations with higher fluorescence than JF552-labeled WT cells were fluorescence-activated cell sort-selected and sorted individually into 96-well plates. Clones were expanded and genotyped by polymerase chain reaction (PCR). Successfully edited clones were further verified by PCR using multiple primer combinations, Sanger sequencing, and anti-FLAG immunoprecipitation.

### Preparative K562 cell culture and nuclei extraction

Large scale cultures of SRCAP-Halo-3xFLAG K562 cells were grown at 37°C and 5% CO_2_ in Opti-MEM media (Thermo Fisher Scientific) supplemented with 0.5% fetal bovine serum and 0.5% newborn calf serum. Cells were maintained in four 3-liter spinner flasks (Bellco), each containing 3 liters of K562 cultures and constantly stirred at 60 rpm via a Precision Magnetic Stirrer Platform (Bellco). Every 3 or 4 days, cells were split 1:15 or 1:30 into fresh media. Cells were collected when the cultures reached 5 × 10^5^ to 10 × 10^5^ cells/ml by centrifuging in a Fiberlite F9-6x1000 rotor (Thermo Fisher Scientific) at 4°C and 2500*g* for 15 min. The cell pellet was washed in phosphate-buffered saline (PBS) and then centrifuged at 4°C and 2500*g* for 5 min. Cells were resuspended in 5 volumes of ice-cold buffer A [10 mM Hepes (pH 7.6), 1.5 mM MgCl_2_, 10 mM KCl, 1 mM dithiothreitol (DTT), and 1× Roche cOmplete protease inhibitors], immediately pelleted (2500*g*, 4°C, 5 min), then resuspended in 3-volume ice-cold buffer A, and incubated on ice for 10 min (*64*). Cells were lysed by douncing 10 times using a glass homogenizer with a type B pestle. Nuclei were pelleted by centrifugation (3000*g*, 4°C, 20 min), and pellets were flash frozen in liquid nitrogen and stored at −80°C until use.

### Purification of native SRCAP complex

All steps were performed at 4°C. Frozen nuclei pellets from 50 to 100 liters of cell culture were thawed, resuspended in 0.9 volumes of buffer C [25 mM Hepes (pH 7.6), 0.2 mM EDTA, 25% glycerol, 0.42 M NaCl, 2 mM beta-mercaptoethanol (BME), 0.01% IGEPAL CA-630, 2× Roche cOmplete protease inhibitors, and 2× phosphatase inhibitors (1×: 1 mM NaF and 20 mM β-glycerophosphate)] and transferred to a 40-ml glass homogenizer. The nuclei were lysed by douncing with a type B pestle 20 times on ice. The nuclear lysate was nutated for 15 min and then centrifuged using a JA-20 Beckman rotor at 4°C and 18,000*g* for 30 min. The supernatant (nuclear extract) was collected, added to 4 ml of magnetic FLAG M2 resin (Sigma-Aldrich), and nutated for 4 hours at 4°C. The resin was washed twice with 10 column volumes (CV) of column buffer [25 mM Hepes (pH 7.6), 0.3 M NaCl, 1 mM MgCl_2_, 10% glycerol, 1 mM EDTA, 1 mM BME, 0.01% IGEPAL CA-630, 1× Roche cOmplete protease inhibitors, 1× phosphatase inhibitors), five times with 10 CV of wash buffer (column buffer containing 0.6 M NaCl), and twice with 10 CV of column buffer. To elute, the beads were incubated with Column Buffer with 3xFLAG peptide (0.5 mg ml^−1^) rocked for 1 hour and then pelleted, and this was repeated for four 1-hour elutions. Elutions were concentrated to 300 μl using a 30–kDa molecular weight cutoff Amicon concentrator (Sigma-Aldrich). The concentrated sample was loaded onto a 20 to 50% glycerol gradient [25 mM Hepes (pH 7.6), 0.3 M NaCl, 1 mM MgCl_2_, 0.2 mM EDTA, 1 mM DTT, and 0.01% IGEPAL CA-630] and ultracentrifuged using a SW55 Ti Beckman rotor at 4°C and 40,000 rpm for 20 hours. For the SRCAP complex used for cryo-EM dataset 1, recombinantly purified CFDP1 was added before glycerol gradient in an attempt to purify the SRCAP-CFDP1 holoenzyme. While CFDP1 did not stoichiometrically comigrate with SRCAP, there was a minor overlap between the peaks leading to substoichiometric levels of CFDP1 being present in the sample. This step was omitted for SRCAP purifications used for all other cryo-EM datasets and all biochemical experiments. Peak fractions of SRCAP complex, assessed by SDS-PAGE stained with Flamingo Fluorescent Stain (Bio-Rad), were pooled and concentrated using a 30–kDa molecular weight cutoff Amicon concentrator (Sigma-Aldrich). The concentrated sample was used immediately for cryo-EM or frozen in liquid nitrogen and stored at −80°C.

### CFDP1 expression and purification

The open reading frame of CFDP1 was subcloned into a modified pCAG vector. CFDP1 was N-terminally tagged with a C-myc NLS sequence and 3xFLAG tag followed by a 3C-protease recognition sequence. Expi293 cells were grown in Expi293 Expression medium (Gibco) to a density of roughly 2 × 10^6^ cells/ml at 37°C and 8% CO_2_. The plasmids were cotransfected using polyethylenimine (1 mg of total DNA and 3 mg of PEI (polyethyleneimine) per 1-liter culture). The transfected cells were cultured for 72 hours at 37°C and 8% CO_2_ and harvested by centrifugation. Nuclei were isolated as described for the K562 cells. All purification steps were performed at 4°C. Frozen nuclei from 1 liter of cell culture were thawed, 0.9 volumes of buffer C [25 mM Hepes (pH 7.6), 0.2 mM EDTA, 25% glycerol, 0.42 M NaCl, 2 mM BME, 0.01% IGEPAL CA-630, 2× Roche cOmplete protease inhibitors, and 2× phosphatase inhibitors (1×: 1 mM NaF and 20 mM β-glycerophosphate)] added and dounced using a glass homogenizer and a type B pestle 20 times on ice. The nuclear extract was nutated for 15 min and then centrifuged using a JA-20 Beckman rotor at 4°C and 18,000*g* for 30 min. The supernatant was collected and added to 2 ml of magnetic FLAG M2 resin (Sigma-Aldrich) for 4 hours nutating. The resin was washed twice with 10 CV of column buffer [25 mM Hepes (pH 7.6), 0.2 M NaCl, 10% glycerol, 1 mM EDTA, 1 mM BME, 0.01% IGEPAL CA-630, 1× Roche cOmplete protease inhibitors, and 1× phosphatase inhibitors] five times with 10 CV of wash buffer (column buffer containing 0.6 M NaCl), and twice with 10 CV of column buffer. To elute, the beads were incubated with column buffer with 3xFLAG peptide (0.5 mg ml^−1^) rocked for 1 hour and then pelleted, and this was repeated for four 1-hour elutions. Elutions were pooled and incubated with HRV 3C protease for 12 hours at 4°C. The sample was concentrated to 300 μl using a 10–kDa molecular weight cutoff Amicon concentrator (Sigma-Aldrich) and loaded onto a Superdex 75 Increase 10/300 GL size exclusion column. The peak fractions, assessed by SDS-PAGE stained with Flamingo Fluorescent Stain (Bio-Rad), were pooled and concentrated using a 10–kDa molecular weight cutoff Amicon concentrator (Sigma-Aldrich). The concentrated sample was frozen in liquid nitrogen and stored at −80°C.

WT and mutant CFDP1 proteins were additionally purified from NiCo21(DE3) (New England Biolabs) *Escherichia coli* cells. The codon-optimized sequence was synthesized as a gBlock (IDT) and cloned into the 1B plasmid (Addgene, #29653) for CFDP1 (WT), CFDP1 (L^247^D^248^W^249^→A^247^A^248^A^249^), and CFDP1 (K^219^R^220^→A^219^A^220^), and the 1*S* plasmid (Addgene, #29659) for CFDP1 (Δ1 to 42), CFDP1 (Δ95 to 120), CFDP1 (Δ162 to 198), CFDP1 (Δ224 to 232), CFDP1 (Δ236 to 299), and CFDP1 (Δ284 to 299). Cells were grown in TB (Terrific Broth) media at 37°C until optical density at 1.0, and the protein expression was induced with 0.4 mM isopropyl β-d-thiogalactopyranoside for 6 hours at 30°C. Cells were harvested and washed twice with PBS, and the pellet was flash-frozen in liquid nitrogen. All steps were performed at 4°C. The pellet was thawed and resuspended in lysis buffer [50 mM potassium phosphate (pH 8.0), 10 mM tris (pH 8.0), 500 mM NaCl, 10 mM imidazole, 2 mM BME, 0.1% Triton X-100, and 1× cOmplete protease inhibitor cocktail (Roche)]. Cell pellets were lysed by sonication and then clarified by centrifugation at 18,000*g* for 30 min. The clarified lysate was incubated with 2 ml of Ni–nitrilotriacetic acid (NTA) resin (QIAGEN) for 60 min on a rocking platform. The beads were washed five times with 10 CV of wash buffer A [50 mM potassium phosphate (pH 8.0), 500 mM NaCl, 20 mM imidazole, 2 mM BME, and 0.05% Triton X-100] and five times with 10 CV of wash buffer B [20 mM Hepes (pH 7.6), 150 mM NaCl, 20 mM imidazole, 2 mM BME, and 5% glycerol]. The protein was eluted with elution buffer (wash buffer B containing 280 mM imidazole). For 1*S* plasmid constructs, the 6xHis-Sumo tag was cleaved by addition of Ulp1 at 0.25 mg/ml and incubation for 4 hours. The Ni-NTA elutions were pooled then loaded onto a 1 ml of HiTrap Q HP column and eluted via salt gradient. The peak fractions were pooled, concentrated using a 10–kDa molecular weight cutoff Amicon concentrator (Sigma-Aldrich), and loaded onto a Superdex 75 Increase 10/300 GL size-exclusion column. The peak fractions, assessed by SDS-PAGE stained with Flamingo Fluorescent Stain (Bio-Rad), were pooled and concentrated using a 10–kDa molecular weight cutoff Amicon concentrator (Sigma-Aldrich). The concentrated sample was frozen in liquid nitrogen and stored at −80°C.

### Preparation of nucleosome substrates

Recombinant Xenopus histones H2A, H2B, H3, and H4 were expressed and purified as described previously (*65*). The nucleosomal DNA contained the upstream super core promoter (*66*) and downstream TERT promoter followed by a modified Widom 601 sequence and random linker sequence (*67*). The 285-bp DNA sequence was 5′- ATCGAAGGGCGCCTATATAAGGGGGTGGGGGCGCGTTCGTCCTCCCTCTCCTCGCGGCGCGAGTTTCAGGCAGCGCTGCGTCCTGCTGCGCACGTGGGAAGCCCTGCTGGAGAATCCCGGTGCGCAGGCCGCTCAATTGGTCGTAGACAGCTCTAGCACCGCTTAAACGCAGCTACGCGCTGTCCCCCGCGTTTTAACCGCCAAGGGGATTACTCCCTAGTCTCCAGGCAGCTGTCAGATATGTACATCCTGTGATCCCCGGGTACCGAGCTCGAATTCACTGGC-3′. The Widom 601 sequence is underlined. The DNA was generated by large-scale polymerase chain reaction, purified by anion exchange chromatography and ethanol precipitation. For nucleosome reconstitution, DNA and histone octamers were mixed in equimolar ratio and dialyzed against a gradient of decreasing salt concentration (*65*). Reconstituted nucleosomes were purified on a Model 491 Prep Cell (Bio-Rad), concentrated to 5 μM, and stored at 4°C.

Human histones were purchased from Histone Source (Colorado State University) and used to generate human nucleosomes for biochemical experiments. The nucleosomal DNA contained the Widom 601 sequence flanked by a random plasmid sequence of 60 bp on each side (*67*). The 267-bp DNA sequence was 5′-ATCGCATCGATCTTCACACCGAGTTCATCCCTTATGTGATGGACCCTATACGCGGCCGCCCTGGAGAATCCCGGTGCCGAGGCCGCTCAATTGGTCGTAGCAAGCTCTAGCACCGCTTAAACGCACGTACGCGCTGTCCCCCGCGTTTTAACCGCCAAGGGGATTACTCCCTAGTCTCCAGGCACGTGTCAGATATATACATCCTGTGCATGATATCGATCCCCGGGTACCGAGCTCGAATTCACTGGCCGTCGTTTTACAACGTCG-3′. The Widom 601 sequence is underlined. The forward primer contained a 5′ IRDye 800 modified (IDT) base for fluorescent detection. H3 mutant (Y41E) histones were purchased from Histone Source (Colorado State University) as histone octamers and used to generate nucleosome core particles (Widom 601) with Cy5 modification (IDT) for fluorescent detection. Human nucleosomes were reconstituted, purified, and stored identically as the Xenopus nucleosomes (*65*).

### Histone exchange assay

Histone exchange assays with human SRCAP were performed similar to previous SWR1 assays (*26*). Fifteen nanomolar nucleosomes (IRDye labeled or unlabeled), 2.5 nM SRCAP complex, 50 nM H2A.Z-H2B^3xF^ dimer, 1 mM ATP, and 100 nM CFDP1 were mixed in histone exchange buffer [25 mM Hepes (pH 7.6), 0.25 mM EGTA, 0.25 mM EDTA, 2.5 mM MgCl_2_, bovine serum albumin [BSA (0.25 mg/ml), 75 mM NaCl, 5% glycerol, 0.025% IGEPAL CA-630, and 0.25 mM tris(2-carboxyethyl)phosphine (TCEP)] and incubated at 37°C for the indicated times before quenching by addition of lambda DNA and transfer to ice. The reactions were then resolved on a 5% native polyacrylamide gel [0.5× Tris-borate-EDTA (TBE)] and scanned on a Typhoon biomolecular imager (Cytiva). For unlabeled nucleosomes (106N32), the gel was first stained with SYBR gold (Invitrogen) before scanning. To test for H2A.Z-H2B^3xF^ nonspecific/non-nucleosomal interactions, we added H2A.Z-H2B^3xF^ (0 to 50 nM) to nucleosome core particles (NCP), long linker nucleosomes (106N32), and long linker nucleosomes (106N32) in the presence of high salt (0.4 M NaCl). The samples were incubated for 15 min at 37°C and resolved as described above. Lambda phage DNA was necessary for preventing nonspecific/non-nucleosomal H2A.Z-H2B^3xF^ interactions.

### Three-color electrophoretic mobility shift assay

Eight nM nucleosomes (IRDye), 0.5 micromolar CFDP1 (Cy3-labeled), 25 nM human SRCAP (HaloTag), 1.0 μM JFX650 dye, and nucleotide (no nucleotide, 1 mM ADP, 1 mM ATPγS, or 1 mM ATP) were incubated in histone exchange buffer for 30 min at 37°C and resolved on a 1% native agarose gel (0.2× TB). The gel was scanned on a Typhoon biomolecular imager (Cytiva) for the IRDye (nucleosome), Cy3 (CFDP1), and JFX650 (SRCAP).

### Restriction enzyme assay

Twenty nanomolar nucleosomes (106N32), 35 nM SRCAP, and 500 nM CFDP1 were incubated for 5 min at 37°C in restriction enzyme buffer [25 mM Hepes (pH 7.6), 7.5 mM MgCl_2_, 75 mM NaCl, BSA (0.25 mg/ml), 5% glycerol, 0.025% IGEPAL CA-630, and 0.25 mM TCEP] in the presence of no nucleotide (apo), 1 mM ADP, 1 mM ATP, 1 mM ATPγS, 1 mM AMP-PNP, 1× ADP-BeF*_x_* (1 mM ADP, 3 mM BeSO_4_, and 80 mM NaF), or 1× ADP-V_i_ (1 mM ADP and 2 mM NaVO_3_). Then, 2 U of PvuII (Thermo Fisher Scientific, #ER0635) was added to the reaction and further incubated for another 1 hour at 37°C. One volume equivalent of proteinase K buffer [20 mM tris (pH 8.0), 10 mM EDTA, and 2% SDS] and 0.2 μl of proteinase K (Thermo Fisher Scientific, #EO0491) were added and incubated for 30 min at 55°C. The DNA was purified with the Monarch DNA Cleanup Kit (New England Biolabs, #T1030L) resolved on a 10% native polyacrylamide gel (0.5× TBE), stained with SYBR Gold stain (Thermo Fisher Scientific, #S11494), and scanned on a Typhoon biomolecular imager (Cytiva). CFDP1 was excluded for the no CFDP1 condition, and the assay was conducted as described above. We additionally tested the efficiency of PvuII to cut naked DNA in the presence of the various nucleotides used above with and without CFDP1 and saw no change, indicating that our results are not due to other confounding factors.

### ATP-chase dissociation assay

Fifteen nanomolar nucleosomes (IRDye labeled), 0 to 50 nM SRCAP complex, 0.1 mM ATPγS, and 100 nM CFDP1 were mixed in histone exchange buffer [25 mM Hepes (pH 7.6), 0.25 mM EGTA, 0.25 mM EDTA, 2.5 mM MgCl_2_, BSA (0.25 mg/ml), 75 mM NaCl, 5% glycerol, 0.025% IGEPAL CA-630, and 0.25 mM TCEP] and incubated at 37°C for 30 min to allow the formation of nucleosome-bound SRCAP complexes. Then, half of the reaction was transferred to a new tube and challenged with 1 mM ATP at 37°C for 3 min. Subsequently, both halves were quenched with addition of lambda DNA, transferred to ice, then resolved on a 5% native polyacrylamide gel (0.5× TBE), and scanned on a Typhoon biomolecular imager (Cytiva). In this assay, stable SRCAP-nucleosome complexes (even in the presence of lambda DNA) remain stuck in the well of the gel, whereas nucleosomes released upon ATP addition run into the gel. Thus, we can visualize the direct effect of ATP hydrolysis in SRCAP dissociation from the nucleosome. Notably, while this assay was not conducted with exogenous 3xFLAG-tagged H2A.Z-H2B dimer used for histone exchange assays, it did contain the endogenous untagged H2A.Z-H2B dimer that copurifies with SRCAP complex. To observe histone H2A.Z incorporation during SRCAP enzymatic activity and subsequent dissociation, we conducted the same assay with 3xHA-H2A nucleosomes. The eviction of 3xHA-tagged H2A-H2B dimer and the insertion of the untagged H2A.Z-H2B dimer lead to faster migration in a gel (opposite of the histone exchange assay conducted with untagged nucleosomes and 3xFLAG-tagged H2A.Z-H2B dimer). SRCAP dissociation occurred as part of the last step in the histone exchange pathway, as evidenced by H2A.Z exchanged nucleosomes that were released.

### Bioinformatic analysis of CFDP1

The ConSurf webserver (https://consurf.tau.ac.il) was used to analyze the evolutionary conservation of amino acids of human CFDP1 using the default parameters (*68*–*70*). An initial structure of CFDP1 was predicted with AlphaFold2 (*71*, *72*) run on the ColabFold server (https://colab.research.google.com/github/sokrypton/ColabFold/blob/main/AlphaFold2.ipynb) and then manually trimmed based on our cryo-EM structures. The ConSurf scores were then mapped to the structured domains of CFDP1. The N-terminal H2A-H2B chaperone domain of CFDP1 was predicted using AlphaFold2 multimer on the ColabFold server (*71*–*73*). The multiple sequence alignment graph was generated using ColabFold (*71*, *72*). The BCNT domain structural conservation of model organisms was predicted with AlphaFold2 run on the ColabFold server (*71*, *72*). BCNT domain sequence conservation of model organisms was aligned using the Clustal Omega web server (www.ebi.ac.uk/jdispatcher/) (*74*).

### Cryo-EM dataset 1—SRCAP-ATPγS-CFDP1-nucleosome

The SRCAP-ATPγS-106N32-CFDP1 sample (dataset 1) contained a substoichiometric concentration of CFDP1 due to a minor overlap of CFDP1 and SRCAP in glycerol gradient fractions. The concentrated peak fractions (1.4 μM native SRCAP, substoichiometric concentration of CFDP1), 106N32 nucleosomes (1.8 μM), and ATPγS (1.0 mM) were incubated for 10 min at room temperature (RT), transferred to ice, and then crosslinked with 0.05% glutaraldehyde (Electron Microscopy Sciences) for 3 min. The sample was applied to plasma-cleaned (Tergeo-EM, PIE Scientific) Quantifoil 0.6/1.0 grids at 4°C under 100% humidity then immediately blotted for 5 s at 5-N force and vitrified in liquid ethane using Vitrobot Mark IV (Thermo Fisher Scientific). Dataset 1 was collected on a Thermo Fisher G3i Titan Krios at 300 keV equipped with a K3 direct electron-counting camera (Gatan). Semi-automated data collection was done using the SerialEM data collection software (*75*). For dataset 1, 20,709 micrographs were collected at a nominal magnification of 22,500 (1.025 Å/pixel, super-resolution mode) at a defocus range of −0.8 to −1.6 μm. Each micrograph was fractionated into 64 frames with 4.0 total exposure time and a total electron dose of ∼50 e^−^/Å^2^.

Cryo-EM data were processed using RELION v4.0 (*76*). Movie frames were aligned using MotionCor2 (*77*) to correct for specimen motion and CTF parameters were estimated using Gctf (*78*). For dataset 1, 7,394,086 particles were picked with the Laplacian of Gaussian (LoG) picker, and 10,833,376 particles were picked using 2D class averages of free SRCAP. Both sets of particles were extracted and sorted extensively through multiple rounds of 3D classification (figs. S6 and S7). Through various sorting methods, nine states (free, preengaged, partially engaged, fully engaged, activated, evicted, preinserted, fully inserted, and predissociation) were determined (figs. S6 and S7). For the free state (dataset 1 state 1; fig. S13D), 37,293 particles were three dimensionally (3D) refined to 3.56 Å, and focused refinements of the core and ARP6/ZNHIT1 yielded resolutions of 3.41 and 3.66 Å. For the preengaged state (dataset 1 state 2 and fig. S13C), 28,381 particles were 3D refined to 4.04 Å, and focused refinements of the core, ARP6/ZNHIT1, HSA module, and nucleosome yielded resolutions of 3.66, 3.96, 7.61, and 5.34 Å. For the partially engaged state (dataset 1 state 3; fig. S13B), 24,701 particles were 3D refined to 3.92 Å, and focused refinements of the core, ARP6/ZNHIT1, and ATPase-nucleosome yielded resolutions of 3.53, 3.53, and 3.88 Å. For the fully engaged state (dataset 1 state 4; fig. S13A), 227,030 particles were 3D refined to 3.35 Å, and focused refinements of the core, ARP6/ZNHIT1, ATPase, and nucleosome yielded resolutions of 2.60, 3.16, 3.22, and 3.04 Å. The trident submodule, A submodule, and GAS41 submodule were 3D refined from a subset of particles to 3.84, 9.65, and 7.32 Å. For the activated state (dataset 1 state 5; fig. S14A), 4346 particles were 3D refined to 4.71 Å, and focused refinements of the RUVBL core, ARP6/ZNHIT1, ATPase, and nucleosome yielded resolutions of 4.12, 5.99, 6.48, and 4.71 Å. For the evicted state (dataset 1 state 6; fig. S14B), 43,233 particles were 3D refined to 3.44 Å, and focused refinements of the RUVBL core, ARP6/ZNHIT1, ATPase, and hexasome yielded resolutions of 3.22, 3.38, 3.63, and 3.50 Å. Focused 3D classification of the evicted state revealed three substates (dataset 1 state 7; fig. S14C) of the pre-inserted H2A.Z-H2B dimer that we term preinserted A, pre-inserted B, and preinserted C, with 6877, 2482, and 2156 particles, respectively. Focused refinement of prenucleosome (dataset 1 state 7, preinserted A, B, C; fig. S14C), yielded resolutions of 4.00, 4.35, 6.70, and 6.48 Å, respectively. For the fully inserted state (dataset 1 state 8; fig. S14D), 24,589 particles were 3D refined to 3.84 Å, and focused refinements of the RUVBL core, ARP6/ZNHIT1, ATPase, and nucleosome yielded resolutions of 3.50, 3.53, 3.70, and 3.80 Å. For the predissociation state (dataset 1 state 9; fig. S14E), 24,877 particles were 3D refined to 3.92 Å, and focused refinements of the RUVBL core, ARP6/ZNHIT1, ATPase, and nucleosome yielded resolutions of 3.50, 3.73, 3.80, and 3.66 Å. All resolutions reported were determined from gold-standard Fourier shell correlation (FSC) of 0.143 (*79*).

### Cryo-EM dataset 2—SRCAP-nucleosome (no nucleotide, apo)

For the SRCAP-106N32 sample (dataset 2), native SRCAP (1.0 μM) was incubated with 106N32 nucleosomes (1.7 μM) for 30 min at RT and 3 min at 37°C and then transferred to ice. The final buffer, glow discharging, crosslinking, and blotting conditions were identical as dataset 1. Dataset 2 was collected on a Thermo Fisher G3i Titan Krios at 300 keV equipped with a Falcon 4i direct electron detector (Thermo Fisher Scientific) and Selectris Energy Filter (Thermo Fisher Scientific). Automated data collection was done using the EPU software (Thermo Fisher Scientific). For dataset 2, 6724 micrographs were collected at a nominal magnification of 165,000 (0.726 Å/pixel) at a defocus range of −0.8 to −1.6 μm. Micrographs were recorded in Electron Event Representation (EER) mode (320 frames per second), with 2.84-s total exposure time and a total electron dose of ∼40 e^−^/Å^2^. Twenty-two EER frames were grouped into one fraction corresponding to an electron dose of ∼1 e^−^/Å^2^ per fraction.

Cryo-EM data were processed using RELION v4.0 (*76*). Movie frames were aligned using MotionCor2 (*77*) to correct for specimen motion and CTF parameters were estimated using Gctf (*78*). For dataset 2, 791,566 particles were picked with the LoG picker, and 1,051,835 particles were picked using 2D class averages of free SRCAP. Both sets of particles were extracted and sorted extensively through multiple rounds of 3D classification (fig. S8A). For the preengaged state (dataset 2 state 1; fig. S15A), 4625 particles were 3D refined to 8.76 Å. All resolutions reported were determined from gold-standard FSC of 0.143 (*79*).

### Cryo-EM dataset 3—SRCAP-ATPγS-nucleosome

For the SRCAP-ATPγS-106N32 sample (dataset 3), native SRCAP (1.0 μM) was incubated with 106N32 nucleosomes (1.7 μM) and ATPγS (1 mM) for 30 min at RT and 3 min at 37°C and then transferred to ice. The final buffer, glow discharging, crosslinking, and blotting conditions were identical as dataset 1. Dataset 3 was collected on a Thermo Fisher G3i Titan Krios at 300 keV equipped with a Falcon 4i direct electron detector (Thermo Fisher Scientific) and Selectris Energy Filter (Thermo Fisher Scientific). Automated data collection was done using the EPU software (Thermo Fisher Scientific). For dataset 3, 6417 micrographs were collected at a nominal magnification of 165,000 (0.726 Å/pixel) at a defocus range of −0.8 to −1.6 μm. Micrographs were recorded in EER mode (320 frames per second), with 2.84 s total exposure time and a total electron dose of ∼40 e^−^/Å^2^. Twenty-two EER frames were grouped into one fraction corresponding to an electron dose of ∼1 e^−^/Å^2^ per fraction.

Cryo-EM data were processed using RELION v4.0 (*76*). Movie frames were aligned using MotionCor2 (*77*) to correct for specimen motion and CTF parameters were estimated using Gctf (*78*). For dataset 3, 718,381 particles were picked with the LoG picker, and 981,281 particles were picked using 2D class averages of free SRCAP. Both sets of particles were extracted and sorted extensively through multiple rounds of 3D classification (fig. S8B). For the fully engaged state (dataset 3 state 1; fig. S15B), 13,297 particles were 3D refined to 3.69 Å, and focused refinements of core, ARP6/ZNHIT1, ATPase, and nucleosome yielded resolutions of 3.37, 3.50, 3.85, and 3.65 Å. All resolutions reported were determined from gold-standard FSC of 0.143 (*79*).

### Cryo-EM dataset 4—SRCAP-AMP-PNP-nucleosome

For the SRCAP-AMP-PNP-106N32 sample (dataset 4), native SRCAP (1.0 μM) was incubated with 106N32 nucleosomes (1.7 μM) and AMP-PNP (1 mM) for 30 min at RT and 3 min at 37°C and then transferred to ice. The final buffer, glow discharging, crosslinking, and blotting conditions were identical as dataset 1. Dataset 4 was collected on a Thermo Fisher G3i Titan Krios at 300 keV equipped with a Falcon 4i direct electron detector (Thermo Fisher Scientific) and Selectris Energy Filter (Thermo Fisher Scientific). Automated data collection was done using the EPU software (Thermo Fisher Scientific). For dataset 4, 5513 micrographs were collected at a nominal magnification of 130,000 (0.925 Å/pixel) at a defocus range of −0.8 to −1.6 μm. Micrographs were recorded in EER mode (320 frames per second), with 3.31-s total exposure time and a total electron dose of ∼40 e^−^/Å^2^. Twenty-nine EER frames were grouped into one fraction corresponding to an electron dose of ∼1 e^−^/Å^2^ per fraction.

Cryo-EM data were initially processed with CryoSPARC v4.2.1 (*80*) to template pick particles and then transferred for further processing on RELION v4.0 (*76*). Movie frames were aligned to correct for specimen motion (*76*). The CTF parameters were estimated using CTFFIND4 (*81*). A total of 619,446 particles from CryoSPARC were extracted on RELION and sorted extensively through multiple rounds of 3D classification (fig. S8C). For the fully engaged state (dataset 4 state 1; fig. S15C), 10,770 particles were 3D refined to 3.86 Å, and focused refinements of core, ARP6/ZNHIT1, ATPase, and nucleosome yielded resolutions of 3.35, 3.95, 3.95, and 3.74 Å. All resolutions reported were determined from gold-standard FSC of 0.143 (*79*).

### Cryo-EM dataset 5—SRCAP-ATP-CFDP1-H2A.Z-H2B-nucleosome (*t*_short_)

For the SRCAP-ATP-106N32-CFDP1-H2A.Z-H2B sample (dataset 5), native SRCAP (0.9 μM) was first incubated with H2A.Z-H2B (1.0 μM) for 10 min at RT and then with 106N32 nucleosomes (2.0 μM) and ATP (1.0 mM) for 15 min at RT. Last, CFDP1 (2.0 μM) was added and incubated for 30 s at 37°C, and glutaraldehyde (Electron Microscopy Sciences) was added to a final concentration of 0.05% and immediately transferred to ice. The final buffer was 20 mM Hepes (pH 7.6), 0.25 mM EDTA, 1.5 mM MgCl_2_, 70 mM NaCl, 0.25 mM TCEP, and 4% glycerol. After crosslinking for 3 min on ice, the sample (3 μl) was applied to plasma-cleaned (Tergeo-EM, PIE Scientific) Quantifoil 0.6/1.0 grids at 4°C under 100% humidity. The sample was immediately blotted for 5 s at 5-N force and vitrified in liquid ethane using Vitrobot Mark IV (FEI). Dataset 5 was collected on a Thermo Fisher G3i Titan Krios at 300 keV equipped with a K3 direct electron-counting camera (Gatan). Semi-automated data collection was done using the SerialEM data collection software (*75*). For dataset 5, 9161 micrographs were collected at a nominal magnification of 22,500 (1.025 Å/pixel, super-resolution mode) at a defocus range of −0.8 to −1.6 μm. Each micrograph was fractionated into 64 frames with 4.0 total exposure time and a total electron dose of ∼50 e^−^/Å^2^.

Cryo-EM data were processed using RELION v4.0 (*76*). Movie frames were aligned using MotionCor2 (*77*) to correct for specimen motion and CTF parameters were estimated using Gctf (*78*). For dataset 5, 2,239,017 particles were picked with the LoG picker, and 3,730,973 particles were picked using 2D class averages of free SRCAP. Both sets of particles were extracted and sorted extensively through multiple rounds of 3D classification (fig. S9A). For the poised state (dataset 5 state 1; fig. S15D), 22,651 particles were 3D refined to 3.80 Å, and focused refinements of the RUVBL core, ARP6/ZNHIT1, ATPase, nucleosome, and trident module yielded resolutions of 3.50, 4.00, 4.25, 4.35, and 7.76 Å. For the activated state (dataset 5 state 2; fig. S15E), 37,606 particles were 3D refined to 3.84 Å, and focused refinements of the RUVBL core, ARP6/ZNHIT1, ATPase, nucleosome, and trident module yielded resolutions of 3.56, 3.80, 4.35, 4.21, and 7.91 Å. For the unwrapping state (dataset 5 state 3; fig. S15F), 1827 particles were 3D refined to 8.07 Å. All resolutions reported were determined from gold-standard FSC of 0.143 (*79*).

### Cryo-EM dataset 6—SRCAP-ATP-nucleosome (no CFDP1)

For the SRCAP-ATP-106N32 sample (dataset 6), native SRCAP (0.9 μM) was incubated with 106N32 nucleosomes (2.0 μM) and ATP (1.0 mM) for 10 min at RT and 3 min at 37°C and then transferred to ice. The final buffer was 25 mM Hepes (pH 7.6), 0.2 mM EDTA, 1.1 mM MgCl_2_, 70 mM NaCl, 0.25 mM TCEP, and 4% glycerol. The sample (3 μl) was crosslinked with 0.05% glutaraldehyde (Electron Microscopy Sciences) for 3 min on ice and then applied to plasma-cleaned (Tergeo-EM, PIE Scientific) Quantifoil 0.6/1.0 grids at 4°C under 100% humidity. The sample was immediately blotted for 5 s at 5-N force and vitrified in liquid ethane using Vitrobot Mark IV (FEI). Dataset 6 was collected on a Thermo Fisher G3i Titan Krios at 300 keV equipped with a K3 direct electron-counting camera (Gatan). Semi-automated data collection was done using the SerialEM data collection software (*75*). For dataset 3, 7538 micrographs were collected at a nominal magnification of 22,500 (1.025 Å/pixel, super-resolution mode) at a defocus range of −0.8 to −1.6 μm. Each micrograph was fractionated into 64 frames with 4.0-s total exposure time and a total electron dose of ∼50 e^−^/Å^2^.

Cryo-EM data were processed using RELION v4.0 (*76*). Movie frames were aligned using MotionCor2 (*77*) to correct for specimen motion and CTF parameters were estimated using Gctf (*78*). For dataset 6, 1,991,605 particles were picked with the LoG picker, extracted, and sorted extensively through multiple rounds of 3D classification (fig. S10A). For the engaged state (dataset 6 state 1; fig. S16A), 17,392 particles were 3D refined to 4.77 Å. All resolutions reported were determined from gold-standard FSC of 0.143 (*79*).

### Cryo-EM dataset 7—SRCAP-ADP-CFDP1-nucleosome

For the SRCAP-ADP-106N32-CFDP1 sample (dataset 7), native SRCAP (1.2 μM) was incubated with 106N32 nucleosomes (1.7 μM), ADP (1.0 mM), and CFDP1 (2.5 μM) for 15 min at RT and 3 min at 37°C and then transferred to ice. The final buffer was 20 mM Hepes (pH 7.6), 0.3 mM EDTA, 60 mM NaCl, 0.25 mM TCEP, and 3% glycerol. 3 μl of the sample was crosslinked with 0.05% glutaraldehyde (Electron Microscopy Sciences) for 3 min on ice then applied to plasma-cleaned (Tergeo-EM, PIE Scientific) Quantifoil 0.6/1.0 grids at 4°C under 100% humidity. The sample was immediately blotted for 4 s at 1-N force and vitrified in liquid ethane using Vitrobot Mark IV (FEI). Dataset 7 was collected on a Thermo Fisher G3i Titan Krios at 300 keV equipped with a Falcon 4i direct electron detector (Thermo Fisher Scientific) and Selectris Energy Filter (Thermo Fisher Scientific). Automated data collection was done using the EPU software (Thermo Fisher Scientific). For dataset 7, 7721 micrographs were collected at a nominal magnification of 165,000 (0.726 Å/pixel) at a defocus range of −0.8 to −1.6 μm. Micrographs were recorded in EER mode (320 frames per second), with 2.84-s total exposure time and a total electron dose of ∼40 e^−^/Å^2^. Twenty-two EER frames were grouped into one fraction corresponding to an electron dose of ∼1 e^−^/Å^2^ per fraction.

Cryo-EM data were processed using RELION v4.0 (*76*). Movie frames were aligned to correct for specimen motion (*76*). The CTF parameters were estimated using CTFFIND4 (*81*). For dataset 7, 870,961 particles were picked with the LoG picker, and 1,208,297 particles were picked using 2D class averages of free SRCAP. Both sets of particles were extracted and sorted extensively through multiple rounds of 3D classification (fig. S10B). For the poised state (dataset 7 state 1; fig. S16B), 4206 particles were 3D refined to 4.33 Å, and focused refinements of the RUVBL core, ARP6/ZNHIT1, ATPase, and nucleosome yielded resolutions of 3.85 Å, 4.27 Å, 6.74 Å, and 6.87 Å. For the activated state (dataset 7 state 2; fig. S16C), 2896 particles were 3D refined to 4.55 Å, and focused refinements of the RUVBL core, ARP6/ZNHIT1, ATPase, and nucleosome yielded resolutions of 4.12, 4.74, 5.74, and 5.84 Å. All resolutions reported were determined from gold-standard FSC of 0.143 (*79*).

### Cryo-EM dataset 8—SRCAP-ATPγS-CFDP1(Δ284 to 299)-nucleosome

For the SRCAP-ATPγS-106N32-CFDP1(Δ284 to 299) sample (dataset 8), native SRCAP (1.2 μM) was incubated with 106N32 nucleosomes (1.7 μM), ATPγS (1.0 mM), and CFDP1(Δ284–299) (3.5 μM) for 15 min at RT and 3 min at 37°C and then transferred to ice. The final buffer was 20 mM Hepes (pH 7.6), 0.3 mM EDTA, 60 mM NaCl, 0.25 mM TCEP, and 3% glycerol. The sample (3 μl) was crosslinked with 0.05% glutaraldehyde (Electron Microscopy Sciences) for 3 min on ice then applied to plasma-cleaned (Tergeo-EM, PIE Scientific) Quantifoil 0.6/1.0 grids at 4°C under 100% humidity. The sample was immediately blotted for 4 s at 1-N force and vitrified in liquid ethane using Vitrobot Mark IV (FEI). Dataset 8 was collected on a Thermo Fisher G3i Titan Krios at 300 keV equipped with a Falcon 4i direct electron detector (Thermo Fisher Scientific) and Selectris Energy Filter (Thermo Fisher Scientific). Automated data collection was done using the EPU software (Thermo Fisher Scientific). For dataset 8, 7715 micrographs were collected at a nominal magnification of 165,000 (0.726 Å/pixel) at a defocus range of −0.8 to −1.6 μm. Micrographs were recorded in EER mode (320 frames per second), with 2.84-s total exposure time and a total electron dose of ∼40 e^−^/Å^2^. Twenty-two EER frames were grouped into one fraction corresponding to an electron dose of ∼1 e^−^/Å^2^ per fraction.

Cryo-EM data were processed using RELION v4.0 (*76*). Movie frames were aligned to correct for specimen motion (*76*). The CTF parameters were estimated using CTFFIND4 (*81*). For dataset 8, 931,219 particles were picked with the LoG picker, and 1,246,387 particles were picked using 2D class averages of free SRCAP. Both sets of particles were extracted and sorted extensively through multiple rounds of 3D classification (fig. S10C). For the poised state (dataset 8 state 1; fig. S16D), 12,028 particles were 3D refined to 3.61 Å, and focused refinements of the RUVBL core, ARP6/ZNHIT1, ATPase, and nucleosome yielded resolutions of 3.37, 3.37, 3.61, and 3.69 Å. All resolutions reported were determined from gold-standard FSC of 0.143 (*79*).

### Cryo-EM dataset 9—SRCAP-ADP-BeF*_x_*-CFDP1-nucleosome

For the SRCAP–ADP-BeF*_x_*–106N32–CFDP1 sample (dataset 9), native SRCAP (0.9 μM) was incubated with 106N32 nucleosomes (2.0 μM) and ADP-BeF*_x_* (1.0 mM ADP, 2.0 mM BeCl_2_, and 8.0 mM NaF) for 10 min at RT, and then CFDP1 (2.0 μM) was added and incubated for 3 min at 37°C before transfer to ice. The final buffer was 25 mM Hepes (pH 7.6), 0.3 mM EDTA, 2 mM MgCl_2_, 70 mM NaCl, 0.25 mM TCEP, and 4% glycerol. The sample (3 μl) was crosslinked with 0.05% glutaraldehyde (Electron Microscopy Sciences) for 3 min on ice then applied to plasma-cleaned (Tergeo-EM, PIE Scientific) Quantifoil 0.6/1.0 grids at 4°C under 100% humidity. The sample was immediately blotted for 5 s at 5-N force and vitrified in liquid ethane using Vitrobot Mark IV (FEI). Dataset 9 was collected on a Thermo Fisher G3i Titan Krios at 300 keV equipped with a K3 direct electron-counting camera (Gatan). Semi-automated data collection was done using the SerialEM data collection software (*75*). For dataset 9, 1999 micrographs were collected at a nominal magnification of 22,500 (1.025 Å/pixel, super-resolution mode) at a defocus range of −0.8 to −1.6 μm. Each micrograph was fractionated into 64 frames with 4.0-s total exposure time and a total electron dose of ∼50 e^−^/Å^2^.

Cryo-EM data were processed using RELION v4.0 (*76*). Movie frames were aligned using MotionCor2 (*77*) to correct for specimen motion and CTF parameters were estimated using Gctf (*78*). For dataset 9, 542,549 particles were picked with the LoG picker, extracted, and sorted extensively through multiple rounds of 3D classification (fig. S11A). For the evicted state (dataset 9 state 1; fig. S16E), 5889 particles were 3D refined to 7.32 Å. All resolutions reported were determined from gold-standard FSC of 0.143 (*79*).

### Cryo-EM dataset 10—SRCAP–ADP-V_i_–CFDP1–nucleosome

For the SRCAP–ADP-V_i_–106N32–CFDP1 sample (dataset 10), native SRCAP (1.2 μM) was incubated with 106N32 nucleosomes (1.9 μM), ADP-Vi (1.0 mM ADP and 1.0 mM Na_3_VO_4_), and CFDP1 (2.0 μM) for 15 min at RT and 3 min at 37°C and then transferred to ice. The final buffer was 20 mM Hepes (pH 7.6), 0.3 mM EDTA, 60 mM NaCl, 0.25 mM TCEP, and 2.5% glycerol. The sample (3 μl) was crosslinked with 0.05% glutaraldehyde (Electron Microscopy Sciences) for 3 min on ice then applied to plasma-cleaned (Tergeo-EM, PIE Scientific) Quantifoil 0.6/1.0 grids at 4°C under 100% humidity. The sample was immediately blotted for 4 s at 1-N force and vitrified in liquid ethane using Vitrobot Mark IV (FEI). Dataset 10 was collected on a Thermo Fisher G3i Titan Krios at 300 keV equipped with a Falcon 4i direct electron detector (Thermo Fisher Scientific) and Selectris Energy Filter (Thermo Fisher Scientific). Automated data collection was done using the EPU software (Thermo Fisher Scientific). For dataset 10, 9079 micrographs were collected at a nominal magnification of 165,000 (0.726 Å/pixel) at a defocus range of −0.8 to −1.6 μm. Micrographs were recorded in EER mode (320 frames per second), with 3.60-s total exposure time and a total electron dose of ∼40 e^−^/Å^2^. Twenty-seven EER frames were grouped into one fraction corresponding to an electron dose of ∼1 e^−^/Å^2^ per fraction.

Cryo-EM data were processed using RELION v4.0 (*76*). Movie frames were aligned to correct for specimen motion (*76*). The CTF parameters were estimated using CTFFIND4 (*81*). For dataset 10, 822,852 particles were picked with the LoG picker, and 970,087 particles were picked using 2D class averages of free SRCAP. Both sets of particles were extracted and sorted extensively through multiple rounds of 3D classification (fig. S11B). For the evicted state (dataset 10 state 1; fig. S16F), 4367 particles were 3D refined to 7.01 Å. All resolutions reported were determined from gold-standard FSC of 0.143 (*79*).

### Cryo-EM dataset 11—SRCAP-AMP-PNP-CFDP1-nucleosome

For the SRCAP–AMP-PNP–106N32–CFDP1 sample (dataset 11), native SRCAP (1.2 μM) was incubated with 106N32 nucleosomes (1.9 μM), AMP-PNP (1.0 mM), and CFDP1 (2.0 μM) for 15 min at RT and 3 min at 37°C and then transferred to ice. The final buffer was 20 mM Hepes (pH 7.6), 0.3 mM EDTA, 60 mM NaCl, 0.25 mM TCEP, and 2.5% glycerol. The sample (3 μl) was crosslinked with 0.05% glutaraldehyde (Electron Microscopy Sciences) for 3 min on ice then applied to plasma-cleaned (Tergeo-EM, PIE Scientific) Quantifoil 0.6/1.0 grids at 4°C under 100% humidity. The sample was immediately blotted for 4 s at 1-N force and vitrified in liquid ethane using Vitrobot Mark IV (FEI). Dataset 11 was collected on a Thermo Fisher G3i Titan Krios at 300 keV equipped with a Falcon 4i direct electron detector (Thermo Fisher Scientific) and Selectris Energy Filter (Thermo Fisher Scientific). Automated data collection was done using the EPU software (Thermo Fisher Scientific). For dataset 11, 5276 micrographs were collected at a nominal magnification of 130,000 (0.925 Å/pixel) at a defocus range of −0.8 to −1.6 μm. Micrographs were recorded in EER mode (320 frames per second), with 3.60-s total exposure time and a total electron dose of ∼40 e^−^/Å^2^. Twenty-seven EER frames were grouped into one fraction corresponding to an electron dose of ∼1 e^−^/Å^2^ per fraction.

Cryo-EM data were initially processed with CryoSPARC v4.2.1 (*80*) to template pick particles, then transferred for further processing on RELION v4.0 (*76*). Movie frames were aligned to correct for specimen motion (*76*). The CTF parameters were estimated using CTFFIND4 (*81*). A total of 134,226 particles from CryoSPARC were extracted on RELION and sorted extensively through multiple rounds of 3D classification (fig. S11C). For the evicted state (dataset 11 state 1; fig. S16G), 6010 particles were 3D refined to 3.99 Å, and focused refinements of the RUVBL core, ARP6/ZNHIT1, ATPase, and hexasome yielded resolutions of 3.74, 5.82, 5.75, and 6.34 Å. All resolutions reported were determined from gold-standard FSC of 0.143 (*79*).

### Cryo-EM dataset 12—SRCAP-ATP-CFDP1-nucleosome (*t*_long_)

For the SRCAP-ATP-106N32-CFDP1 sample (dataset 12), native SRCAP (1.2 μM) was incubated with 106N32 nucleosomes (1.9 μM), ATP (1.0 mM) for 15 min at RT, then CFDP1 (2.0 μM) was added and incubated for 3 min at 37°C before transfer to ice. The final buffer was 20 mM Hepes (pH 7.6), 0.3 mM EDTA, 60 mM NaCl, 0.25 mM TCEP, and 2.5% glycerol. 3 μl of the sample was applied to plasma-cleaned (Tergeo-EM, PIE Scientific) Quantifoil 0.6/1.0 grids at 4°C under 100% humidity without chemical crosslinking. The sample was immediately blotted for 4 s at 1-N force and vitrified in liquid ethane using Vitrobot Mark IV (FEI). Dataset 12 was collected on a Thermo Fisher G3i Titan Krios at 300 keV equipped with a Falcon 4i direct electron detector (Thermo Fisher Scientific) and Selectris Energy Filter (Thermo Fisher Scientific). Automated data collection was done using the EPU software (Thermo Fisher Scientific). For dataset 12, 20,133 micrographs were collected at a nominal magnification of 165,000 (0.726 Å/pixel) at a defocus range of −0.8 to −1.6 μm. Micrographs were recorded in EER mode (320 frames per second), with 3.16-s total exposure time and a total electron dose of ∼40 e^−^/Å^2^. Twenty-four EER frames were grouped into one fraction corresponding to an electron dose of ∼1 e^−^/Å^2^ per fraction.

Cryo-EM data were initially processed with CryoSPARC v4.2.1 (*80*) to template pick particles, then transferred for further processing on RELION v4.0 (*76*). Movie frames were aligned to correct for specimen motion (*76*). The CTF parameters were estimated using CTFFIND4 (*81*). A total of 621,110 particles from CryoSPARC were extracted on RELION and sorted extensively through multiple rounds of 3D classification (fig. S12A). For the evicted state (dataset 12 state 1; fig. S16H), 31,192 particles were 3D refined to 3.19 Å, and focused refinements of the RUVBL core, ARP6/ZNHIT1, ATPase, and hexasome yielded resolutions of 3.04, 3.09, 3.70, and 3.28 Å. For the inserted state (dataset 12 state 2; fig. S16I), 7008 particles were 3D refined to 4.02 Å, and focused refinements of the RUVBL core, ARP6/ZNHIT1, ATPase, and nucleosome yielded resolutions of 3.46, 4.30, 5.58, and 4.15 Å. All resolutions reported were determined from gold-standard FSC of 0.143 (*79*).

### Model building and refinement

Core atomic models comprising regions of cryo-EM refinements resolved to ≤5 Å were refined into composite cryo-EM maps. Composite maps were generated by combining focused-refined constituent cryo-EM maps postprocessed using DeepEMhancer (*82*). To remove overlapping densities at the interface of the constituent maps, tight masks were generated by docking rough atomic models into the focused-refined maps, converting the models into molecular density maps using the “molmap” function in ChimeraX (using resolution setting between 3 and 4 Å depending on the resolution of the focused map), and then generating mask volumes from these using the relion_mask_create function in relion. These masks were then applied to their corresponding focused constituent maps, and the masked constituent maps were individually aligned to a global consensus map (refined without a mask from the same data and filtered by local resolution) using the “fitmap” function in ChimeraX. At this point, the aligned constituent maps were visually inspected to ensure there were no overlapping densities between adjacent maps. The aligned constituent maps were then combined using the “volume add” function in ChimeraX to generate the composite map used for atomic model building refinement. Additional maps, e.g. those postprocessed by local-resolution filtering, were also used to guide model construction in some cases.

Initial models were largely constructed from AlphaFold2-predicted structures generated using the ColabFold AlphaFold2_mmseqs2 Notebook on Google Colab (https://colab.research.google.com/github/sokrypton/ColabFold/blob/main/AlphaFold2.ipynb) (*71*–*73*). The fully engaged state of the SRCAP-nucleosome complex exhibited the highest resolution and completeness, and thus, the atomic model for this structure was built and refined first and then used to generate starting models for the remaining structures, with starting model restraints enforced within PHENIX and ISOLDE to limit divergence from the starting model. Manual model modifications were performed in Coot (*83*), followed by interactive molecular dynamics flexible fitting (MDFF) using ISOLDE in Chimera X (*84*, *85*) and lastly real-space refinement in PHENIX (*86*). The final models were validated using MTriage (*87*) and MolProbity (*88*) within PHENIX.

Extended atomic models were built that additionally include lower resolution regions visible in the globally refined consensus maps for which the underlying molecules could be reliably identified and confidently orientated in the map via rigid docking of high-confidence AlphaFold predicted models. The extended model for the fully engaged state of the SRCAP-nucleosome complex is the most complete out of the models from this study and was built first. The salient feature of this model is the extended SRCAP HSA module structure including the BAF53a, actin, DMAP1, and GAS41 subunits. A starting model for this module was generated in AlphaFold 3 (*89*) using the AlphaFold server (https://alphafoldserver.com/; see fig. S23) and was initially fit into the map as three separate pieces: the trident submodule, A submodule, and GAS41 submodule. The AlphaFold3 models were then fit into the map with MDFF using ISOLDE, with distance and torsion restraints applied to restrict deviations from the starting model. A focused-refined map for the trident submodule was used to first fit this part of the HSA module before fitting the rest of the HSA into the globally refined consensus map. Other extensions included in the extended models were built the same way from AlphaFold3 starting models or, in the case of extended linker DNA segments, from B-form double-stranded DNA structures generated using the “Build Structure” tool in ChimeraX. All models were lastly real-space refined in PHENIX (*86*) with starting model restraints enforced. The extended model for the fully engaged state was used as a base for the other extended models generated for the preengaged, poised, activated, and unwrapping states. We did not generate extended models for other structural states that did not have appreciable interpretable density beyond their core atomic model.

### Figure generation

Figures were generated using UCSF ChimeraX (*85*, *90*), Adobe Illustrator, and GraphPad Prism.

### Statistics and reproducibility

Statistical analyses were performed using GraphPad Prism (v10.2.3). Quantified and statistically analyzed experiments were performed in three technical replicates (*n* = 3), and the mean and SD are shown. Significance levels are represented as: *P ≤ 0.05, **P ≤ 0.01 and ***P ≤ 0.001; ns, not significant. Statistical significance was calculated by two-tailed, unpaired *t* test with Welch’s correction. *P* values are reported in the figure legends.

## Supplementary Materials

### The PDF file includes:

Supplementary Text Figs. S1 to S46 Tables S1 to S8 References
