# Supplementary material for: Structural mechanism of histone H2A.Z exchange by human SRCAP-CFDP1 holoenzyme
Source: Sci Adv. 2026 Jul 31;12(31):eaei7728. doi: 10.1126/sciadv.aei7728 (PMC13426437; doi:10.1126/sciadv.aei7728)
Supplement: Supplementary file 1 — Supplementary Text Figs. S1 to S46 Tables S1 to S8 References [file sciadv.aei7728_sm.pdf]

Science Advances
AAAS

Supplementary Materials for

Structural mechanism of histone H2A.Z exchange by human SRCAP-CFDP1 holoenzyme

Giho Park *et al*.

Corresponding author: Carl Wu, wuc@jhu.edu; Robert K. Louder, rklouder@jhu.edu

Sci. Adv. **12**, eaei7728 (2026)
DOI: 10.1126/sciadv.aei7728

The PDF file includes:

Supplementary Text
Figs. S1 to S46
Tables S1 to S8
Legends for movies S1 to S6
References

Other Supplementary Material for this manuscript includes the following:

Movies S1 to S6

Supplementary Text

Free monomeric SRCAP

Prior to chromatin engagement, SRCAP appears to be in an auto-inhibited state to prevent non-specific interactions. In the free state, the nucleosome-enveloping HSA module is bound and tethered to the SRCAP core module through DMAP1 interactions with ARP6 (fig. S39A and fig. S24C). This interaction is only released upon full-engagement of the nucleosome in which ARP6 binds to the H3 tail (fig. S24D). Upon release, the HSA module envelops the nucleosome through extensive electrostatic interactions (Fig. 2H).

GAS41 interaction with the H4 N-terminal tail

In the fully engaged state of the SRCAP-nucleosome complex, GAS41 binds to the unmodified H4 tail (Fig. 2F and fig. S26C, D). This finding contrasts with earlier studies concluding that GAS41 preferentially binds to the H3 N-terminal tail (*91–94*). However, those studies used the YEATS domain or GAS41 subunit in isolation, and all but one study probed histone peptides, rather than the nucleosome substrate (*91–94*). While we cannot rule out the possibility of a transient H3 interaction in an unobserved state from the cryo-EM datasets, our findings underscore the importance of investigating histone PTM readers within the context of their full complexes and with nucleosomal substrates (*95*, *96*).

Structural basis for nucleosome engagement by SRCAP

The high-resolution (2.6-3.2 Å) map of the fully engaged SRCAP core module enabled us to accurately model the structure at the atomic level (fig. S39B). Our model of the SRCAP core module reveals the critical role of the YL1 subunit in organizing the complex (fig. S43C and Fig. 2I). The N-terminal region of YL1 contains the H2A.Z-H2B chaperone domain (*30*, *31*) which we putatively dock adjacent to the SRCAP ATPase. As observed in yeast Swc2 (*29*) (yeast homolog of YL1), human YL1 contains a dual arginine anchor motif that interacts with the H2A-H2B acidic patch on the nucleosome face opposite to the H2A-H2B dimer that is targeted for exchange. This is followed by a linker that passes over SHL-6 and connects the arginine anchor to the YL1 throttle helix. The YL1 throttle bridges the two N- and C-lobes of the SRCAP ATPase and is followed by a beta-hairpin that inserts between the OB folds of RUVBL1 and -2, constraining the SRCAP ATPase relative to the core. The carboxyl-terminus of YL1 harbors the highly conserved YL1-C domain. In yeast, the YL1-C domain of Swc2 forms a subcomplex with the fungal-specific Swc3 subunit that was shown to be critical for SWR1 chromatin binding and promoter-specific H2A.Z deposition genome wide (fig. S40A) (*29*). In the human complex, YL1-C instead folds with the insert domain of the SRCAP subunit (residues 987 to 998) (fig. S40B). While the core YL1-C fold is evolutionarily conserved, the human YL1-C:SRCAP submodule lacks the structured DNA binding domains observed in yeast Swc2:Swc3 submodule (fig. S40C). Rather, the SRCAP subunit contains a disordered 900 amino acid loop (residues 999 to 1881) that extends from the complex (fig. S40D), suggesting the evolution of a unique, intrinsically disordered region (IDR)-based mechanism for target search, as observed for the cBAF complex (*97*). Notably, the C-terminus of SRCAP subunit contains another large disordered region (residues 2357 to 3230) that contains three AT hooks shown to be important for nuclear localization and nucleosome binding of SRCAP, with mutations in this region leading to Floating-Harbor Syndrome (fig. S40D) (*98*).

The SRCAP ATPase primarily binds the nucleosomal DNA at SHL2 through its cleft between the N- and C-lobes (fig. S39C). In addition, the N-lobe is inserted between the two

gyres of nucleosomal DNA, establishing secondary contacts at SHL-6 (fig. S39C). The binding of the ATPase distorts the nucleosomal DNA, inducing a large bulge of both the tracking and guide strands of DNA (fig. S39D, E). The DNA is further distorted by the gating helix which inserts into the minor groove of the bulge (fig. S39D). Relative to previously investigated remodelers, the SRCAP gating helix is inserted deeper into the widened minor groove, disrupting DNA contacts to H3 loop 1 (R83) at SHL2.5 and inducing a kink (fig. S39E) (*39–43*). The observed DNA distortion and detachment from histones occurs on the side of the nucleosome where the DNA is unwrapped for H2A-H2B dimer eviction, indicating that the fully engaged SRCAP is poised for exchange (fig. S39B and Fig. 2C).

Structural details of the partially engaged state of SRCAP

Further classification of the cryo-EM data reveals a partially engaged state distinct from the fully engaged state. In the fully engaged state, the ZNHIT1/ARP6 module forms extensive interactions with DNA and histone H3, bringing the nucleosome closer to core SRCAP (fig. S41A-C). The N-terminal alpha-helix of ZNHIT1 (R^{17}, R^{26}, R^{30}) binds to nucleosomal DNA at the dyad (SHL0) (fig. S41B). Moreover, a positively charged loop (K^{66}KKKKTR^{72}) of ZNHIT1 dynamically interacts with the linker DNA near SHL-9 (fig. S41D). ARP6 binds the unmodified histone H3 N-terminal tail (residues 38-56), with H3 Y41 inserting into a hydrophobic pocket on the ARP6 surface (fig. S41C, E). Notably, this APR6 pocket is occupied by the N-terminal tail of DMAP1 in the preengaged state, likely functioning to position and stabilize the HSA module on the nucleosome (fig. S24C, D). In the transition from pre- to fully engaged, the large conformational shift of the HSA module and nucleosome would release the DMAP1-ARP6 interaction and position it for binding by H3.

The H3-ARP6 interaction appears to be independent of histone post-translational modifications and likely functions in anchoring to the histone octamer and sequestering the positively charged tail to prevent re-wrapping of the nucleosomal DNA (*99*). The H3-ARP6 interaction is dynamic as we were unable to fully separate H3 bound and unbound populations within the particle subset corresponding to the fully engaged state (fig. S41E, F). Collectively, these interactions highlight the function of the ZNHIT1/ARP6 module in disrupting histone-DNA contacts and firmly anchoring the nucleosome, facilitating its full engagement by the SRCAP complex.

Structural comparison of constitutive yeast Swc5 and transient human CFDP1

An integrative structural model of this region within the yeast SWR1 complex revealed a similar arrangement of interactions for the Swc5 BCNT-I interface (fig. S42B) (*29*). However, compared to the human BCNT-I interface, the yeast Swc5 BCNT-I segment is predicted to form more extensive contacts with the Swr1 and actin subunits, resulting in roughly 40% more buried surface area predicted for the Swc5 BCNT-I domain compared to that of CFDP1 (fig. S42A, B). Indeed, a mutagenesis study of yeast SWR1 showed that C-terminal truncation of the of Swr1 subunit, which also results in the loss of the actin subunit and thus would virtually eliminate the Swc5 BCNT-I interface, results in the complete loss of Swc5 from the complex, further pointing to the integral role of the BCNT-I interface in retaining Swc5 in the Swr1 complex (*47*). The other expected Swc5 binding sites on the Swr1 and RuvBL1/2 subunits are observed to be unoccupied in structures of nucleosome-bound yeast SWR1, and are thus likely transiently occupied during histone exchange as in the human holoenzyme (*29*). Thus, a the BCNT-I interface is likely the sole differentiating factor explaining why Swc5 is a constitutive component of the yeast SWR1 complex, unlike human CFDP1.

Conserved CFDP1 domains beyond BCNT-C play ancillary roles in the exchange reaction

In the poised and activated states of the SRCAP-CFDP1 holoenzyme, we observe additional CFPD1 domains extending beyond the core BCNT domain. The conserved 'guide' helix comprising residues 105-122 in the N-terminal half of CFDP1 binds to the RUVBL2 C-terminal 'DIII' domain on the outer edge of the RUVBL1/2 heterohexameric ring (Fig. 3A, B and fig. S27A-E). While SRCAP complex contains three copies of RUVBL2, we only observe concomitant occupancy of the guide helix and BCNT binding sites, suggesting these low-affinity interfaces function cooperatively to enable CFDP1 binding in specific conformational contexts of the SRCAP-nucleosome complex (Fig. 3A).

The CDFP1 N-terminus harbors a histone chaperone domain, previously shown for yeast Swc5 to preferentially bind the canonical H2A-H2B dimer (*21*). We do not observe any cryo-EM density for this AB chaperone domain in the poised state, suggesting it does not effectively compete with nucleosomal DNA for histone binding prior to unwrapping. However, we used AlphaFold2 to generate a structural prediction of the trimeric CFDP1-H2A-H2B subcomplex, which shows the acidic CFDP1 N-terminus shielding the positively charged histone surface (Fig. 3A bottom inset and fig. S28D) (*71–73*). Notably, the CFDP1 guide helix positions the AB chaperone domain near the H2A-H2B dimer to be evicted, which likely plays a role in coordinating chaperone function upon DNA unwrapping (Fig. 3A and fig. S28C).

The evicted state cryo-EM reconstruction contains additional low-resolution density near the CFDP1 BCNT-II domain and $SRCAP^{ATPase}$ that we attribute to the CFDP1 'FAGE' domain (Fig. S30E and movie S3). The predicted structure of this domain comprises a β-hairpin that packs onto a short α-helix, forming a distinct shape that allows confident fitting into the cryo-EM density (*71–73*). The turn of the β-hairpin, comprising the eponymous conserved $F^{171}AGE^{174}$ residues, binds at the junction of the CFDP1 BCNT-II helix and the $SRCAP^{ATPase}$ brace and SuppH elements (fig. S30E), while four lysine residues along one face of the FAGE α-helix are oriented to interact with the phosphate backbone of the unwrapped DNA near prior SHL3 (fig. S30E). This interface involves $SRCAP^{ATPase}$ and DNA conformations specific to the evicted state, explaining why the FAGE domain is not observed to stably interact in the preceding states (fig. S30F). We speculate that the FAGE domain functions in promoting the stability of CFDP1 BCNT binding in the DNA-unwrapped state of the complex, further ensuring hexasome stability in this vulnerable state when the histones are most prone to disassembly. Notably, as the DNA is fully unwrapped, we no longer observe density for the HSA module and associated BCNT-I domain.

Guided by the detailed structural characterization of CFDP1–SRCAP interactions, we assayed the effect of specific CFDP1 mutations on *in vitro* histone exchange activity (fig. S27H, I). Overall, mutations disrupting the C-terminal BCNT-II or BCNT-III domains were the most deleterious, with the deletion of both BCNT-II and -III domains completely abolishing histone exchange activity. By contrast, mutations within the N-terminal 232 amino acids, including deletions of the AB chaperone, guide, FAGE, or BCNT-I domains, show little to no effect on histone exchange *in vitro*, pointing to ancillary functions for these conserved domains that are apparently not critical to the core histone exchange reaction. Accordingly, a prior yeast study (*20*) has shown that residues encompassing the guide helix (Swc5 residues 80–156; corresponding to CFDP1 residues 105–122) are required for function *in vivo*.

Notably, although deletion of the human CFDP1 AB chaperone domain does not significantly reduce histone exchange activity, previous yeast Swc5 studies have reported this domain to be important (*20*, *21*). This discrepancy may reflect differences in reaction conditions, reagent quality, or mutant design: both yeast studies used Swc5Δ2–78, whereas we used CFDP1Δ1–42, a more precise truncation of the AB domain. Species-specific differences may also contribute, as yeast SWR1 assays require the Nap1 histone chaperone (in addition to Swc5), whereas human SRCAP assays do not. Notably, the two yeast studies also differ in their results—one reporting only a minor reduction in activity (*20*) and the other a substantial decrease (*21*)—despite using the same mutant. Nevertheless, the earlier study (*20*) further showed, via a yeast growth assay, that the AB domain is dispensable for *in vivo* function, consistent with our findings. We therefore propose that the AB chaperone domain is not essential for the core histone exchange mechanism but may play auxiliary roles *in vivo*.

N-terminal arginine anchors of YL1 chaperone the H2A.Z-H2B dimer

Notably, in our preinserted and fully inserted states, we observe additional regions of YL1 chaperoning the H2A.Z-H2B dimer, revealing interactions beyond those captured in prior crystallographic studies (fig. S43A, B) (*30*, *31*). The distinction between the cryo-EM structure and the human YL1-H2A.Z-H2B crystal structure likely stems from crystal-packing-induced dimerization of YL1 residues 16-24 (fig. S43B) (*31*), resulting in R10 of YL1 interacting with a distinct symmetry-related YL1-H2A.Z-H2B complex. In the cryo-EM structure, we observe similar interactions where YL1 residues 25-68 bind to the H2A.Z αC-helix and the DNA binding surface of H2A.Z-H2B dimer (fig. S43A). We further resolve conserved N-terminal YL1 residues 1-17 which interact with loop 3 of H2A.Z and the H2A.Z-H2B acidic patch where YL1 R7 serves as a canonical arginine anchor and R10 as a type 2 variant (fig. S43A). Following the arginine anchors, YL1 S2 forms a hydrogen bond with H2B D48. Collectively, YL1 contains four total arginine anchors, with two serving to chaperone H2A.Z-H2B and the other two anchoring to the nucleosome H2A-H2B acidic patch (fig. S43C).

Mechanism of partial DNA unwrapping by SRCAP apoenzyme

During the preparation of this manuscript, another study reporting multiple cryo-EM structures of the SRCAP(apoenzyme)-nucleosome complex was published (*17*). These authors recombinantly overexpressed and purified the SRCAP complex from human cells, assembled SRCAP-nucleosome complexes, and subjected the sample to gradient fixation (glycerol gradient with glutaraldehyde crosslinking). They report three structures of the SRCAP-nucleosome complex that the authors define on the basis of the nucleotide free and bound states of the SRCAP ATPase (apo, ADP-BeF3, and ADP). The apo and ADP states closely resemble our preengaged and fully engaged states, respectively, despite our sample containing ATPγS; the ADP-BeF3 state is conformationally intermediate between our preengaged and fully engaged states. Notably, their sample preparation method involved pre-incubation with ATP prior to addition of nucleotide analogs (ADP-BeF3, and ADP) then extensive treatment (fixation and purification) of their SRCAP-nucleosome complexes for cryo-EM.

Importantly, our interpretation of the distinct conformational states as part of a stepwise pathway towards nucleosome engagement independent of ATP hydrolysis, is conceptually distinct from the conclusion of Yu et al. (*17*) that ATP hydrolysis is required for full engagement of the nucleosome by the SRCAP complex and the unwrapping of nucleosomal DNA by the

ARP6-ZNHIT1 subunits. Indeed, we were able to observe this fully engaged, DNA unwrapped state in the context of SRCAP ATPase bound with unhydrolyzed ATPγS or AMP-PNP. Therefore, we conclude that the key requirement for ATP hydrolysis serves a later stage in the histone exchange reaction after nucleosome engagement and partial DNA unwrapping. Importantly, our results are consistent with previous cryo-EM structures of yeast SWR1 complex assembled with nonhydrolyzable ATP analogs as well as single molecule studies revealing partial DNA unwrapping with nonhydrolyzable ATP analogs (*28*, *29*, *33*).

Additionally, the Yu et al. study (*17*) suggests ZNHIT1 directly evicts the H2A-H2B dimer based on its interaction with the H2A-H2B acidic patch. Our findings reveal a different mechanism. We demonstrate that the CFDP1-activated $SRCAP^{ATPase}$ unwraps the nucleosomal DNA and that the restructuring of the histone H3 αN-helix by ARP6 facilitates eviction of the exposed dimer. The previous study observed ZNHIT1 interaction with H2A in the preengaged state prior to nucleosome engagement, suggesting the interaction likely functions in SRCAP nucleosome engagement rather than in the enzymatic histone exchange process. The authors also suggest that the HSA module functions to restrain propagation of DNA distortion and prevent net DNA translocation. In contrast, our results indicate it plays a direct role in unwrapping the nucleosomal DNA in coordination with $SRCAP^{ATPase}$.

<u>Requirements of histone exchange</u>

An early study using SRCAP apoenzyme immuno-purified from HeLa cells demonstrated histone exchange activity, using a western blot-based assay to detect H2A.Z incorporation (*13*). However, our study using endogenous SRCAP apoenzyme from K562 cells, along with more recent work (*17*) using recombinant SRCAP apoenzyme from HEK293 cells, showed no histone exchange activity on nucleosomes. We attribute this discrepancy to the limitations of western blotting detection used in the early work which does not differentiate between nucleosome-incorporated H2A.Z-H2B dimer and non-specific linker DNA associated dimer. In our EMSA-based histone exchange assay (*26*, *27*), we resolve this issue by using excess Lambda DNA which absorbs non-nucleosomal dimers and thus allows for specific detection of H2A.Z-H2B incorporated as part of the histone octamer. Furthermore, the SRCAP apoenzyme purified in the earlier study (*13*) likely lacked functionality due to the absence of ZNHIT1 and YL1 subunits, which play critical roles in nucleosome engagement and H2A.Z-H2B incorporation (this study and *17*). Hence, we conclude that CFDP1 is essential for *in vitro* histone exchange and that western blotting is an inadequate method for assessing histone exchange activity.

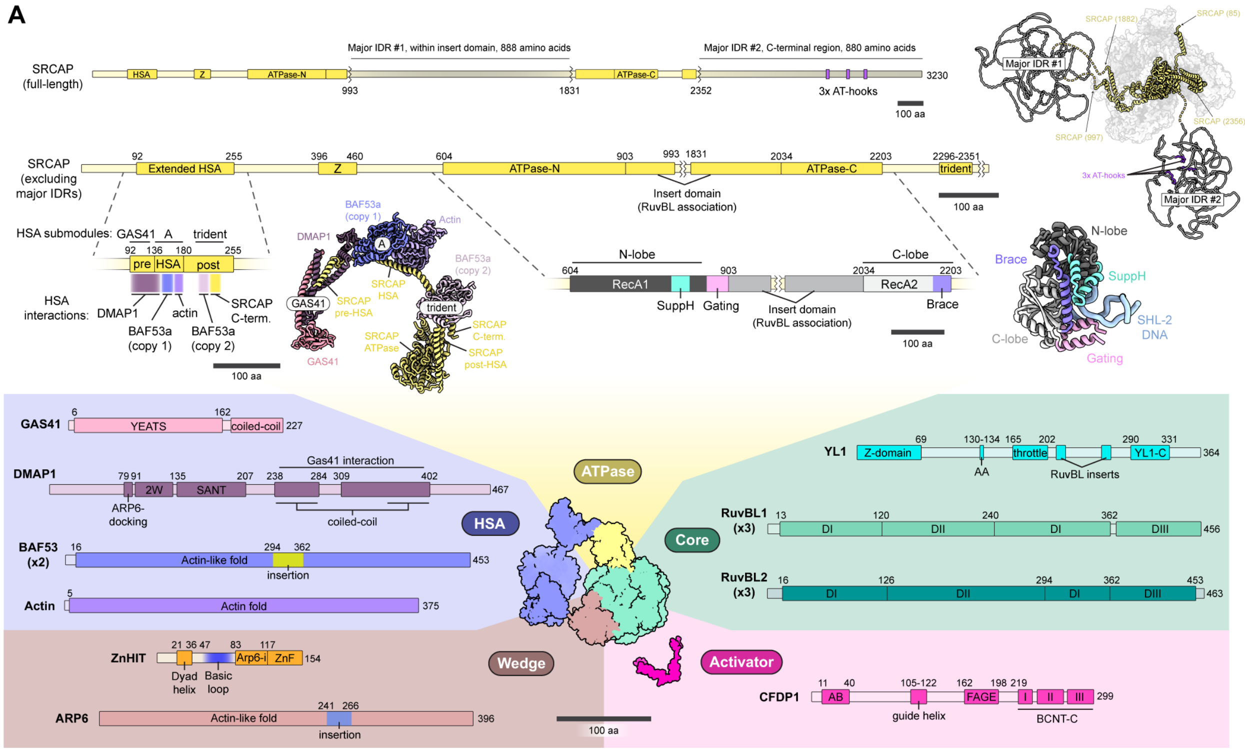

Fig. S1. Domain architecture of the human SRCAP complex.

(A) Domain diagram of human SRCAP complex subunits. Each subunit is grouped according to the functional modules defined in Fig. 1A. Relevant structural models are shown where appropriate.

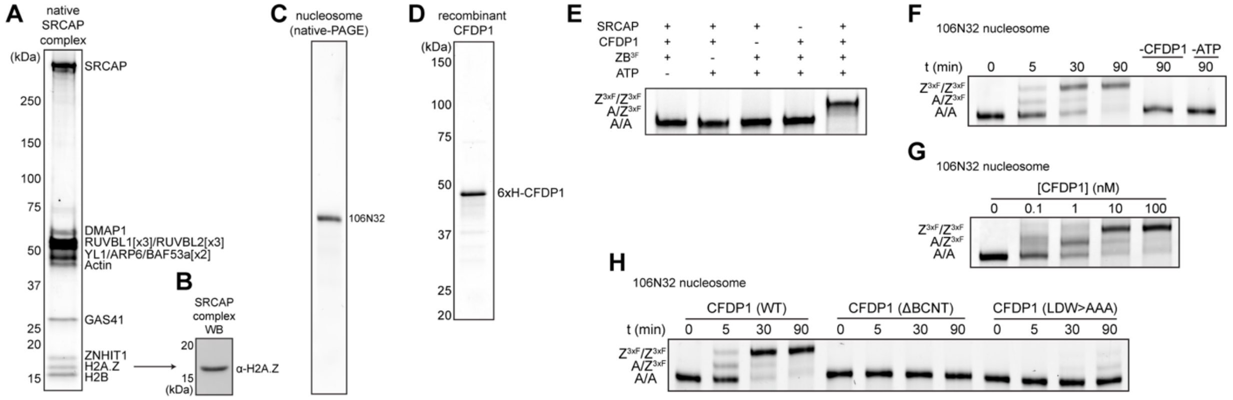

Fig. S2. Biochemical characterization of SRCAP-CFDP1 mediated histone H2A.Z exchange.

(A) SDS-PAGE of native SRCAP complex purified from K562 cells (see methods). (B) Western blot analysis of native SRCAP complex for histone H2A.Z. (C) Native PAGE of 106N32 nucleosome. (D) SDS-PAGE of bacterially expressed full-length human CFDP1. (E) Histone exchange assay (90 minute time point) with all components required for activity (lane 1) and without SRCAP (lane 2), CFDP1 (lane 3), H2A.Z-H2B (lane 4), or ATP (lane 5). (F) Histone exchange assay results at different time points (0, 5, 30, 90 minutes) with all components (lane 1-4), without CFDP1 (lane 5, 90 minutes), and without ATP (lane 6, 90 minutes) as in Fig. 1D but with unlabeled 106N32 nucleosomes used for cryo-EM. (G) Histone exchange assay with different concentrations of CFDP1 (0, 0.1, 1, 10, and 100 nM) as in Fig. 1E but with unlabeled 106N32 nucleosomes. (H) Histone exchange assay at different time points (0, 5, 30, 90 minutes) with WT, ΔBCNT-C, and LDW alanine substitution mutant ($L^{247}D^{248}W^{249}$ -> $A^{247}A^{248}A^{249}$) for CFDP1 as in Fig. 3C but with unlabeled 106N32 nucleosomes.

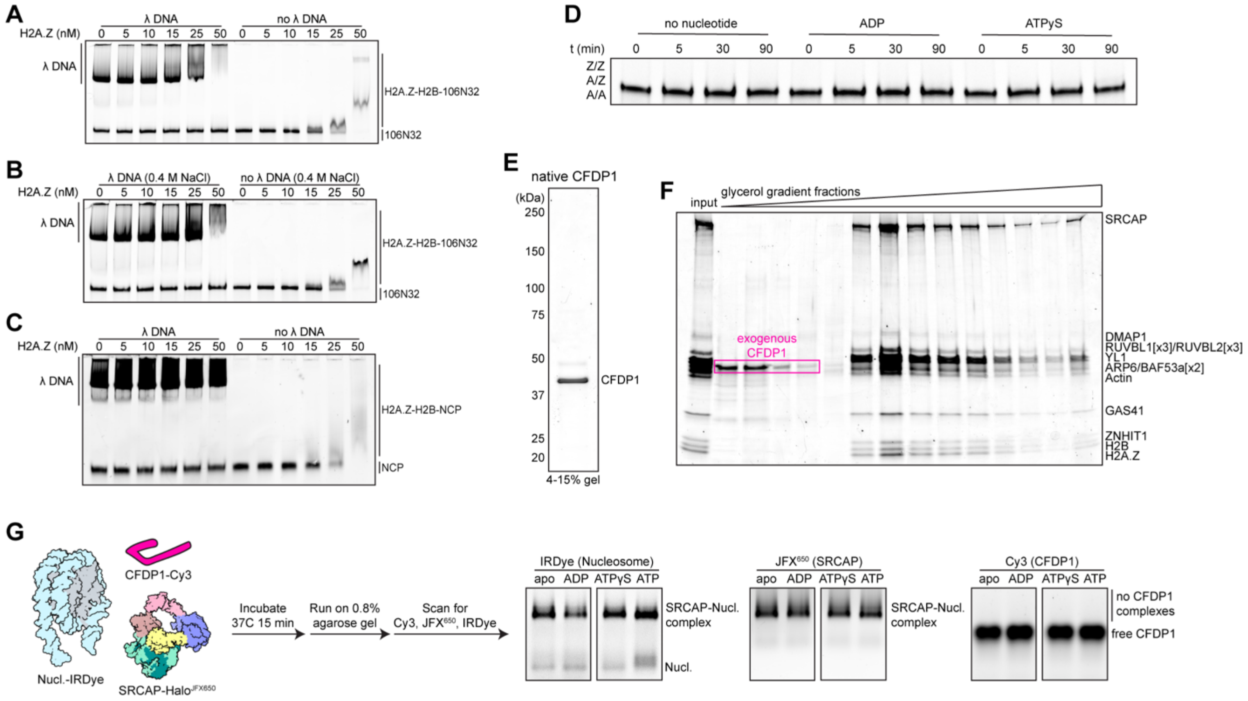

Fig. S3. Biochemical characterization of SRCAP-CFDP1 mediated histone H2A.Z exchange (continued).

(A) 106N32 nucleosomes incubated with an increasing amount of H2A.Z-H2B dimer (0 to 50 nM) with (lane 1-6) or without (lane 7-12) lambda DNA in histone exchange buffer. H2A.Z-H2B dimer non-specifically associates with nucleosomes in the absence of lambda DNA. (B) Same as panel (A), except that histone exchange buffer was supplemented with NaCl (final 0.4 M NaCl). Non-specific dimer association (without lambda DNA) occurs even at high salt concentrations. (C) Same as panel (A), except that nucleosome core particles (NCPs, no linker DNA) were used. H2A.Z-H2B dimer non-specifically associates with NCPs in the absence of lambda DNA. (D) Histone exchange assay without nucleotide (apo, lane 1-4), with ADP (lane 5-8), and with ATPγS (lane 9-12). (E) SDS-PAGE of recombinant full-length CFDP1 purified from human Expi293 cells. (F) SDS-PAGE of input (lane 1) and glycerol gradient fractions (15-45% glycerol, lanes 2-15) of native SRCAP complex supplemented with excess CFDP1. Exogenously added CFPD1 is boxed in pink. While CFDP1 did not co-migrate stoichiometrically, there was a minor overlap in glycerol gradient fractions. (G) Schematic and results of 3-color EMSA with labeled nucleosomes, SRCAP, CFDP1, and different nucleotide conditions (apo - no nucleotide, ADP, ATPγS, ATP). The same gel was scanned for nucleosomes (IRDye), SRCAP (JFX^{650} dye), and CFPD1 (Cy3).

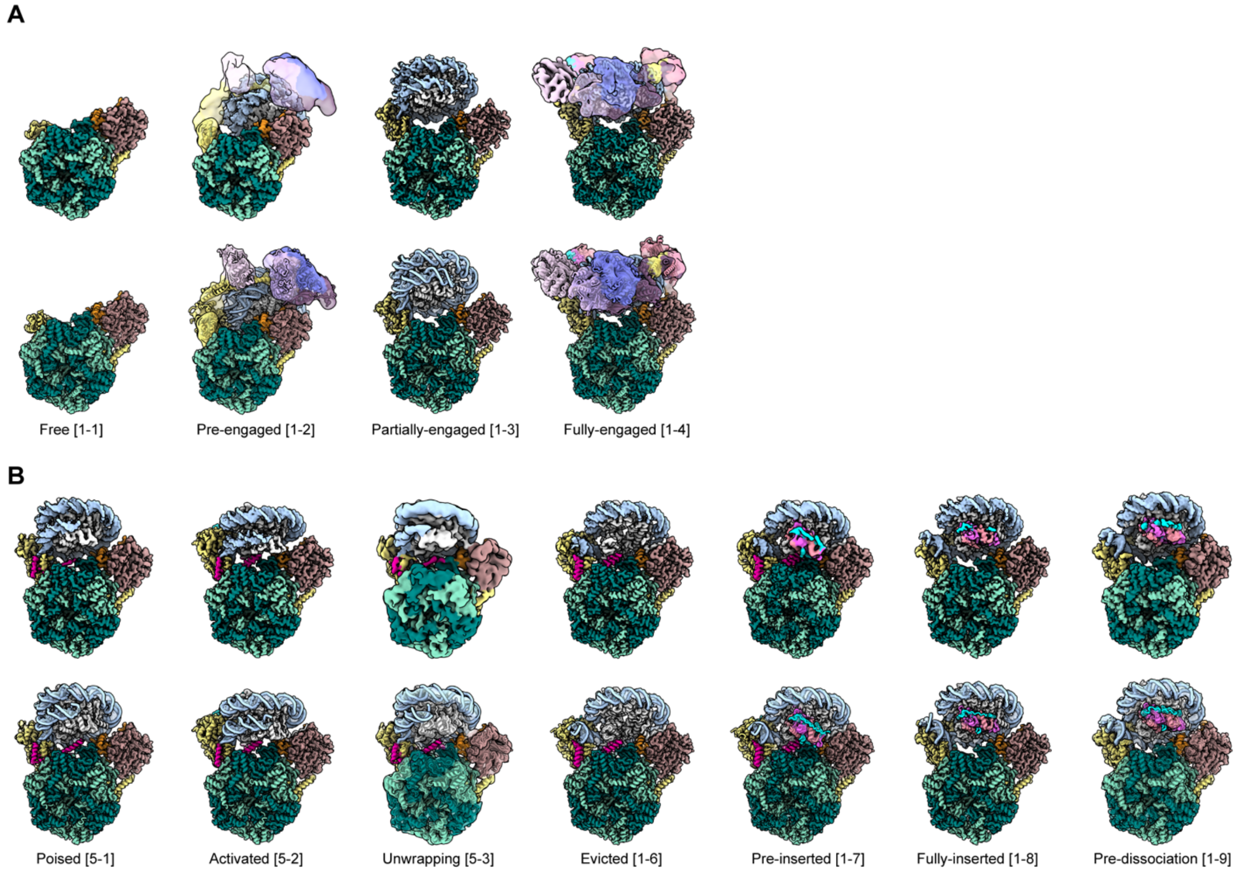

Fig. S4. Gallery of all cryo-EM states observed.
(A) Cryo-EM map (top row, lower res regions shown transparent) and cryo-EM model fit into the map (bottom row, cryo-EM map shown transparent) of free, preengaged, partially engaged, fully engaged states. (B) Cryo-EM map (top row) and cryo-EM model fit into the map (bottom row, cryo-EM map shown transparent) of poised, activated, unwrapping, evicted, preinserted, fully inserted, and predissociation states.

A states observed in each dataset

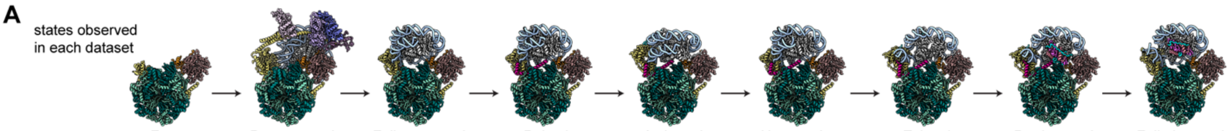

	Free	Pre-engaged	Fully-engaged	Poised	Activated	Unwrapping	Evicted	Pre-inserted	Fully-inserted
Dataset 1	+	+	+	-	+	-	+	+	+
Dataset 2	-	+	-	-	-	-	-	-	-
Dataset 3	-	-	+	-	-	-	-	-	-
Dataset 4	-	-	+	-	-	-	-	-	-
Dataset 5	-	-	-	+	+	+	-	-	-
Dataset 6	-	-	+	-	-	-	-	-	-
Dataset 7	-	-	-	+	+	-	-	-	-
Dataset 8	-	-	-	+	-	-	-	-	-
Dataset 9	-	-	-	-	-	-	+	-	-
Dataset 10	-	-	-	-	-	-	+	-	-
Dataset 11	-	-	-	-	-	-	+	-	-
Dataset 12	-	-	-	-	-	-	+	-	+

Fig. S5. Cryo-EM states observed in each dataset.
(A) Major cryo-EM states observed in each dataset (1-12).

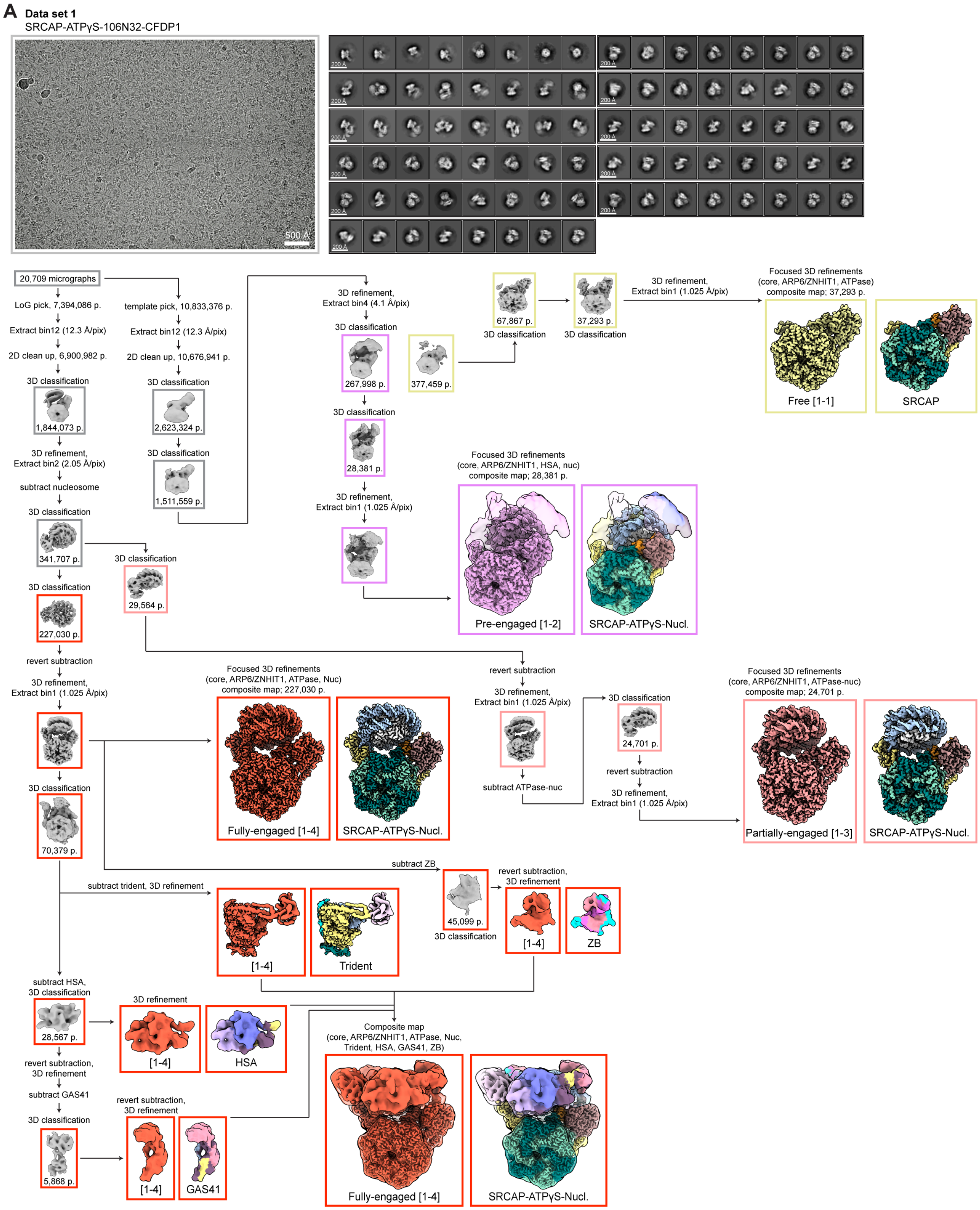

Fig. S6. Cryo-EM processing of data set 1.

(A) Cryo-EM processing of data set 1 (SRCAP-ATPγS-CFDP1-Nucleosome). The cryo-EM micrograph, 2D class averages, and processing trees for the free, preengaged, partially engaged, and fully engaged states are shown.

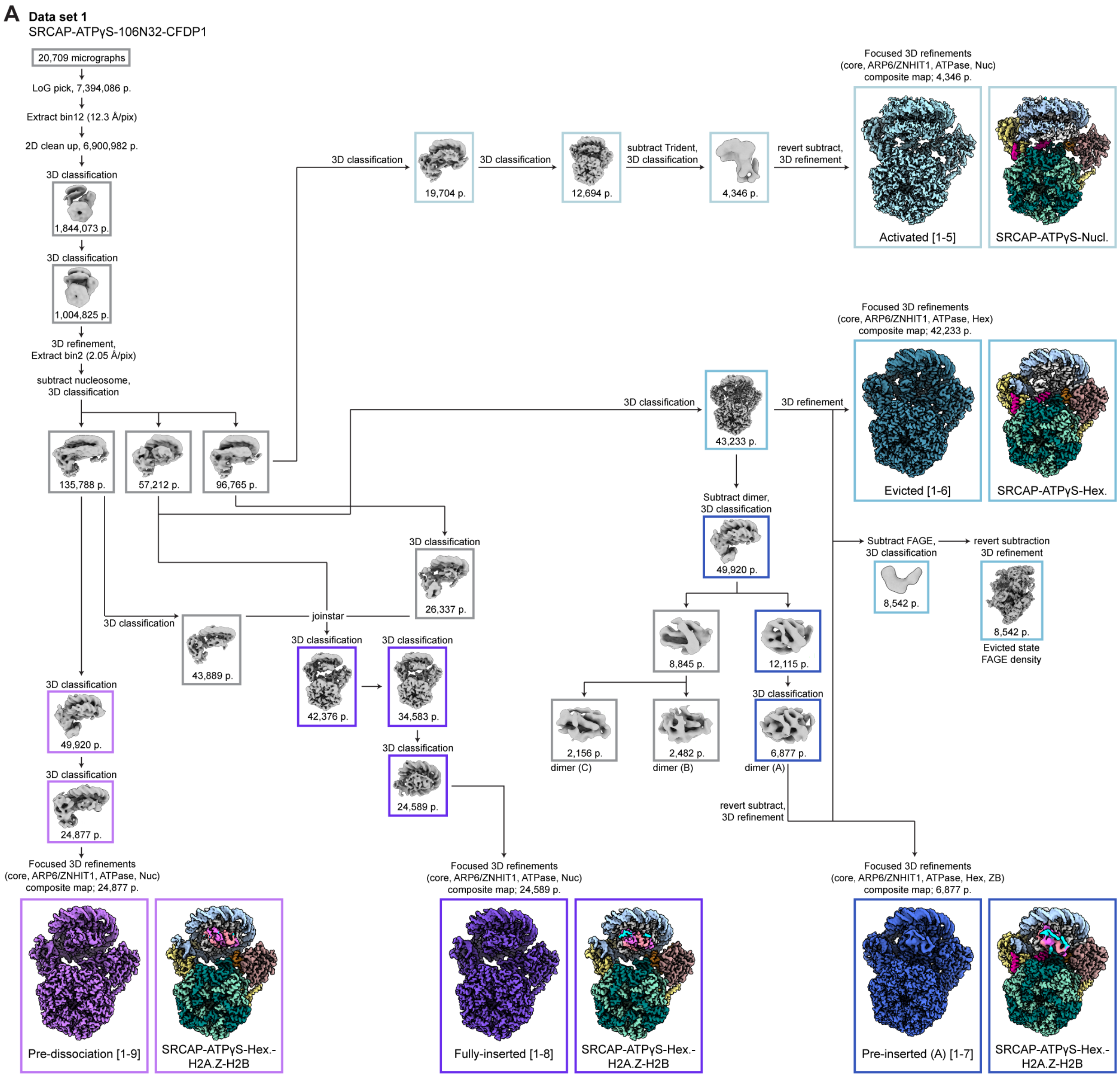

Fig. S7. Cryo-EM processing of data set 1 (continued).
(A) Cryo-EM processing of data set 1 (SRCAP-ATPγS-CFDP1-Nucleosome). Processing trees for the activated, evicted, preinserted, fully inserted, and predissociation states are shown.

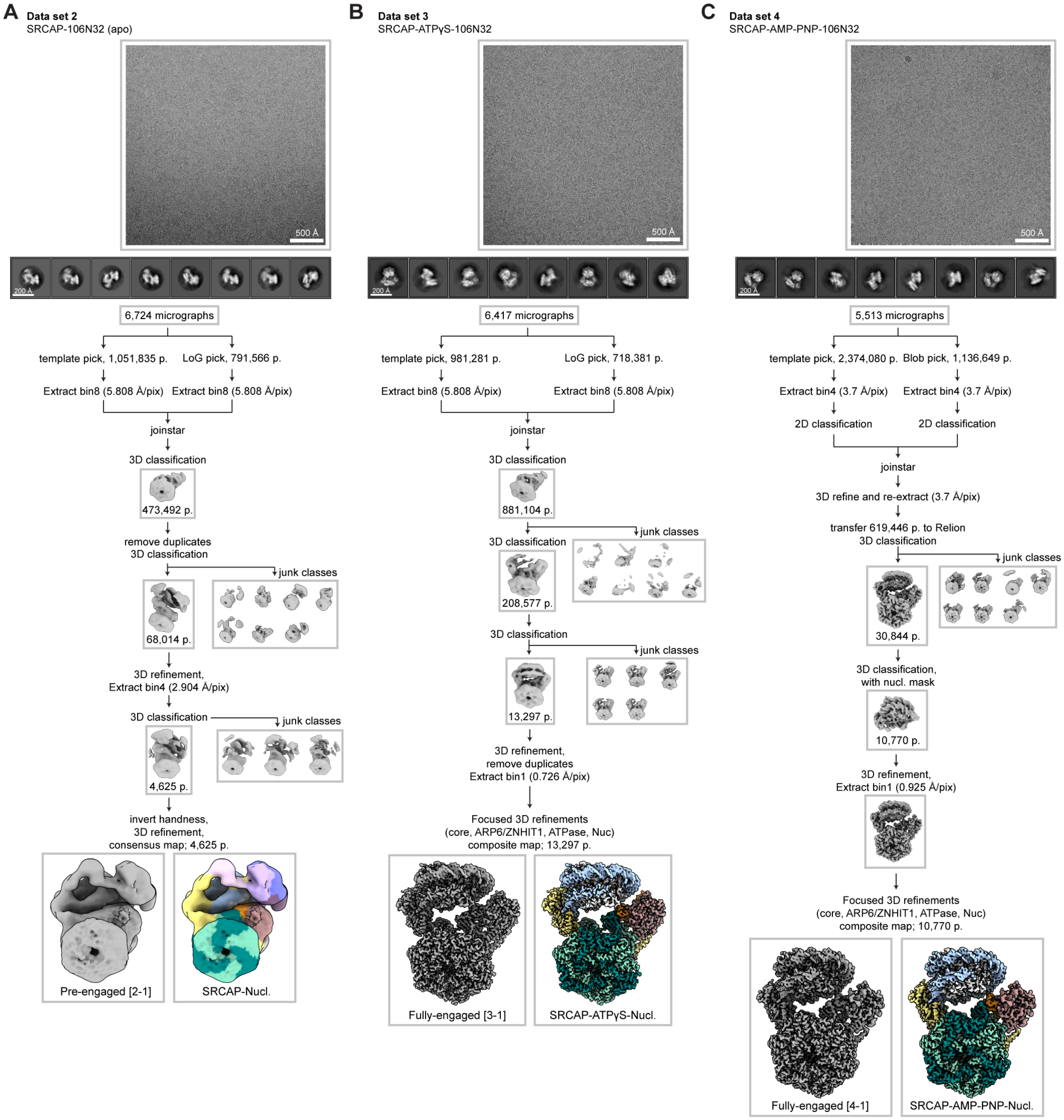

Fig. S8. Cryo-EM processing of data sets 2-4.
(A) Cryo-EM processing of data set 2 (SRCAP-Nucleosome). The cryo-EM micrograph, 2D class averages, and processing tree for the preengaged state are shown. (B) Cryo-EM processing of data set 3 (SRCAP-ATPγS-Nucleosome). The cryo-EM micrograph, 2D class averages, and processing tree for the fully engaged state are shown. (C) Cryo-EM processing of data set 3 (SRCAP-AMP-PNP-Nucleosome). The cryo-EM micrograph, 2D class averages, and processing tree for the fully engaged state are shown.

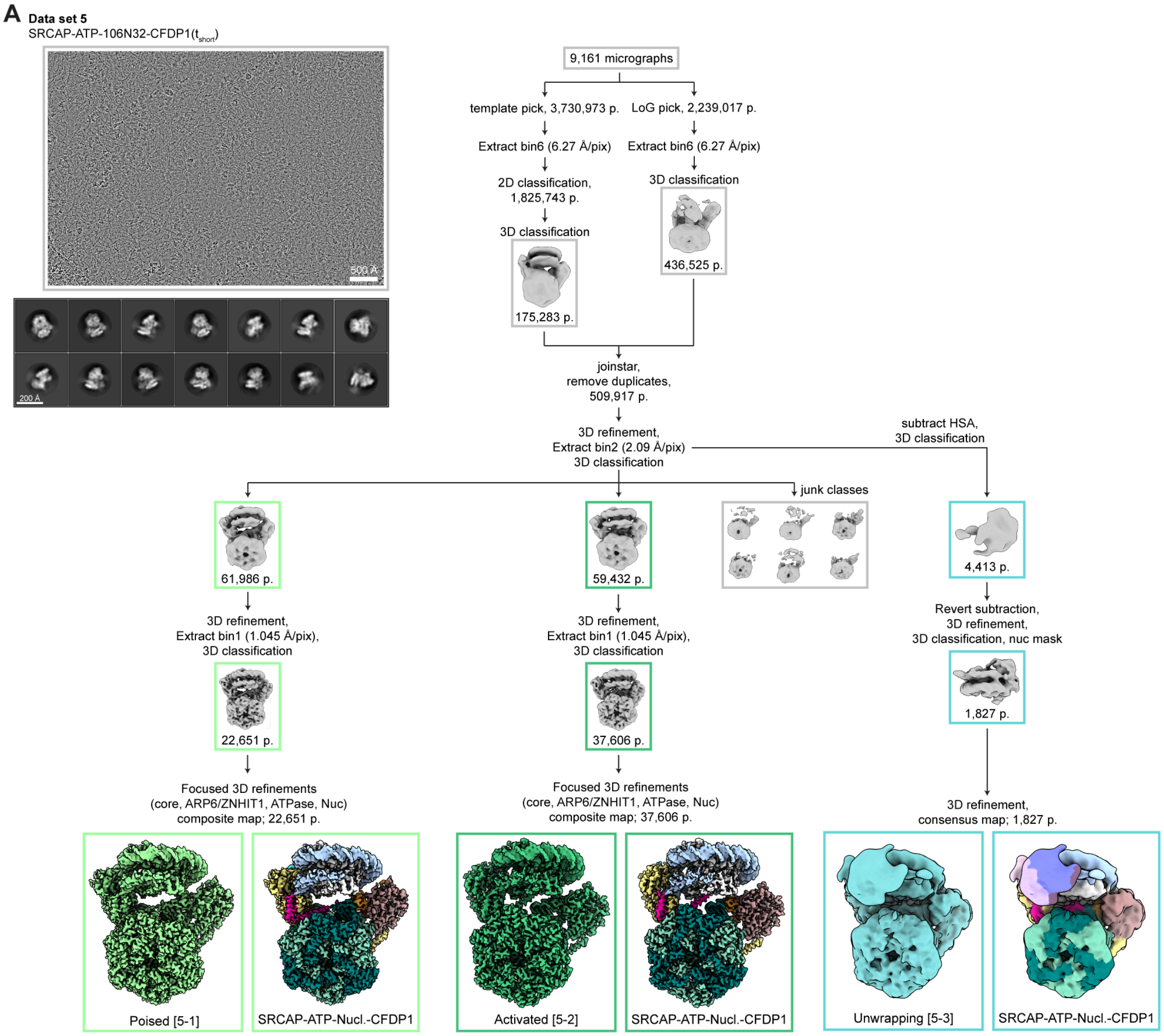

Fig. S9. Cryo-EM processing of data set 5.
(A) Cryo-EM processing of data set 5 (SRCAP-ATP-CFDP1-Nucleosome). The cryo-EM micrograph, 2D class averages, and processing trees for the poised, activated, and unwrapping states are shown.

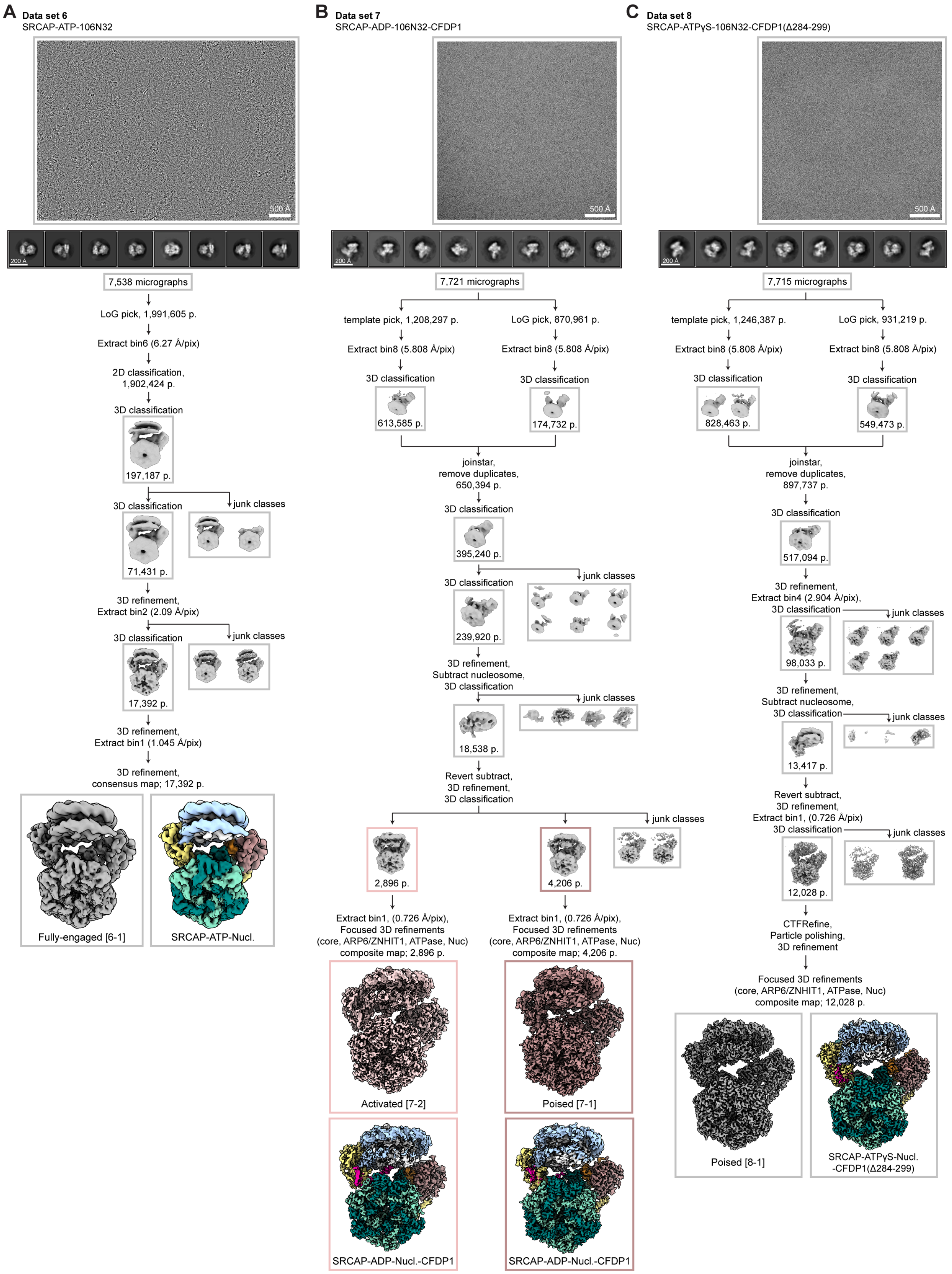

Fig. S10. Cryo-EM processing of data sets 6-8.

(A) Cryo-EM processing of data set 6 (SRCAP-ATP-Nucleosome). The cryo-EM micrograph, 2D class averages, and processing tree for the fully engaged state are shown. (B) Cryo-EM processing of data set 7 (SRCAP-ADP-CFDP1-Nucleosome). The cryo-EM micrograph, 2D class averages, and processing trees for the poised and activated states are shown. (C) Cryo-EM processing of data set 8 (SRCAP-ATPγS-CFDP1(Δ284-299)-Nucleosome). The cryo-EM micrograph, 2D class averages, and processing tree for the poised state are shown.

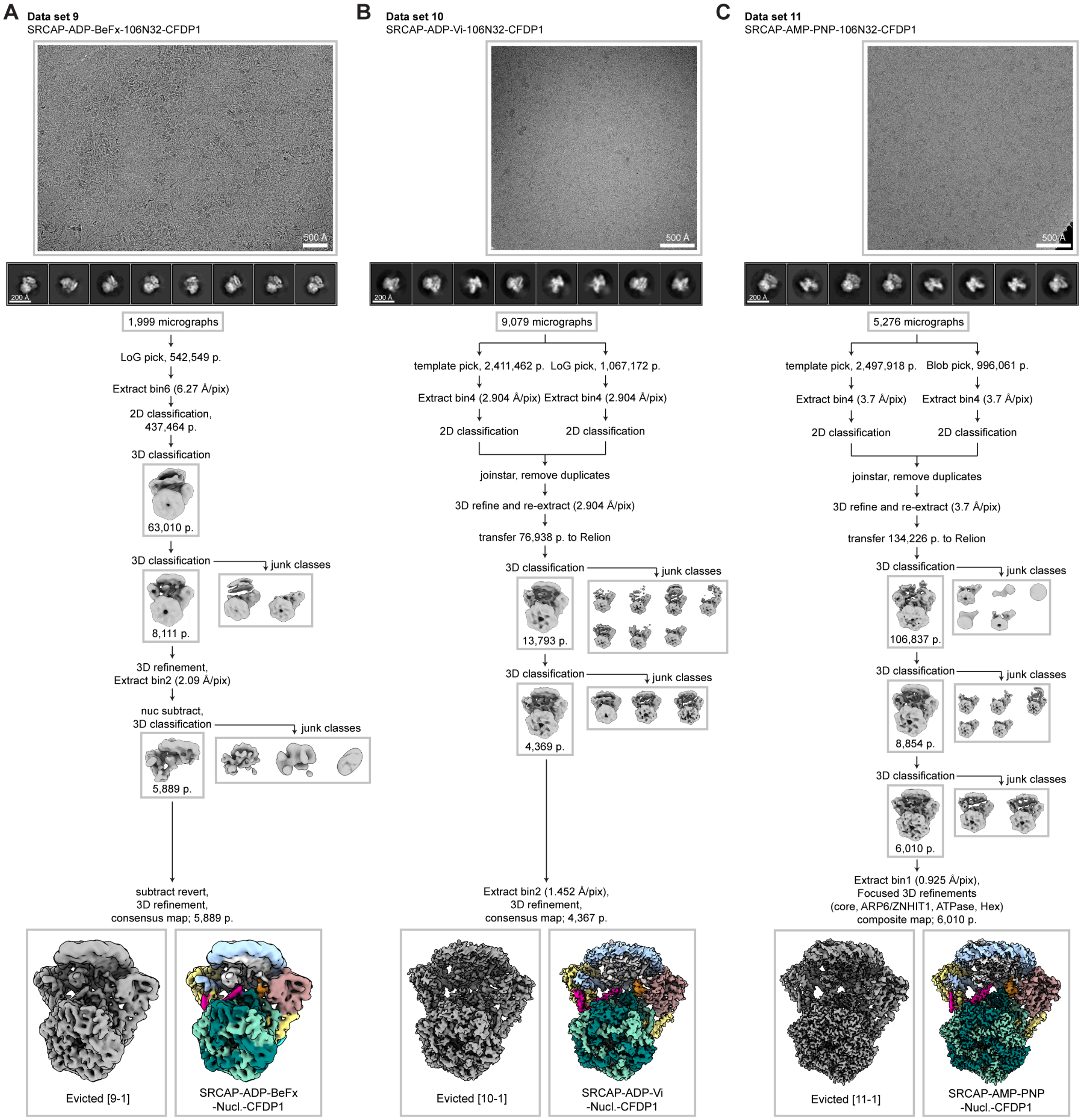

Fig. S11. Cryo-EM processing of data sets 9-11.
(A) Cryo-EM processing of data set 9 (SRCAP-ADP-BeF$_x$-Nucleosome). The cryo-EM micrograph, 2D class averages, and processing tree for the evicted state are shown. (B) Cryo-EM processing of data set 10 (SRCAP-ADP-V$_i$-CFDP1-Nucleosome). The cryo-EM micrograph, 2D class averages, and processing tree for the evicted state are shown. (C) Cryo-EM processing of data set 11 (SRCAP-AMP-PNP-CFDP1-Nucleosome). The cryo-EM micrograph, 2D class averages, and processing tree for the evicted state are shown.

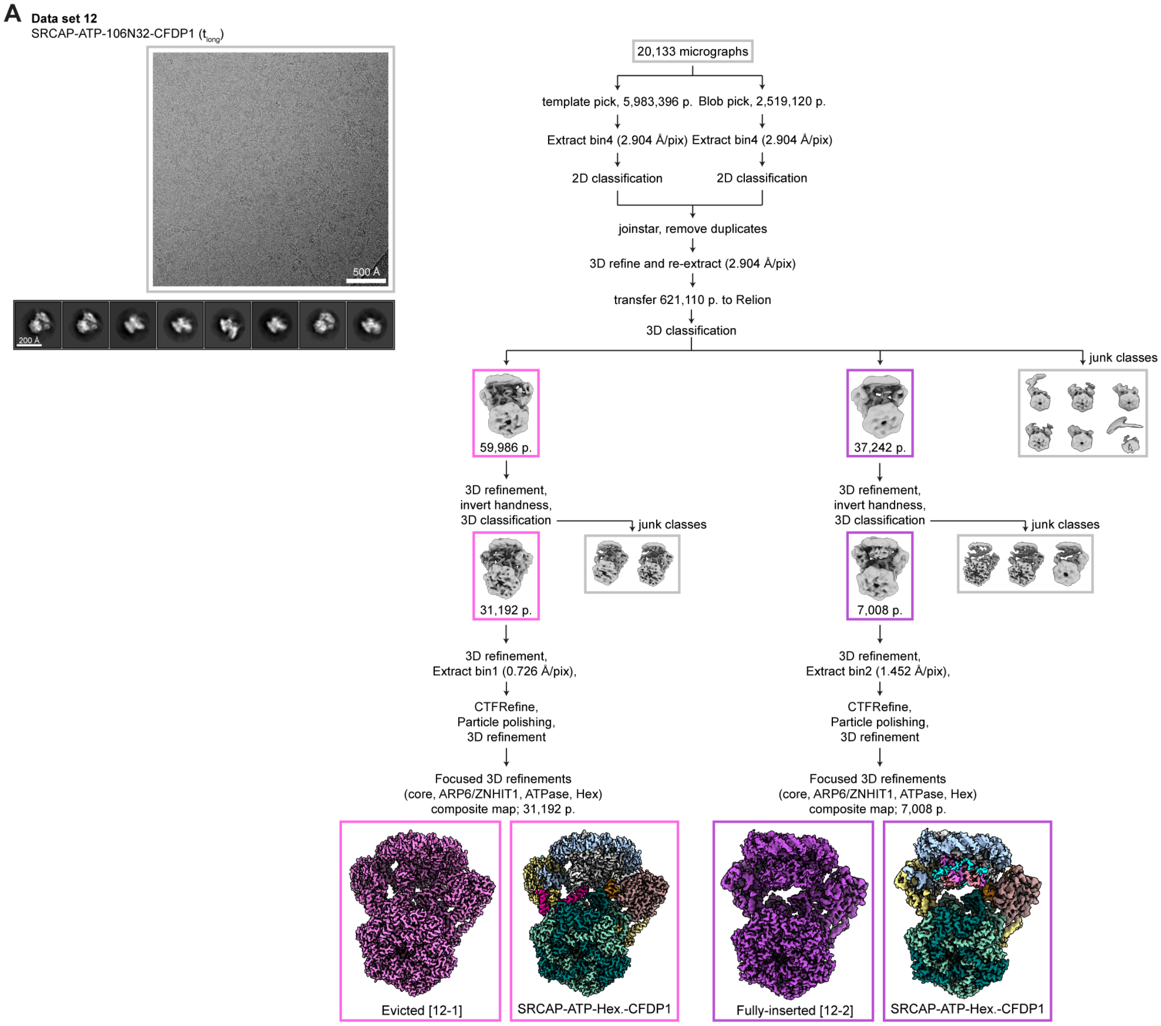

Fig. S12. Cryo-EM processing of data set 12.

(A) Cryo-EM processing of data set 12 (SRCAP-ATP-CFDP1-Nucleosome). The cryo-EM micrograph, 2D class averages, and processing trees for the evicted and fully inserted states are shown.

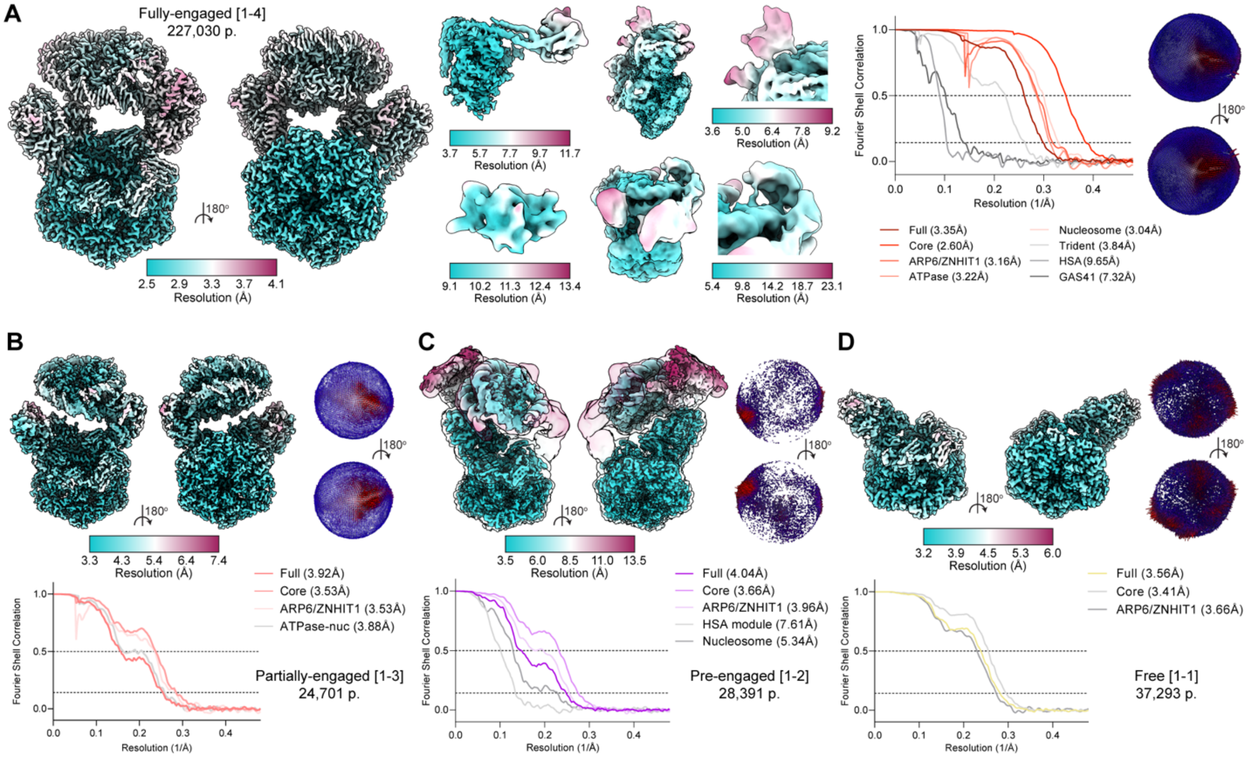

Fig. S13. Quality and resolution of cryo-EM maps for the fully engaged, partially engaged, preengaged and free states.

(A-D) Local resolution colored composite cryo-EM maps, orientation distribution plots, and mask corrected gold-standard Fourier shell correlation (FSC) plots for the indicated data sets and states.

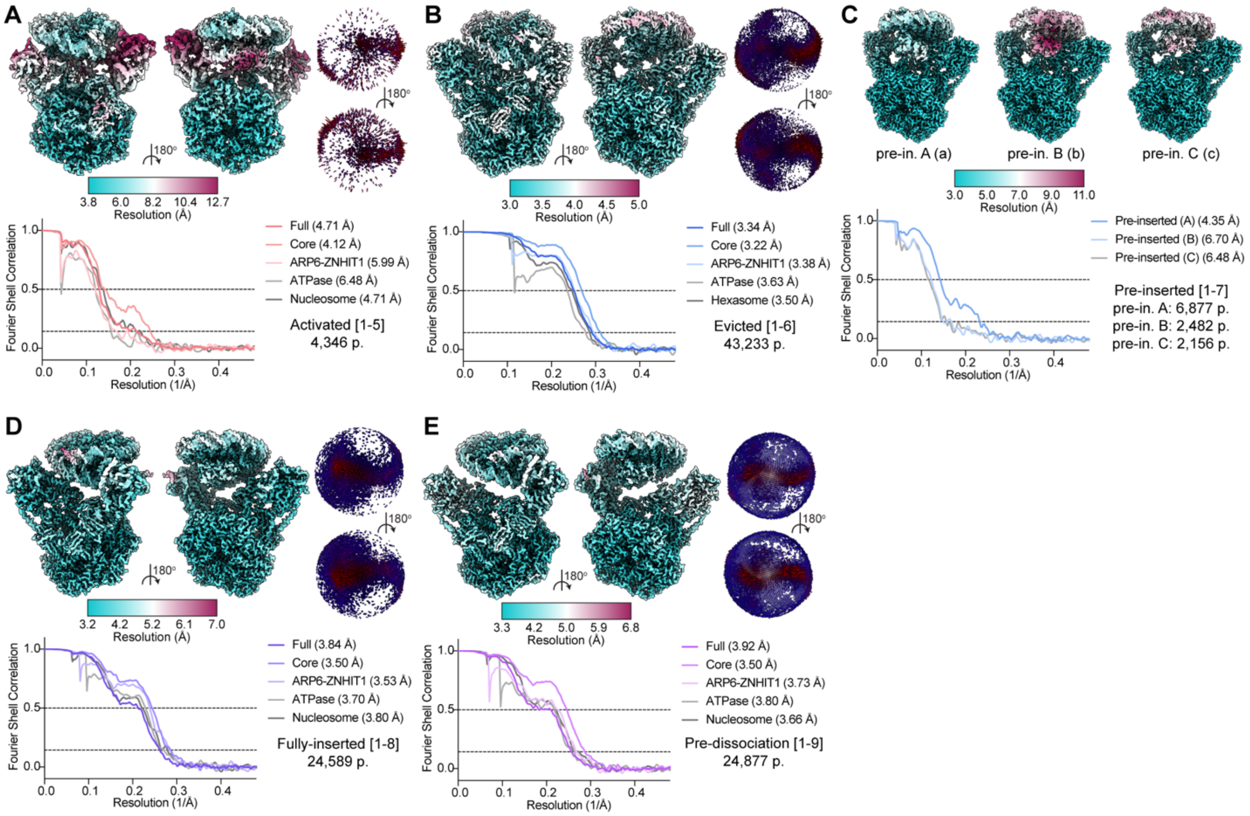

Fig. S14. Quality and resolution of cryo-EM maps for the activated, evicted, preinserted, fully inserted, and predissociation states.

(A-F) Local resolution colored composite cryo-EM maps, orientation distribution plots, and mask corrected gold-standard Fourier shell correlation (FSC) plots for the indicated data sets and states.

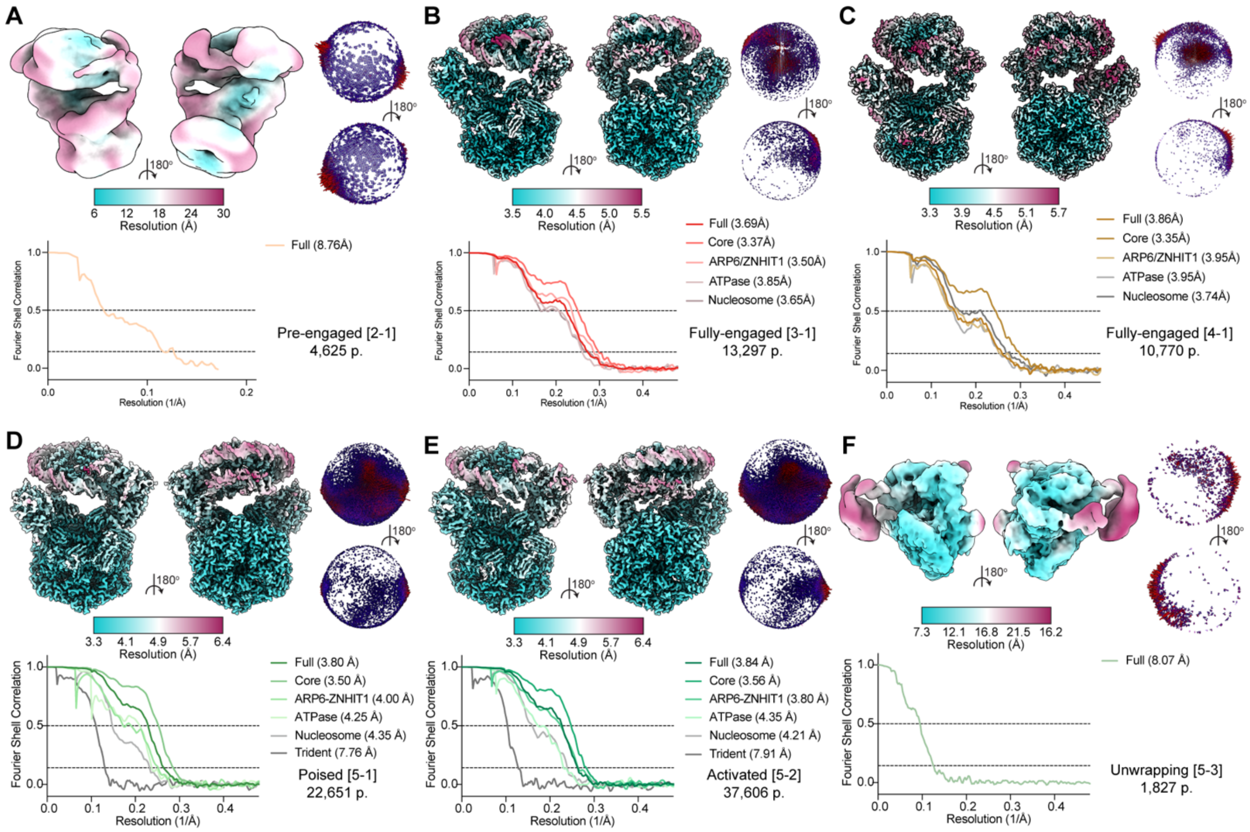

Fig. S15. Quality and resolution of cryo-EM maps for the preengaged, fully engaged, poised, activated, and unwrapping states.

(A-F) Local resolution colored composite cryo-EM maps, orientation distribution plots, and mask corrected gold-standard Fourier shell correlation (FSC) plots for the indicated data sets and states.

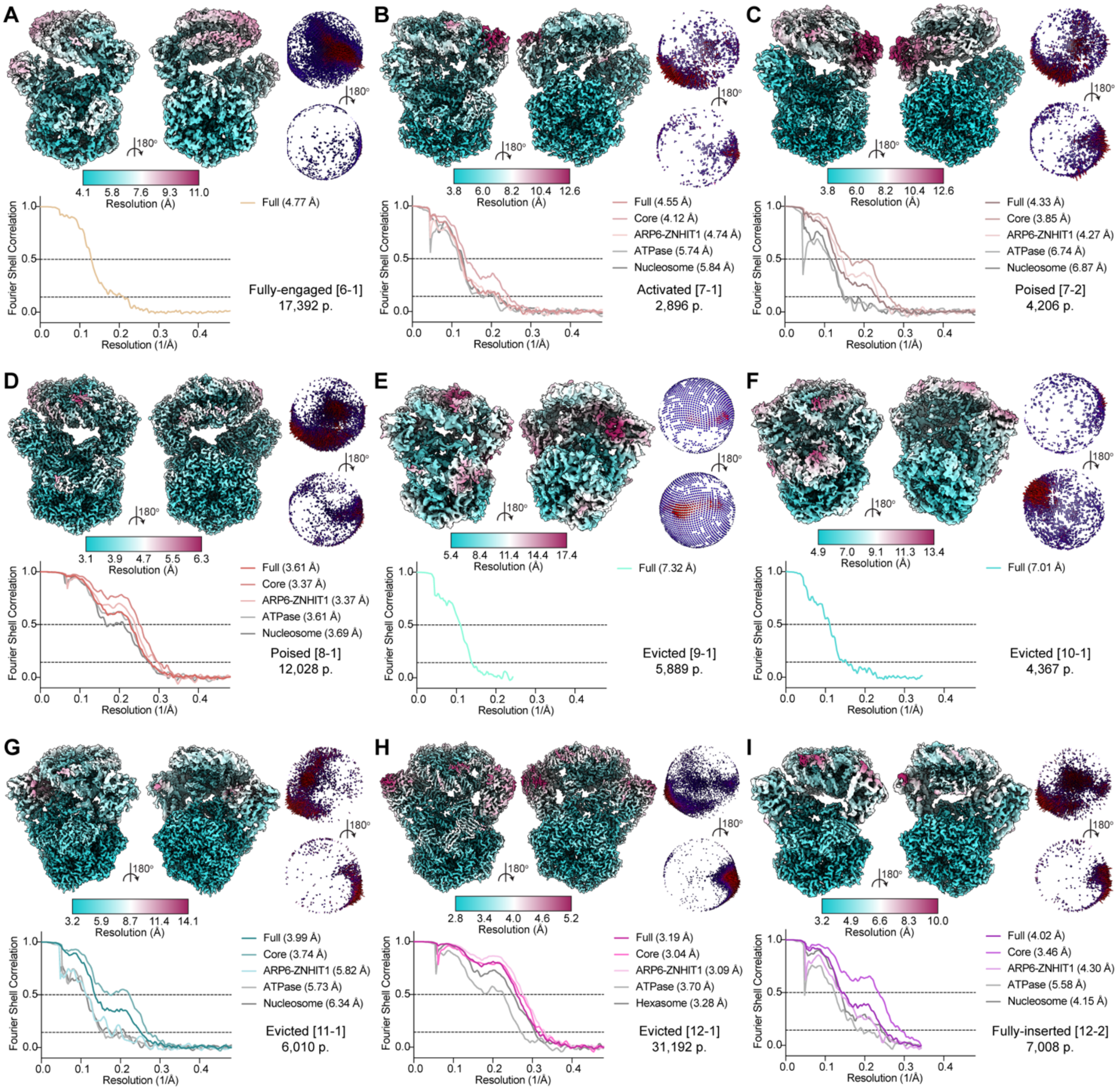

Fig. S16. Quality and resolution of cryo-EM maps for the fully engaged, activated, poised, evicted, and fully inserted states.

(A-I) Local resolution colored composite cryo-EM maps, orientation distribution plots, and mask corrected gold-standard Fourier shell correlation (FSC) plots for the indicated data sets and states.

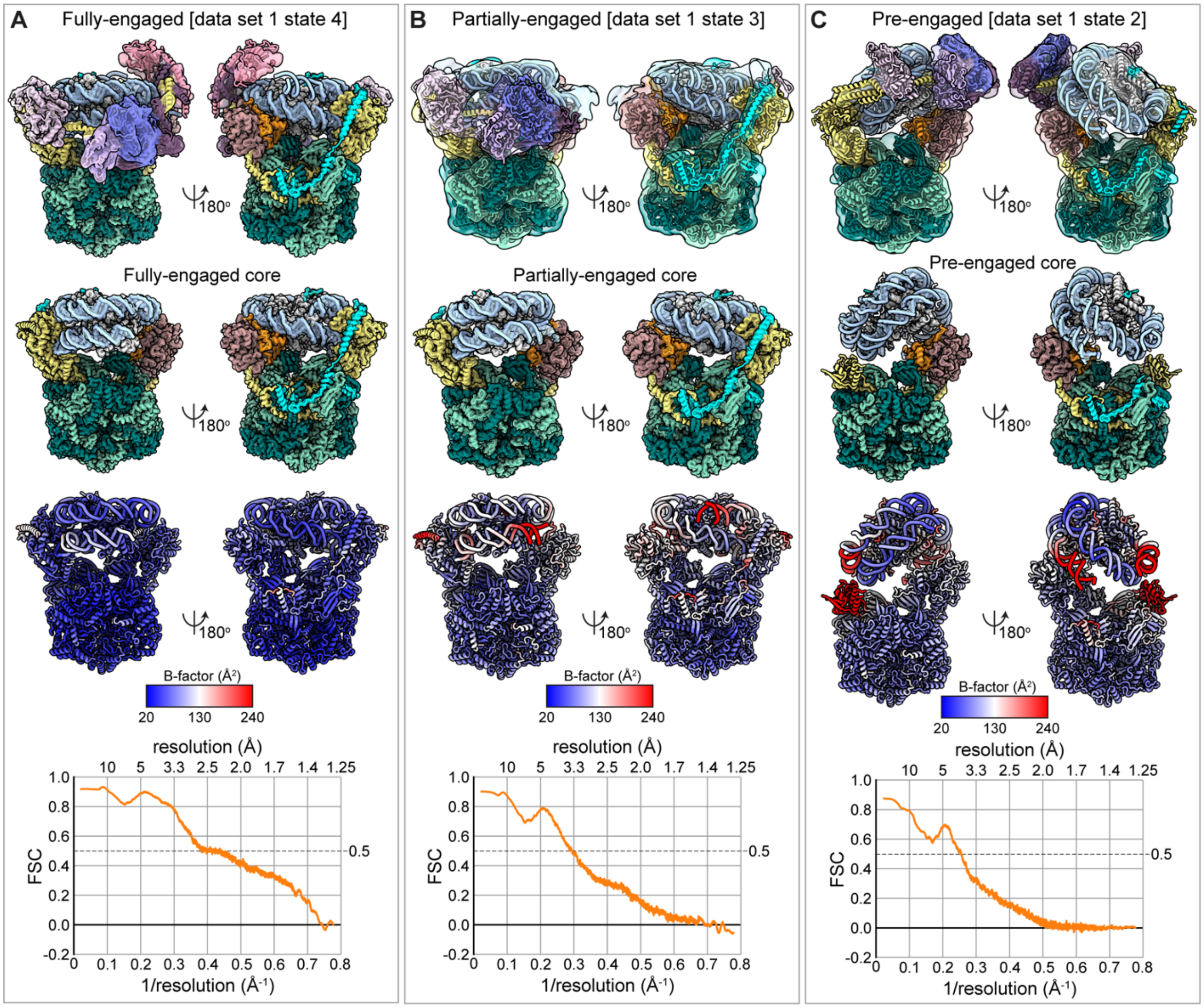

Fig. S17. Model building of the fully engaged, partially engaged, and preengaged cryo-EM structures.

(A-C) Overall fit of SRCAP model (A, Fully engaged; B, Partially engaged; C, Preengaged) into the lower-resolution full cryo-EM map (top). Overall fit of refined SRCAP model (A, Fully engaged; B, Partially engaged; C, Preengaged) into the high-resolution composite cryo-EM map (middle). Refined model colored by PHENIX B-factor estimation (bottom). Map vs. model Fourier shell correlation (FSC) shown below.

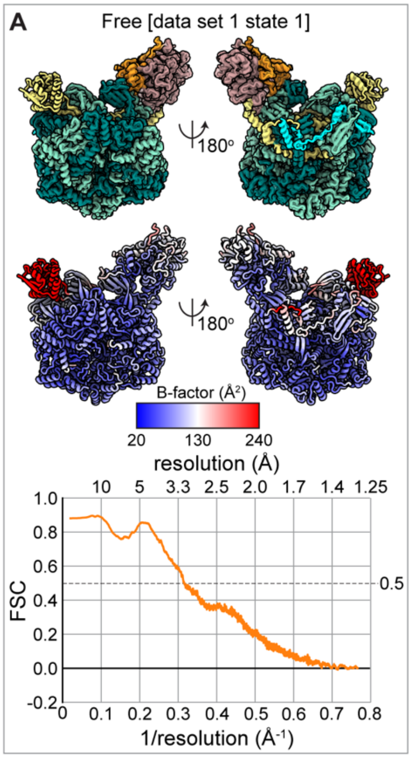

Fig. S18. Model building of the free cryo-EM structure.
(A) Overall fit of refined SRCAP model (A, Free) into the high-resolution composite cryo-EM map (top). Refined model colored by PHENIX B-factor estimation (middle). Map vs. model Fourier shell correlation (FSC) shown below.

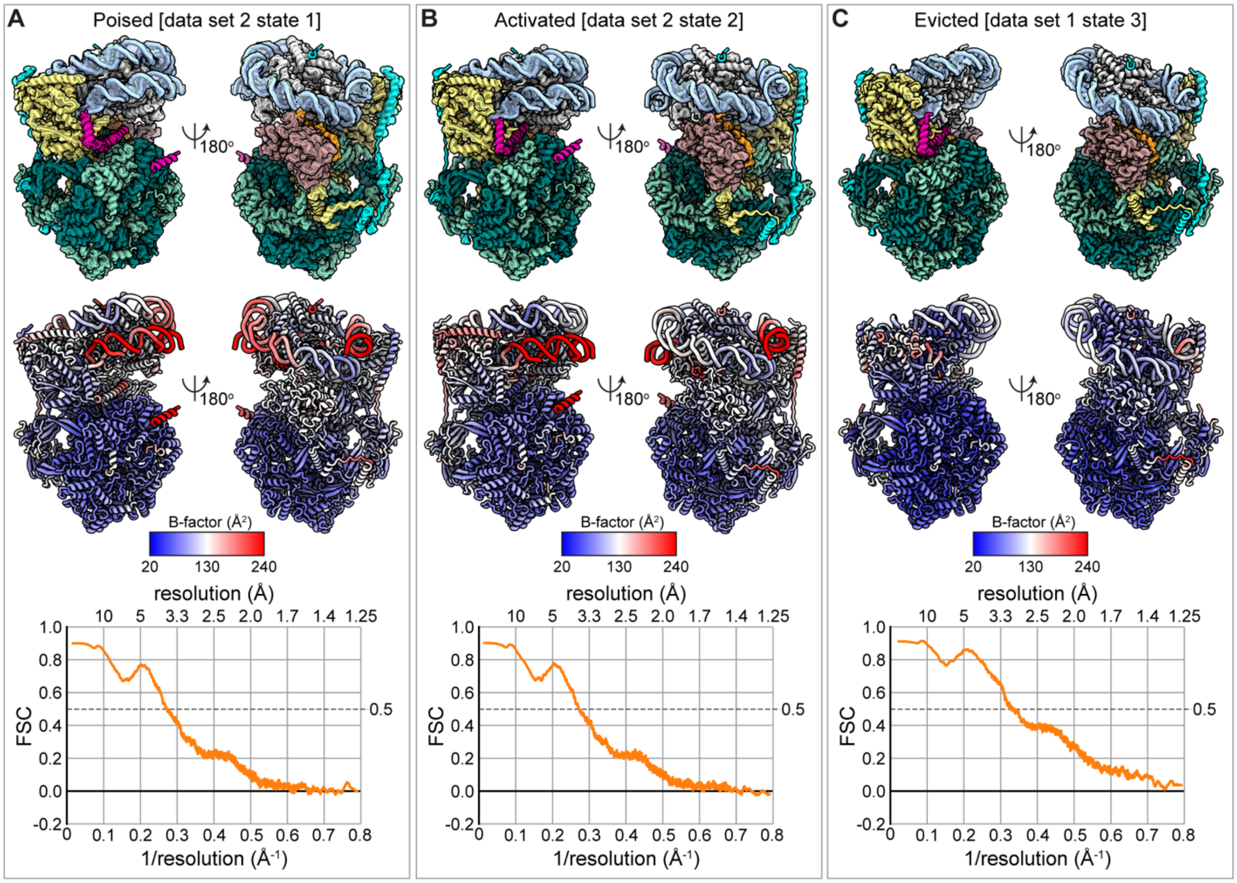

Fig. S19. Model building of the poised, activated, and evicted cryo-EM structures.
(A-C) Overall fit of refined SRCAP model (A, Poised; B, Activated; C, Evicted) into the high-resolution composite cryo-EM map (top). Refined model colored by PHENIX B-factor estimation (middle). Map vs. model Fourier shell correlation (FSC) shown below.

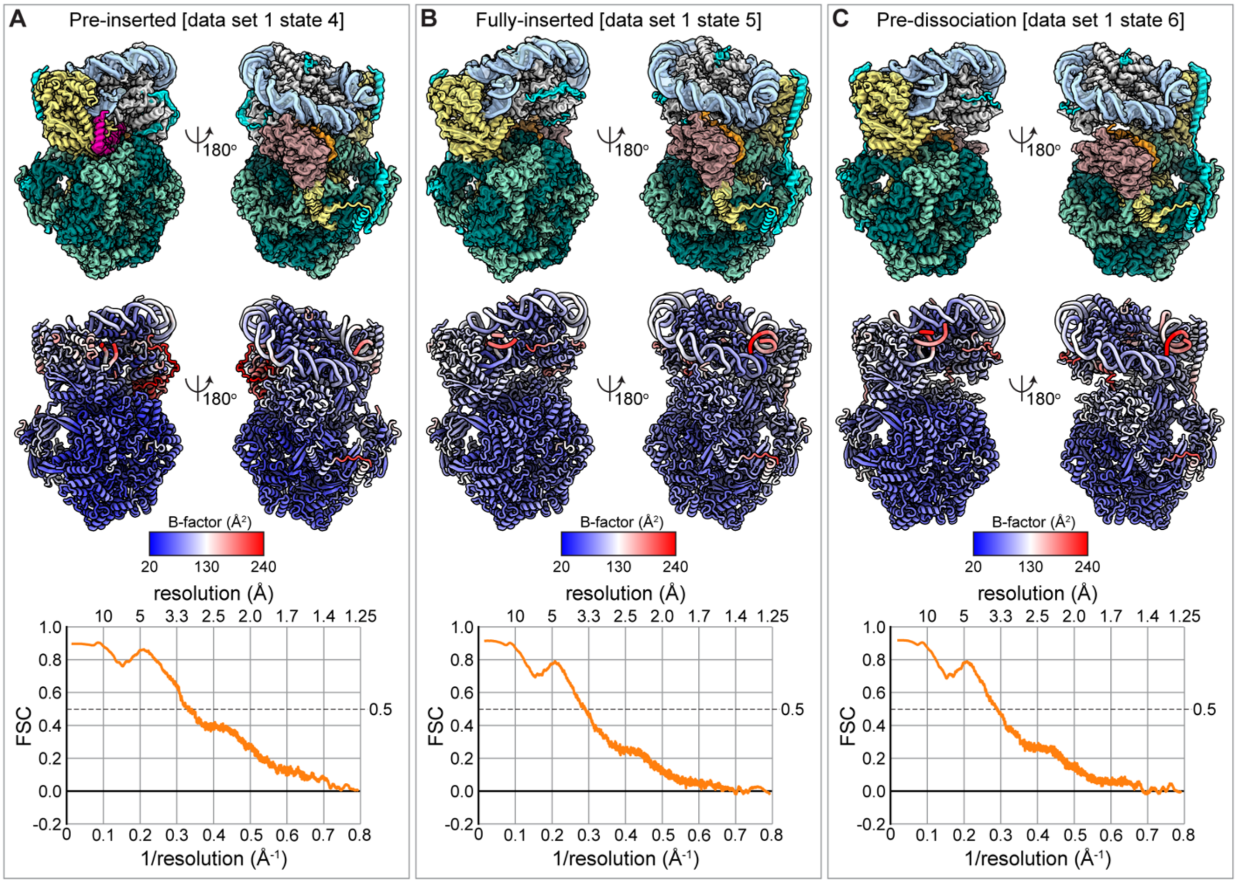

Fig. S20. Model building of the preinserted, fully inserted, and predissociation cryo-EM structures.

(A-C) Overall fit of refined SRCAP model (A, Preinserted; B, Fully inserted; C, Predissociation) into the high-resolution composite cryo-EM map (top). Refined model colored by PHENIX B-factor estimation (middle). Map vs. model Fourier shell correlation (FSC) shown below.

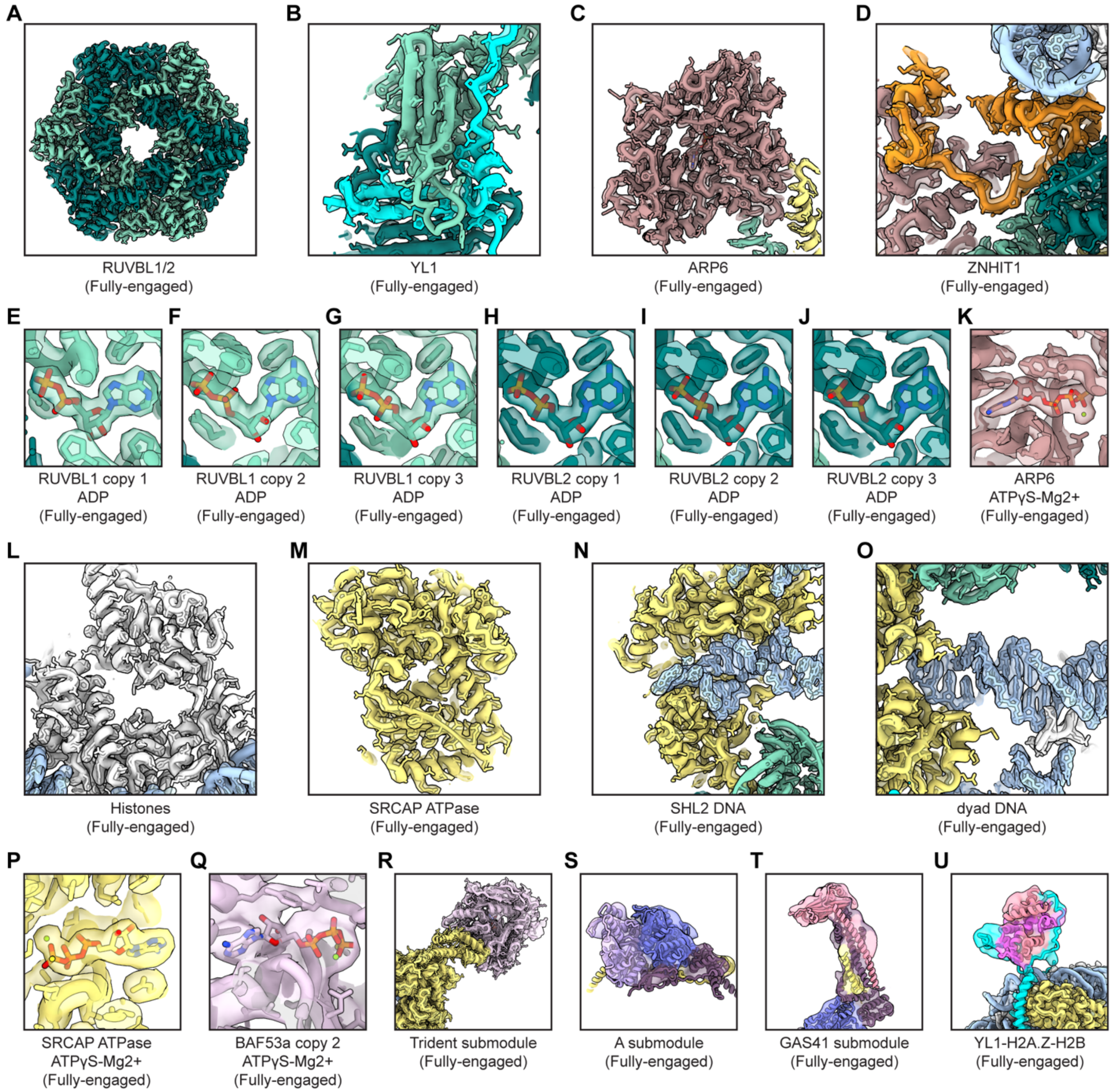

Fig. S21. Cryo-EM model-to-map fits of representative regions.
(A-U) Cryo-EM density (transparent) and atomic model of representative regions.

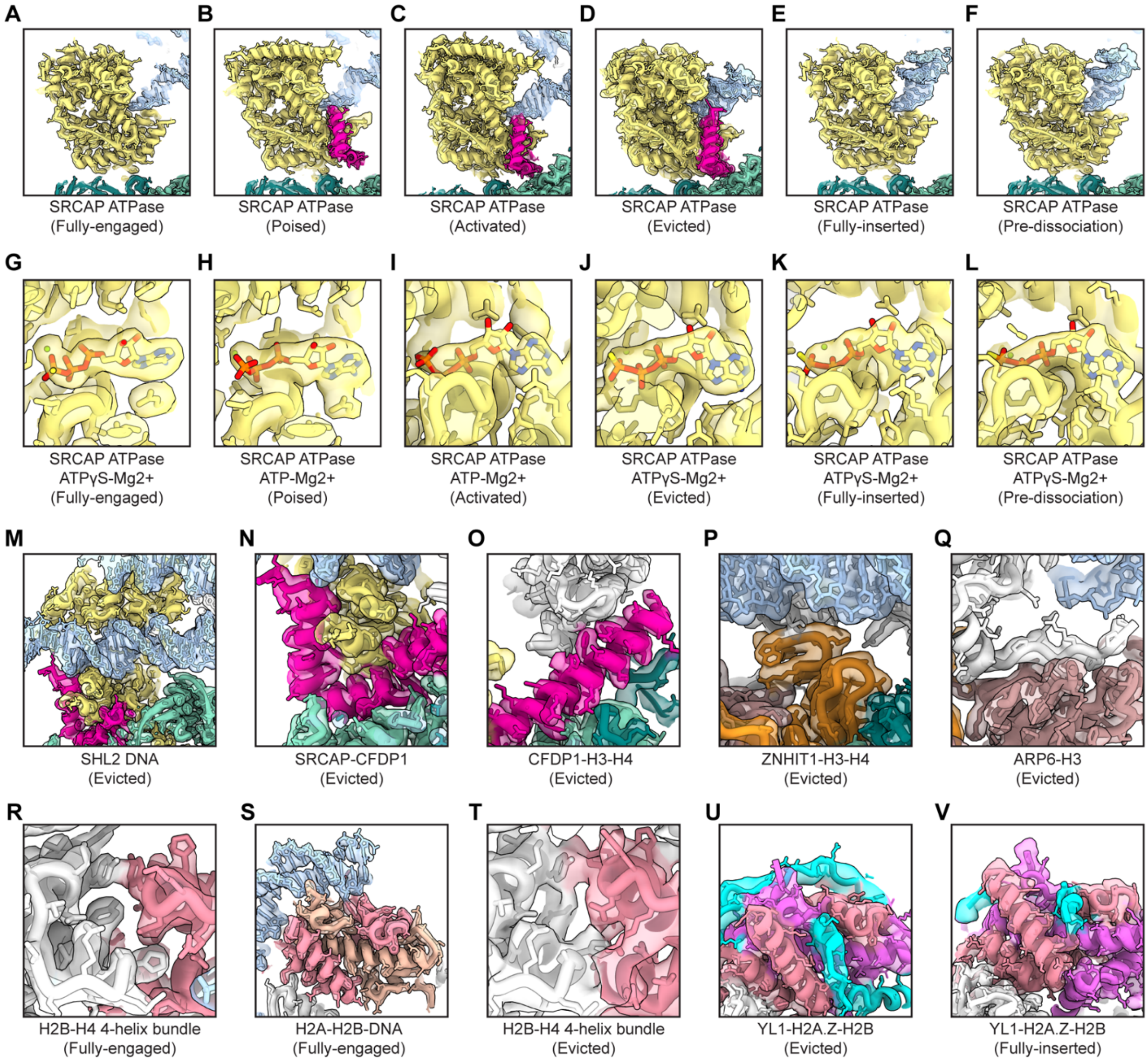

Fig. S22. Cryo-EM model-to-map fits of representative regions (continued).
(A-V) Cryo-EM density (transparent) and atomic model of representative regions.

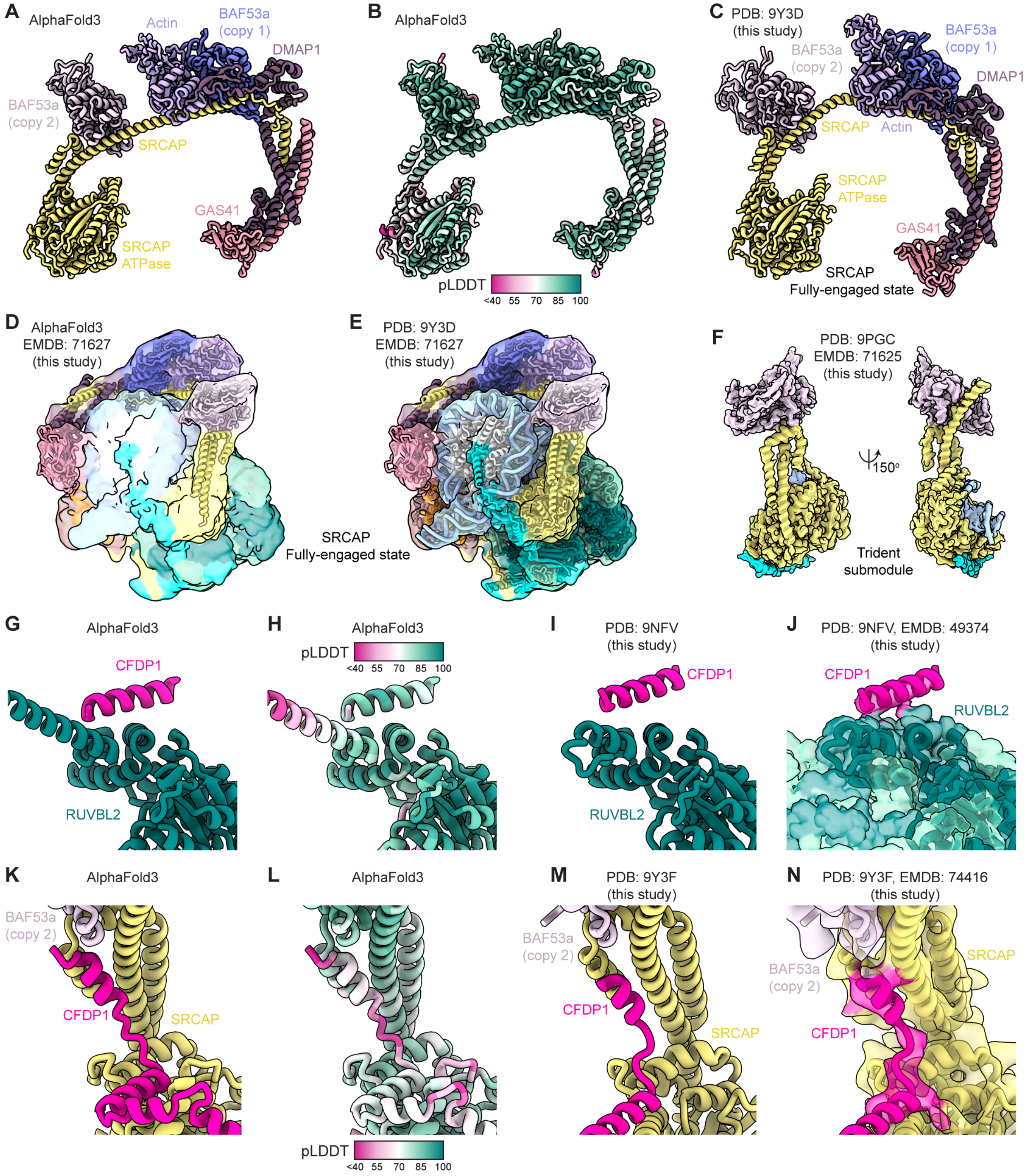

Fig. S23. Model building of the extended fully engaged SRCAP cryo-EM structure and poised SRCAP-CFDP1 cryo-EM structure.
(A) AlphaFold3-multimer prediction of the SRCAP HSA module. (B) Predicted structure in panel (A) colored by pLDDT confidence score. (C) Manually built model (PDB: 9Y3D) using cryo-EM data of the SRCAP fully engaged state. (D) Fit of the AlphaFold3-multimer prediction into the fully engaged state cryo-EM density (EMDB: 71627). (E) Fit of the manually built model (PDB: 9Y3D) into the fully engaged state cryo-EM density (EMDB: 71627). (F) Cryo-EM map (EMDB:71625) and model (PDB: 9PGC) of the Trident submodule. (G) AlphaFold3-multimer prediction of RUVBL2-CFDP1(guide helix). (H) Predicted structure in panel (G)

colored by pLDDT confidence score. (I) Manually built model (PDB: 9NFV) using cryo-EM data of the SRCAP poised state. (J) Fit of the manually built model (PDB: 9NFV) into the poised state cryo-EM density (EMDB: 49374). (K) AlphaFold3-multimer prediction of SRCAP-BAF53a-CFDP1(BCNT-I). (L) Predicted structure in panel (K) colored by pLDDT confidence score. (M) Manually built model (PDB: 9Y3F) using cryo-EM data of the SRCAP poised state. (N) Fit of the manually built model (PDB: 9Y3F) into the poised state cryo-EM density (EMDB: 74416).

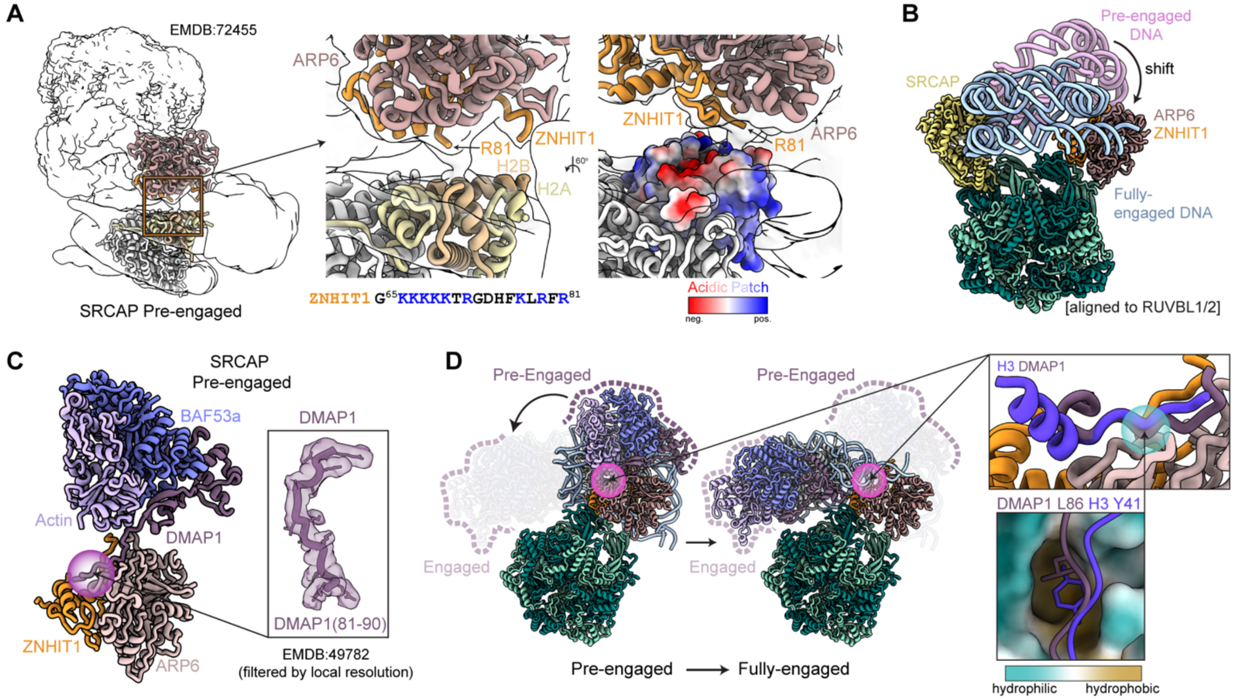

Fig. S24. Structural details of SRCAP preengaged state.
(A) Cryo-EM map of SRCAP preengaged shown as transparent surface showing ZNHIT1 interaction with the H2A-H2B dimer near the acidic patch. The H2A-H2B acidic patch is shown as a surface representation colored according to Coulombic electrostatic potential (red: -10 kcal/(mol·e), blue: +10 kcal/(mol·e)). (B) Conformational shift of the preengaged nucleosome (pink) to the fully engaged nucleosome (blue). The two states were aligned to the RUVBL1/2 core. (C) Isolated view of the A module is shown with the DMAP1 N-terminal region bound by ZNHIT1/ARP6 highlighted with a purple sphere. The cryo-EM density corresponding to DMAP1 residues 81-90 that are bound in the pocket is shown. (D) The conformational shift of the HSA module in the transition from preengaged to fully engaged is shown. The ARP6 surface used for binding DMAP1 (preengaged) and H3 tail (fully engaged) are highlighted with a pink sphere. A close-up of the interface is shown in the box with the hydrophobic pocket highlighted with a cyan sphere. The pocket is colored based on the molecular lipophilicity potential (MLP) map with cyan representing hydrophilic and brown representing hydrophobic.

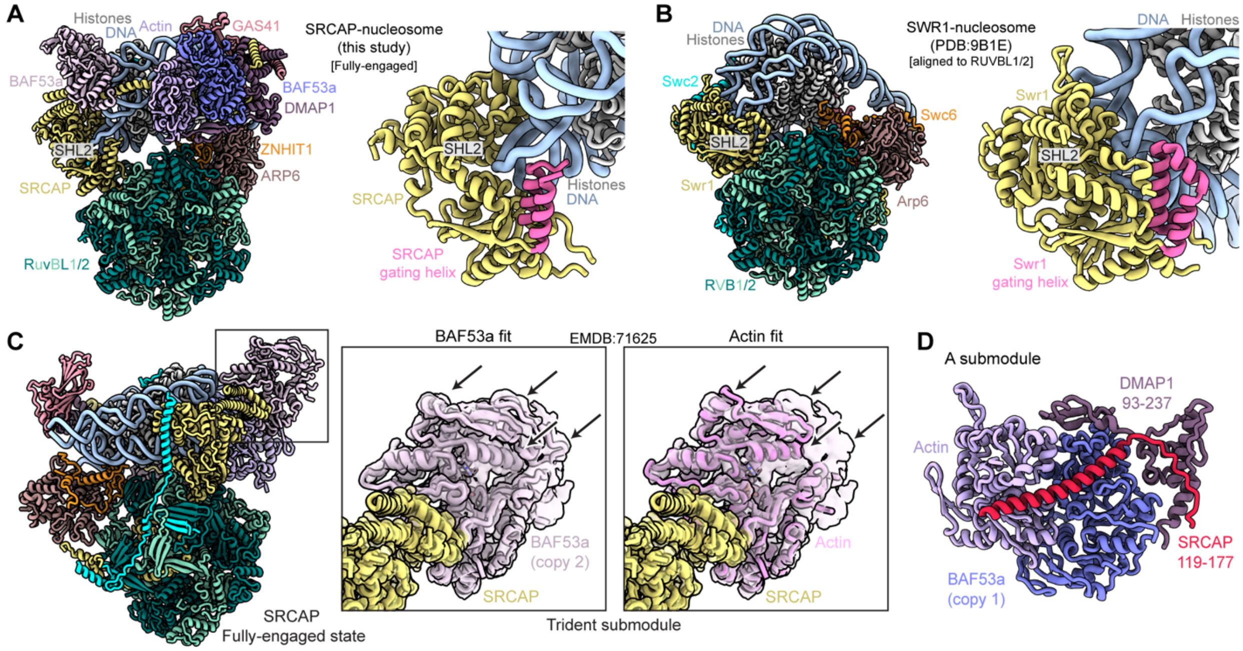

Fig. S25. Comparison of yeast SWR1 with human SRCAP (fully engaged), cryo-EM map-model-fit of BAF53a (copy 2) of the Trident submodule, and cryo-EM structure of the A submodule.
(A) Structure of fully engaged SRCAP-nucleosome complex (this study) and isolated view (right) of SRCAP ATPase bound to SHL2. The gating helix is colored pink. (B) Structure of SWR1-nucleosome complex (PDB: 9B1E) aligned to the RUVBL1/2 heterohexamer of SRCAP (panel a) and isolated view (right) of SWR1 ATPase bound to SHL2. The gating helix is colored pink. (C) Cryo-EM map-model fit of the BAF53a (copy 2) of the Trident submodule. The arrows indicate regions of the cryo-EM map that support the presence of BAF53a over Actin. (D) Isolated view of the A submodule of SRCAP.

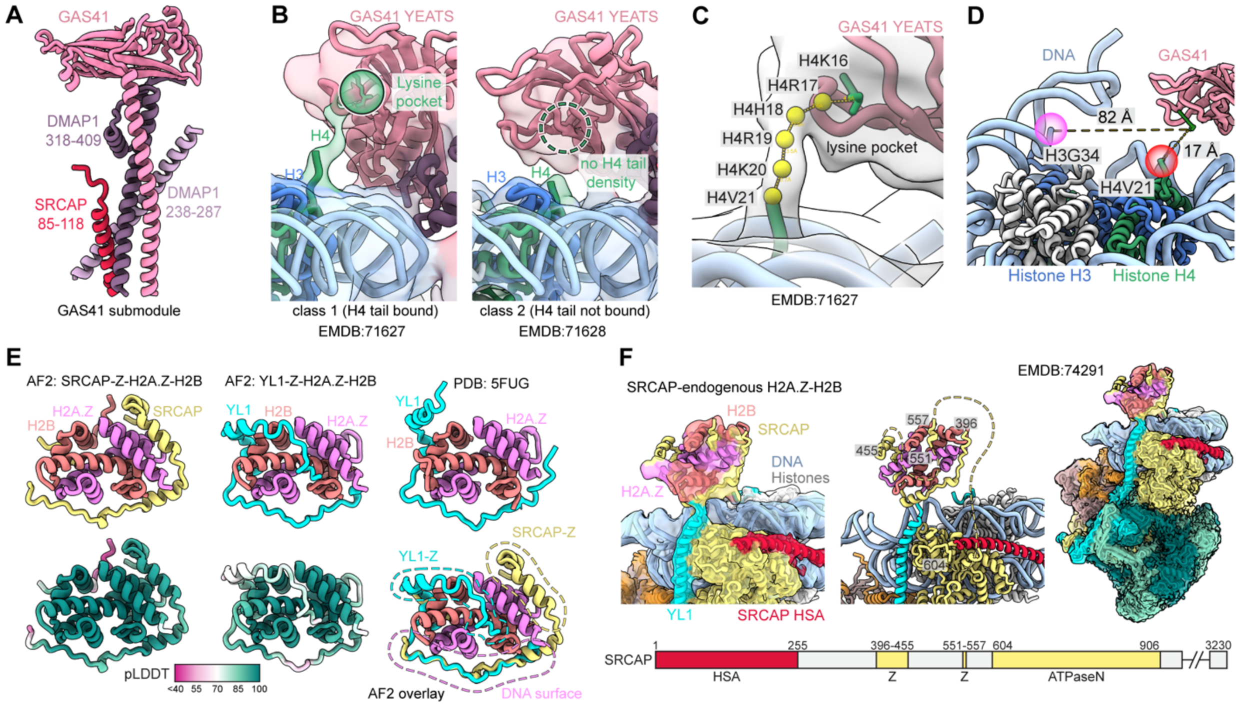

Fig. S26. Structural details of the GAS41 submodule and H2A.Z-H2B dimer.
(A) Isolated view of the GAS41 submodule of SRCAP. (B) Cryo-EM map of the H4 tail bound (class 1) and unbound (class 2) are shown with the lysine pocket of GAS41 YEATS domain highlighted green. The H4 tail is shown as a dashed green line. (C) Cryo-EM map of the H4 tail binding to GAS41 YEATS domain. The distance suggests H4K16 is bound in the lysine pocket of YEATS domain. (D) Structure of SRCAP fully engaged showing the YEATS domain and the linear distance from H3 tail (H3R40, pink) and H4 tail (H4V21, red). (E) Comparison of the crystal structure (PDB: 5FUG) and AlphaFold2-multimer predictions of H2A.Z-H2B chaperone domains of SRCAP complex. The predicted structures are colored by pLDDT confidence score (bottom). The overlay of the YL1 and SRCAP chaperoned H2A.Z-H2B shows an overlapping region chaperoning the DNA interaction surface and unique regions chaperoning the histone interaction surface. (F) Putative docking of SRCAP-Z-H2A.Z-H2B AlphaFold2-multimer model into low resolution cryo-EM density adjacent to the SRCAP ATPase and HSA helix. Domain map of SRCAP is shown below. The chaperone domains are labeled ‘Z’ and HSA helix as ‘HSA’.

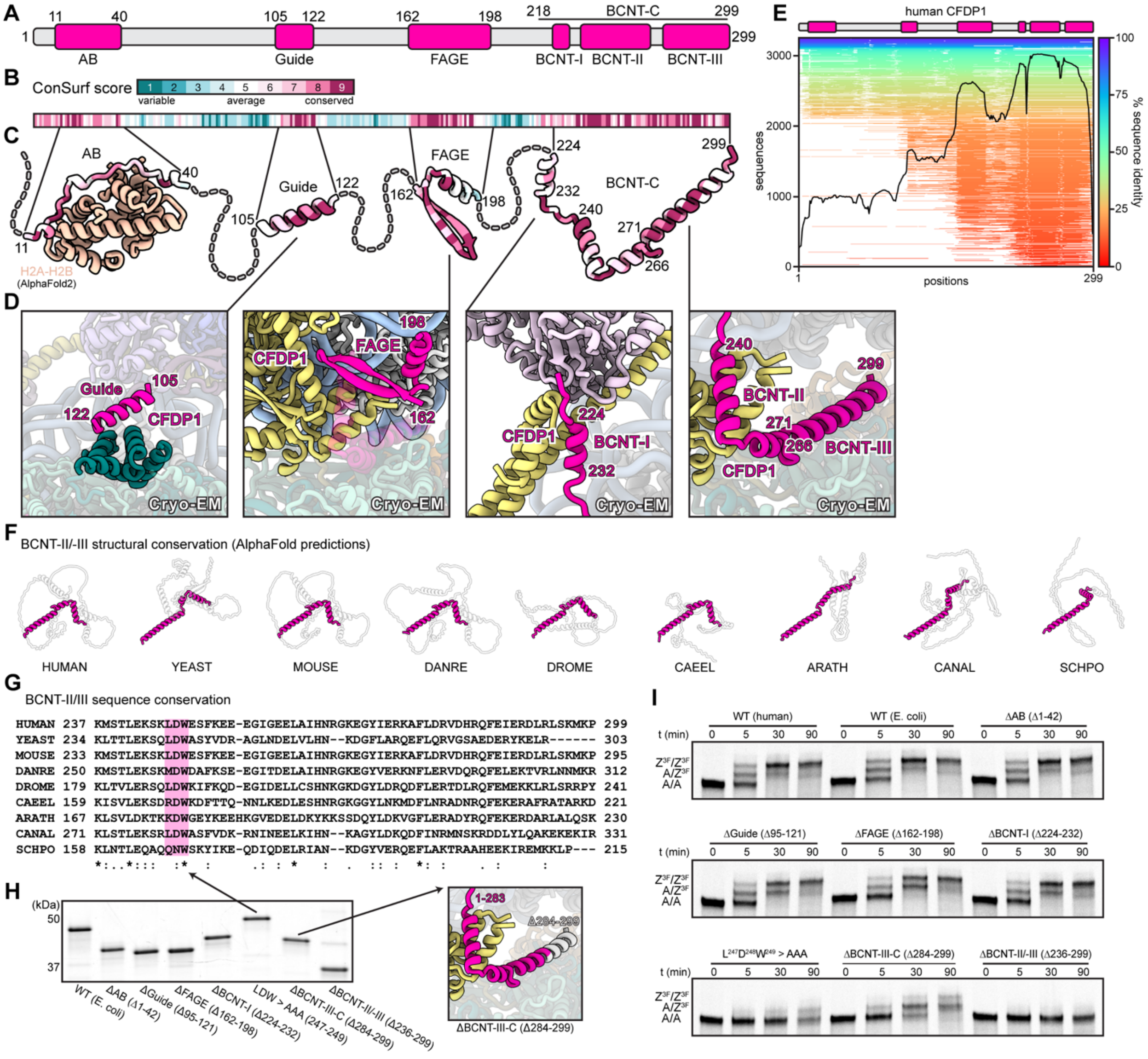

Fig. S27. Bioinformatic, biochemical, and structural characterization of CFDP1.
(A) Domain diagram of human CFDP1. (B) ConSurf evolutionary conservation scores (cyan: variable, white: average, maroon: conserved) of human CFDP1 (aligned to residues and domains in panel (A)). (C) Structured regions of CFDP1 generated with AlphaFold2 and cryo-EM structures colored based on ConSurf scores as in panel (B). (D) Structured regions of CFDP1 observed in cryo-EM structures are shown. (E) Multiple sequence alignment (MSA) of CFDP1 generated using ColabFold. (F) BCNT domain structural conservation predicted with AlphaFold2 run on the ColabFold server. The BCNT domain is colored pink and other regions are shown semi-transparent for clarity. (G) BCNT domain sequence conservation of model organisms aligned using Clustal Omega web server (‘*’: absolute conservation, ‘:’: strong conservation, ‘.’: weaker conservation). The conserved ‘LDW’ motif is highlighted in pink. (H) SDS-PAGE of bacterially purified WT and mutant CFDP1 proteins. The arrow for LDW > AAA (247-249) indicates mutation of the highly conserved domain. The inset for ΔBCNT-III-C (Δ284-299) shows the C-terminal truncation in white (rest of CFDP1 colored pink). (I) Histone exchange assay results of WT and mutant CFDP1 proteins at different time points (0, 5, 30, 90 minutes).

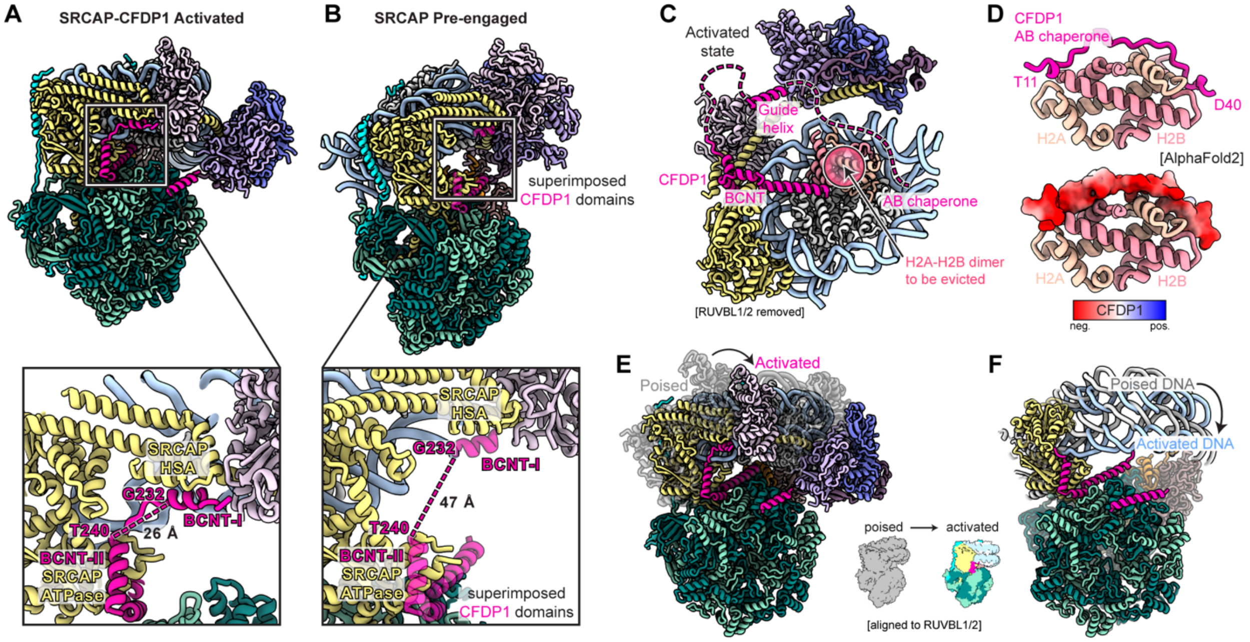

Fig. S28. Structural details of the SRCAP-CFDP1-nucleosome complex in the activated state.

(A) Cryo-EM structure of SRCAP-CFDP1-nucleosome ternary complex highlighting CFDP1 BCNT-I and BCNT-II domain interaction with SRCAP 'trident' submodule and $SRCAP^{ATPase}$, respectively. The inset shows a zoom in of the short linker (7 amino acids) tightly connecting the BCNT-I and II helices. (B) Cryo-EM structure of SRCAP-nucleosome complex in the preengaged state with superimposed CFDP1 BCNT-I and BCNT-II domains. The inset shows incompatibility of BCNT-I and II helices simultaneously binding to SRCAP 'trident' submodule and $SRCAP^{ATPase}$, respectively, prior to nucleosome engagement by SRCAP. (C) View of SRCAP-CFDP1-nucleosome with RUVBL1/2 core removed for clarity. The CFDP1 guide helix positions the AB chaperone domain near the H2A-H2B dimer to be evicted (red sphere). (D) AlphaFold2 prediction of H2A-H2B-CFPD1 subcomplex. On the bottom, CFDP1 is represented as a surface and colored according to Coulombic electrostatic potential (red: -10 kcal/(mol·e), blue: +10 kcal/(mol·e)). (E) Conformational shift of SRCAP HSA module in transition from poised to activated state with the states aligned to the RUVBL1/2 core. The poised state is colored gray and shown as semi-transparent. (F) Conformational shift of DNA in transition from poised to activated state. The poised state DNA is colored gray and activated state DNA is colored light blue.

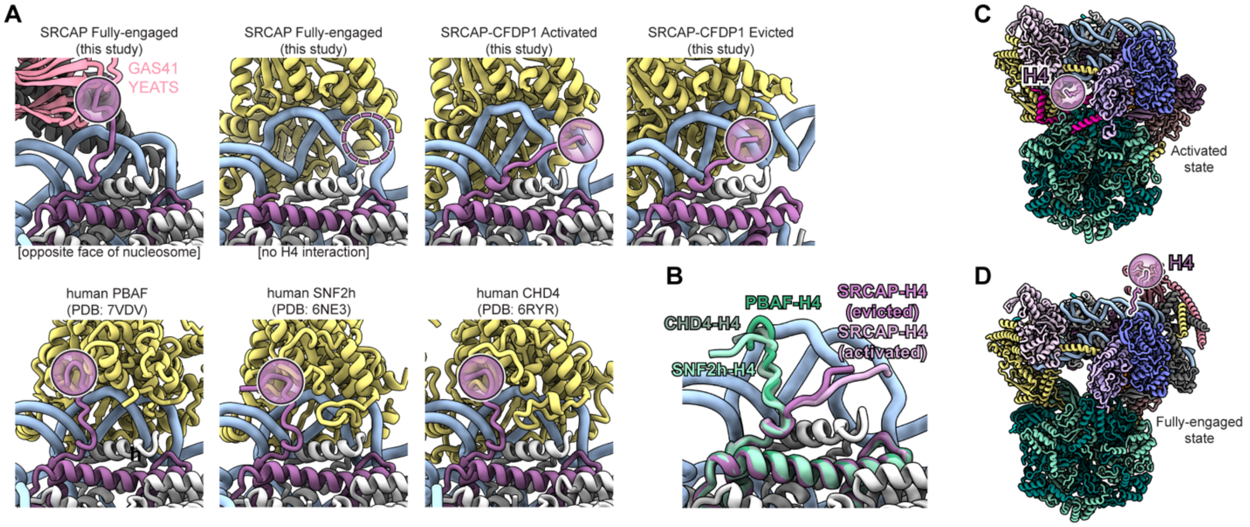

Fig. S29. Comparison of H4 binding by SRCAP and other chromatin remodelers.
(A) Comparison of H4 tail interaction with GAS41 (fully engaged), SRCAP ATPase (activated and evicted states), SNF2h (PDB: 6NE3), CHD4 (PDB: 6RYR), and PBAF (PDB: 7VDV). SRCAP ATPase in the engaged state (prior to CFDP1 binding) does not interact with H4 tail. (B) H4 tails bound by different remodelers are shown with other subunits removed for clarity. (C) Cryo-EM structure of the activated state, highlighting the H4 tail bound by the SRCAP ATPase. (D) Cryo-EM structure of the fully engaged state, highlighting the H4 tail bound by the GAS41 subunit. The structure is aligned to the RUVBL1/2 core of the activated state in panel (C), highlighting SRCAP engagement with both H4 tails of the nucleosome.

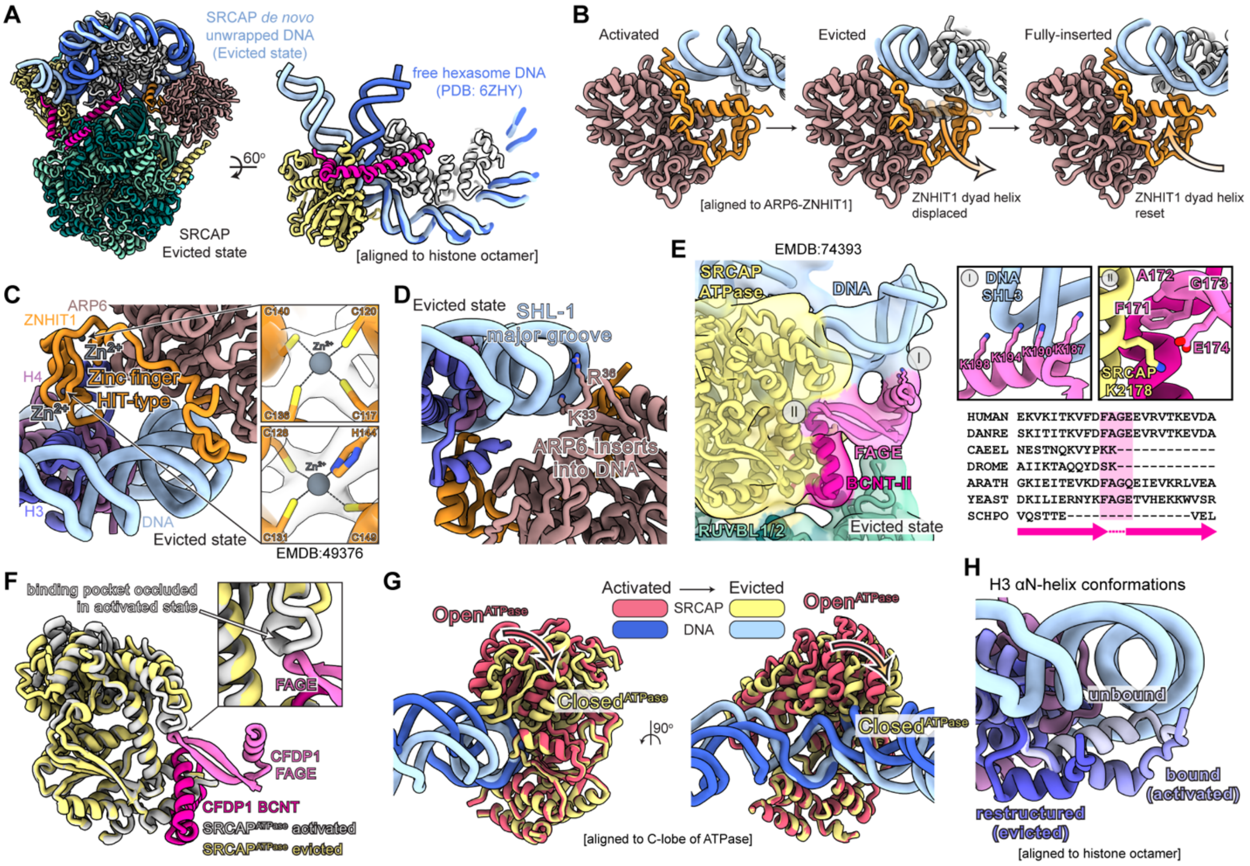

Fig. S30. Structural details of SRCAP-CFDP1-hexasome complex in the evicted state.
(A) Comparison of DNA in a free hexasome (PDB: 6ZHY, only the hexasome DNA is shown for clarity) and SRCAP unwrapped *de novo* hexasome (structures aligned to histone hexamer). (B) Conformation of ZNHIT1 dyad helix in the activated, evicted, and inserted states (aligned to ARP6). (C) The zinc finger HIT-type domain of ZNHIT1 contacts the exposed H3-H4 tetramer (region II). Panels (right) show the cryo-EM map and model of two zinc atoms coordinated by seven cysteines and one histidine of the HIT-type Zinc finger domain. (D) ARP6 K^{33} and R^{36} interaction with DNA major groove near SHL-1 in the evicted state. (E) Cryo-EM map (low-pass filtered) and fitted model of CFDP1 FAGE domain in the evicted state. Roman numerals are used to label different regions of the complex discussed in close-up panels (right). Panel I shows lysine residues of the FAGE domain interacting with unwrapped DNA near SHL3. Panel II shows the $F^{171}AGE^{174}$ residues that interact with the $SRCAP^{ATPase}$. (F) Comparison of the $SRCAP^{ATPase}$ FAGE domain binding pocket shows occlusion in the activated state which precedes the evicted state. The $SRCAP^{ATPase}$ in the activated state is colored white and $SRCAP^{ATPase}$ in the evicted state is colored yellow and the two states are aligned to the C-lobe of $SRCAP^{ATPase}$. (G) Open (activated) and closed (evicted) states of the $SRCAP^{ATPase}$ (aligned to C-lobe of $SRCAP^{ATPase}$) shown in two different views. The subunits in each state are colored according to the key above. (H) Superposition of H3 αN-helix in the unbound, ARP6-'bound' (activated state), and ARP6-'restructured' (evicted state) conformations (aligned to histone octamer).

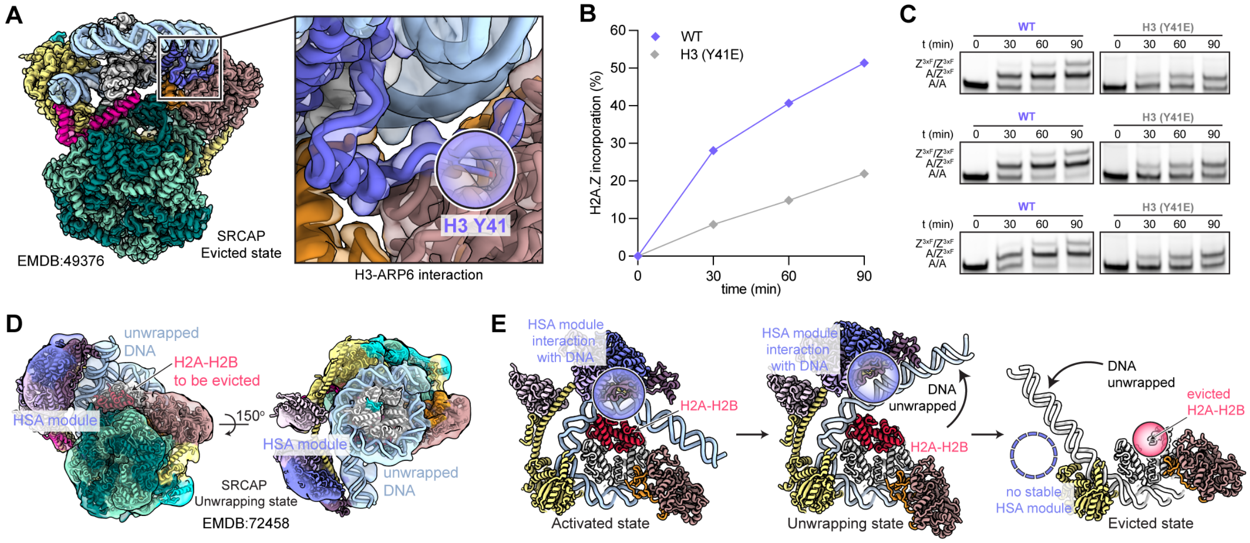

Fig. S31. Biochemical analysis of H3-ARP6 interaction and structural details of the SRCAP-CFDP1-nucleosome unwrapping state.
(A) Cryo-EM map and model of the evicted state. The inset shows a zoomed in view of the H3-ARP6 interaction with the H3 Y41 highlighted (purple sphere). (B) Quantification histone exchange assays in panel (C), showing reduced H2A.Z incorporation into H3 Y41E mutant (grey) nucleosome compared to WT (purple) nucleosomes. (C) Histone exchange assay results at different time points (0, 30, 60, 120 minutes) for WT (left) and H3 mutant (Y41E, right) nucleosomes. Triplicates are shown. (D) Cryo-EM density and model of the unwrapping state is shown with the HSA helix, unwrapped DNA, and H2A-H2B to be evicted labeled. (E) Comparison of the DNA and HSA module in the activated (left), unwrapping (middle), and evicted (right) states. The HSA module interaction with DNA is highlighted (purple sphere). In the evicted state, there is no stable density for the HSA module and the H2A-H2B dimer has been evicted (red sphere).

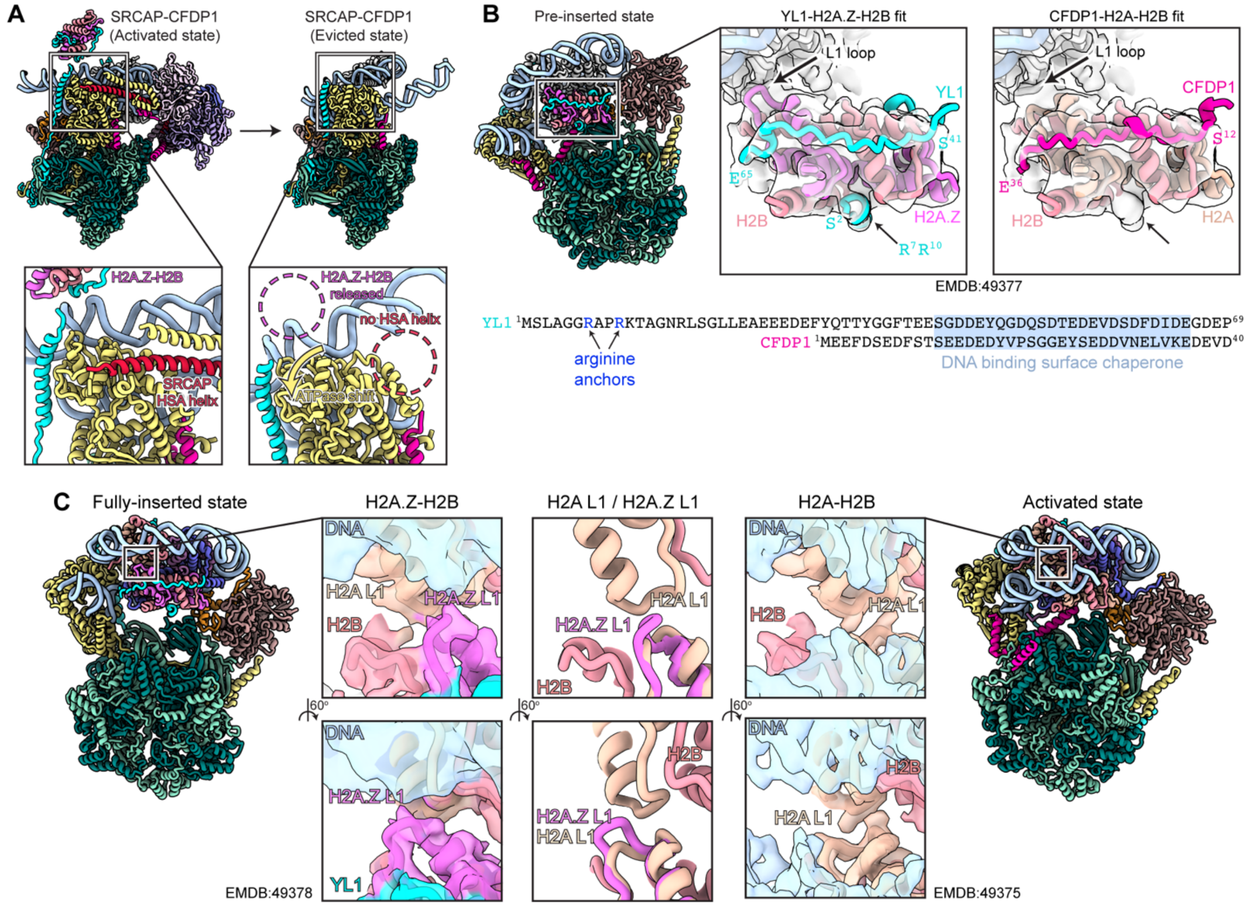

Fig. S32. Structural basis for H2A.Z-H2B release and insertion into the hexasome.
(A) Cryo-EM structures of SRCAP-CFDP1 in the activated state (with H2A.Z-H2B aligned as in the fully engaged state) and evicted state. The $SRCAP^{ATPase}$ is zoomed in to show the conformational changes that occur in the transition which may release H2A.Z-H2B. (B) Cryo-EM map and model for YL1-H2A.Z-H2B fit or CFDP1-H2A-H2B. The arrows indicate the extended L1 loop of H2A.Z (top) and arginine anchors of YL1 (bottom) that support presence of YL1-H2A.Z-H2B over CFDP1-H2A-H2B. Below, the sequence of the N-terminal region of YL1 and CFDP1 are shown. Only YL1 contains the arginine anchors observed in the cryo-EM density, confirming pre-insertion of H2A.Z-H2B. (C) Cryo-EM structure of the fully inserted state (left) and zoom-in of the H2A.Z L1 region (shown at two rotated views). Cryo-EM structure of the activated state (right) and zoom-in of the H2A L1 region (shown at the same views as for H2A.Z L1). Comparison of the cryo-EM density and structures (middle) confirms insertion of H2A.Z-H2B into the hexasome in the fully inserted state.

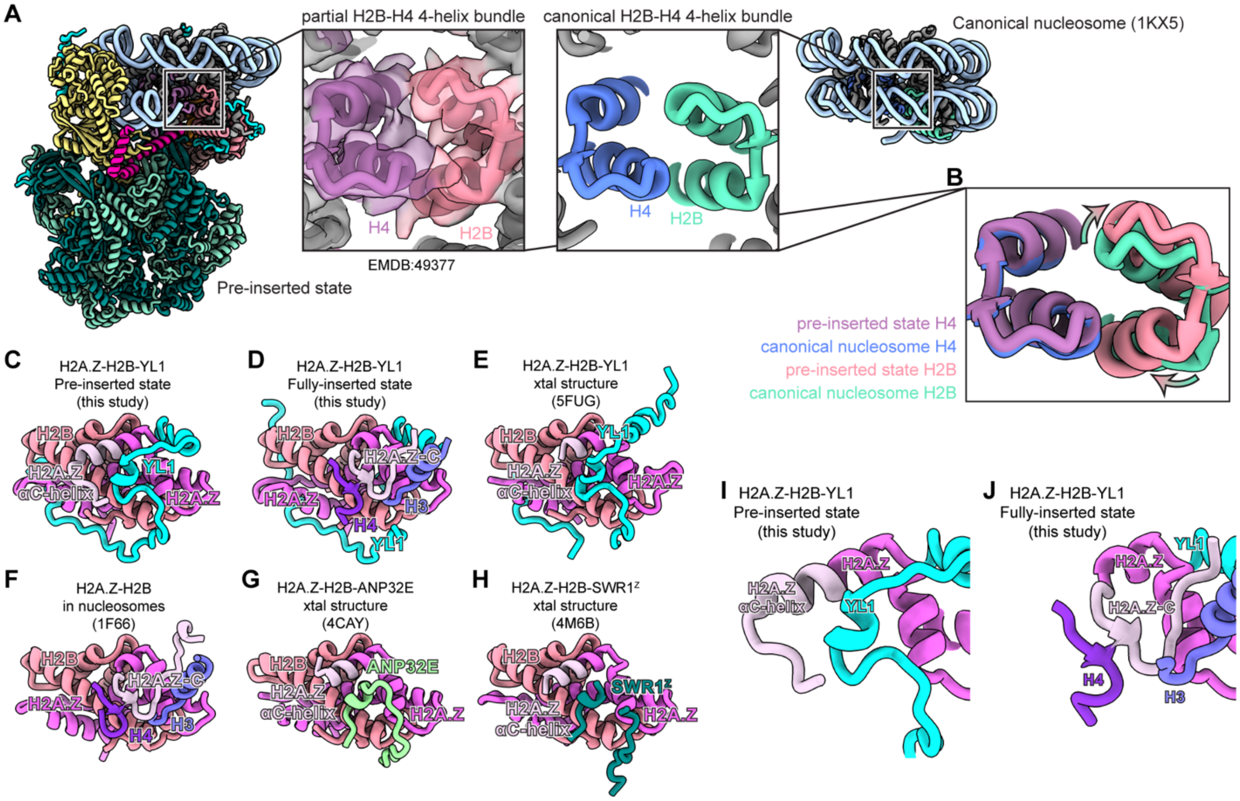

Fig. S33. Structural details of the preinserted and fully inserted H2A.Z-H2B dimer. (A) Structure of SRCAP preinserted state (left) with a zoom-in view (inset) of the partial H2B-H4 4-helix bundle (cryo-EM map and model shown). Structure of the canonical nucleosome (PDB: 1KX5, right) with a zoom-in view (inset) of the canonical H2B-H4 4-helix bundle (model shown). (B) Comparison of the partial and canonical H2B-H4 4-helix bundle. The preinserted H4 and H2B are colored purple and pink, respectively. The canonical nucleosome H4 and H2B are colored blue and green, respectively. (C) Cryo-EM structure of H2A.Z-H2B-YL1 in the preinserted state (this study). The extended H2A.Z αC-helix is colored light pink. (D) Cryo-EM structure of H2A.Z-H2B-YL1 in the inserted state (this study). The uncoiled H2A.Z C-terminus is colored light pink. (E) X-ray crystal structure of H2A.Z-H2B-YL1 in isolation (PDB: 5FUG). The extended H2A.Z αC-helix is colored light pink. (F) X-ray crystal structure of H2A.Z-H2B-YL1 in nucleosomes (PDB: 1F66). The uncoiled H2A.Z C-terminus is colored light pink. (G) X-ray crystal structure of H2A.Z-H2B-ANP32E in isolation (PDB: 4CAY). The extended H2A.Z αC-helix is colored light pink. (H) X-ray crystal structure of H2A.Z-H2B-SWR1^{Z} in isolation (PDB: 4M6B). The extended H2A.Z αC-helix is colored light pink. (I) Zoom in-view of H2A.Z-H2B-YL1 in the preinserted state (this study). The extended H2A.Z αC-helix and YL1 chaperone domain prevent H2A.Z C-terminus interactions with H3 and H4. (J) Zoom in-view of H2A.Z-H2B-YL1 in the inserted state (this study). YL1 chaperone domain is competed off by H3 and H4 which interact with the uncoiled H2A.Z C-terminus as observed in intact nucleosomes.

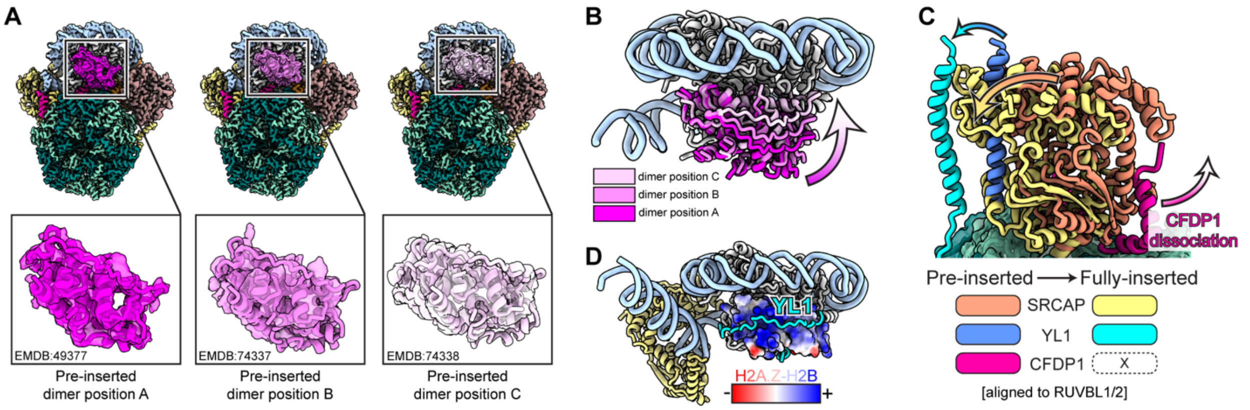

Fig. S34. Structural details of the transition from preinserted to fully inserted.
(A) Cryo-EM maps and structures of three positions of the preinserted H2A.Z-H2B dimer, which potentially depict the pathway of dimer insertion. The inset shows a zoom-in view of the cryo-EM map and model for the H2A.Z-H2B dimer. (B) Overlay of the three positions in panel (A) aligned to histone octamer. (C) Conformational transition of the SRCAP^{ATPase} and YL1 throttle helix in the preinserted and fully inserted states (aligned to RUVBL1/2 core). CFDP1 dissociates in the transition to the fully inserted state. The subunits in each state are colored according to the key below. (D) Structure of YL1 chaperone domain in the fully inserted state which protects the positively charged surface of H2A.Z-H2B. The exposed H2A.Z-H2B dimer is colored according to Coulombic electrostatic potential (red: -10 kcal/(mol·e), blue: +10 kcal/(mol·e)).

A

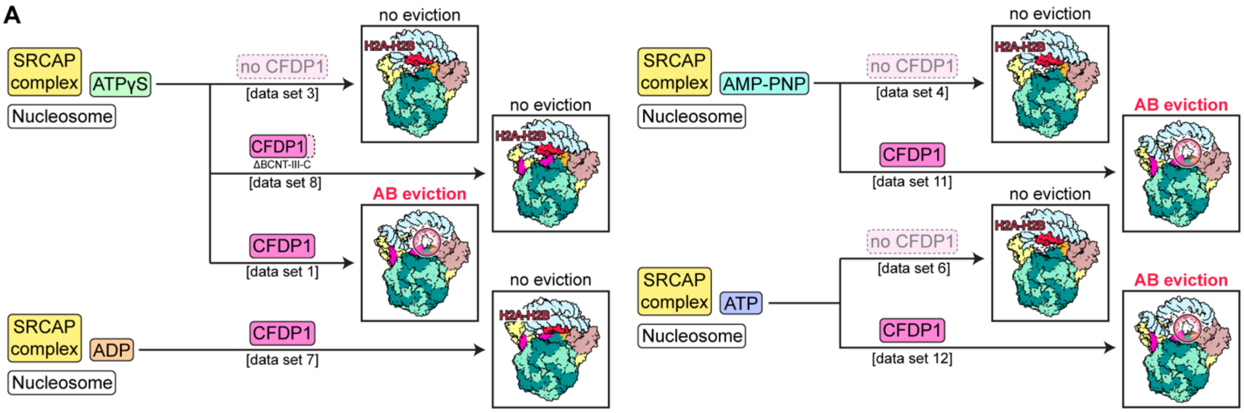

B

H2A-H2B eviction	-	+	-	-	+	-	+	+	+	-
SRCAP	+	+	+	+	+	+	+	+	+	+
Nucleosome	+	+	+	+	+	+	+	+	+	+
CFDP1	-	+	-	-	+	-	+	+	+	+
CFDP1(Δ284-299)	-	-	-	+	-	-	-	-	-	-
ATP	+	+	-	-	-	-	-	-	-	-
ATPγS	-	-	+	+	+	-	-	-	-	-
AMP-PNP	-	-	-	-	-	+	+	-	-	-
ADP-BeFx	-	-	-	-	-	-	-	+	-	-
ADP-Vi	-	-	-	-	-	-	-	-	+	-
ADP	-	-	-	-	-	-	-	-	-	+
Dataset	6	12	3	8	1	4	11	9	10	7

Fig. S35. Summary of cryo-EM datasets that show H2A-H2B eviction.
(A) Diagram showing sample composition of the various cryo-EM datasets (not all data sets are shown) and the resulting observed state. Only H2A-H2B evicted and un-evicted states are indicated for clarity. (B) Sample composition of various cryo-EM datasets (not all data sets are shown) and whether H2A-H2B eviction was observed (+) or not observed (-).

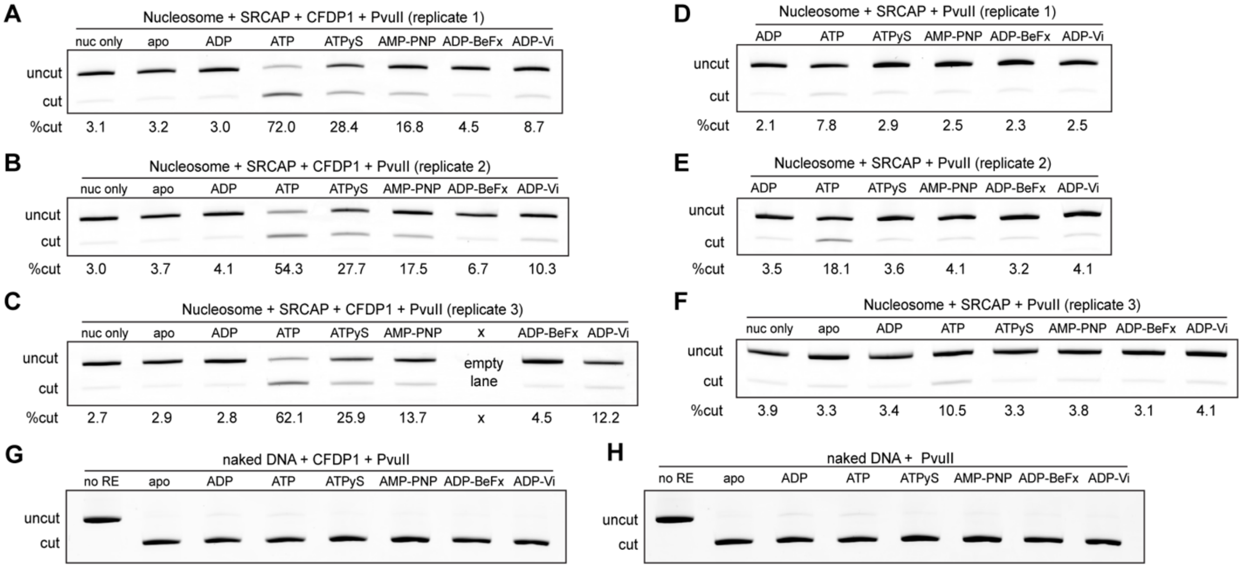

Fig. S36. Additional biochemical data for ATP hydrolysis-independent eviction.
(A) Restriction digest assay (replicate 1) with nucleosome, SRCAP, CFDP1, and PvuII restriction enzyme. Percent (%) of cut population is indicated below each lane. (B) Restriction digest assay (replicate 2) as in panel (A). (C) Restriction digest assay (replicate 3) as in panel (A). (D) Restriction digest assay (replicate 1) with nucleosome, SRCAP, and PvuII restriction enzyme (without CFDP1). Percent (%) of cut population is indicated below each lane. (E) Restriction digest assay (replicate 2) as in panel (D). (F) Restriction digest assay (replicate 3) as in panel (D). (G) Restriction digest assay with naked DNA (identical construct used to generate nucleosomes), CFDP1, and PvuII restriction enzyme. Essentially all DNA is digested indicating CFDP1 and nucleotide conditions (apo, ATP, and ATP analogs) have no inhibitory effect on PvuII restriction digest activity. (H) Restriction digest assay as in panel (G) but without CFDP1.

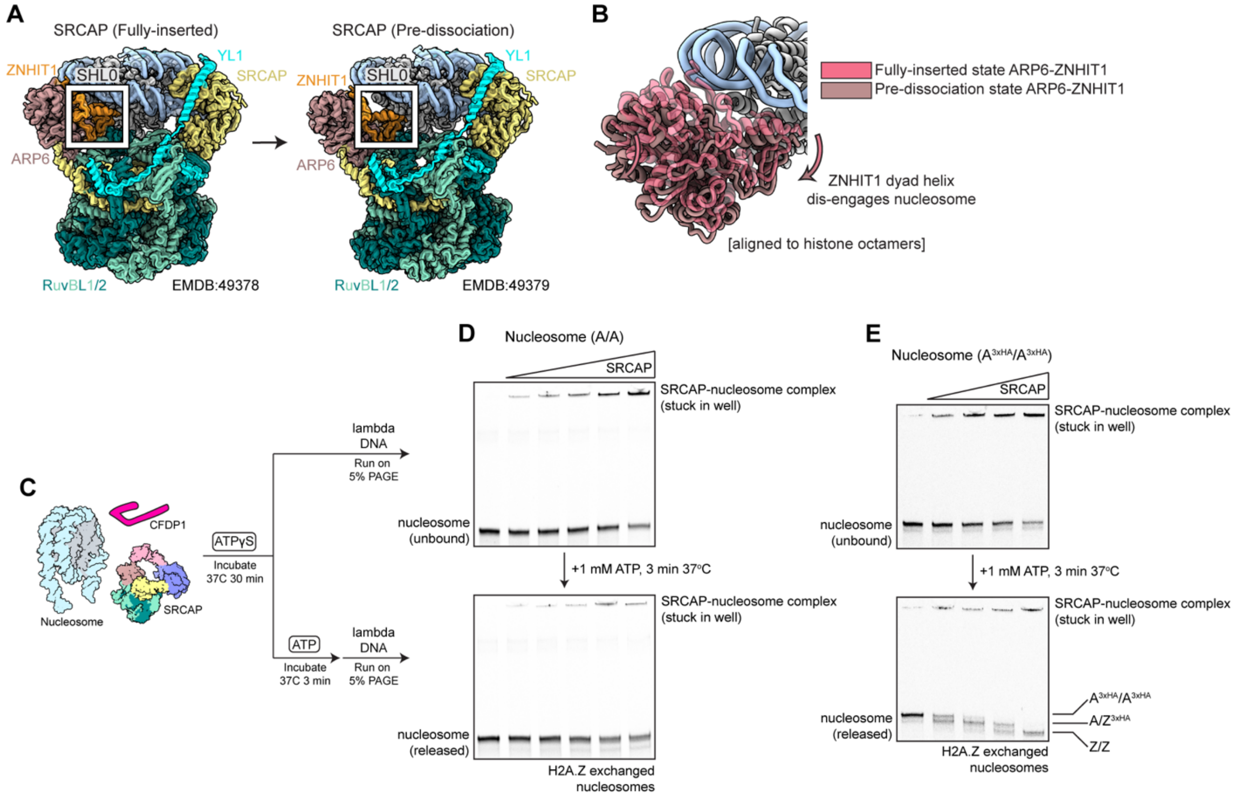

Fig. S37. Structural details of the predissociation state and biochemical data for ATP function in SRCAP dissociation.

(A) Cryo-EM maps and structures of SRCAP in the fully inserted and predissociation states. (B) Zoom in on the white boxes in panel (A) aligned to the histone octamer. The ARP6-ZNHIT1 is colored red in the inserted state and brown in the predissociation state. (C) Schematic for the biochemical ATP-chase dissociation assay in which the SRCAP–CFDP1–nucleosome ternary complex is first assembled in the presence of low ATPγS (0.1 mM), then challenged with ATP (1 mM) to induce disassembly. Lambda DNA is then added to the sample and run on native PAGE to resolve undissociated complexes (stuck in well) or dissociated and released free nucleosomes (runs into gel). (D) Results of the ATP-chase dissociation assay with untagged A/A nucleosomes. Addition of ATP releases complexes stuck in the well, indicating ATP hydrolysis induced dissociation. (E) Results of the ATP-chase dissociation assay with 3xHA tagged A/A nucleosomes. Addition of ATP releases complexes stuck in the well, indicating ATP hydrolysis induced dissociation. Additionally, the 3xHA tagged H2A allows for identification of endogenous H2AZ (untagged) incorporation.

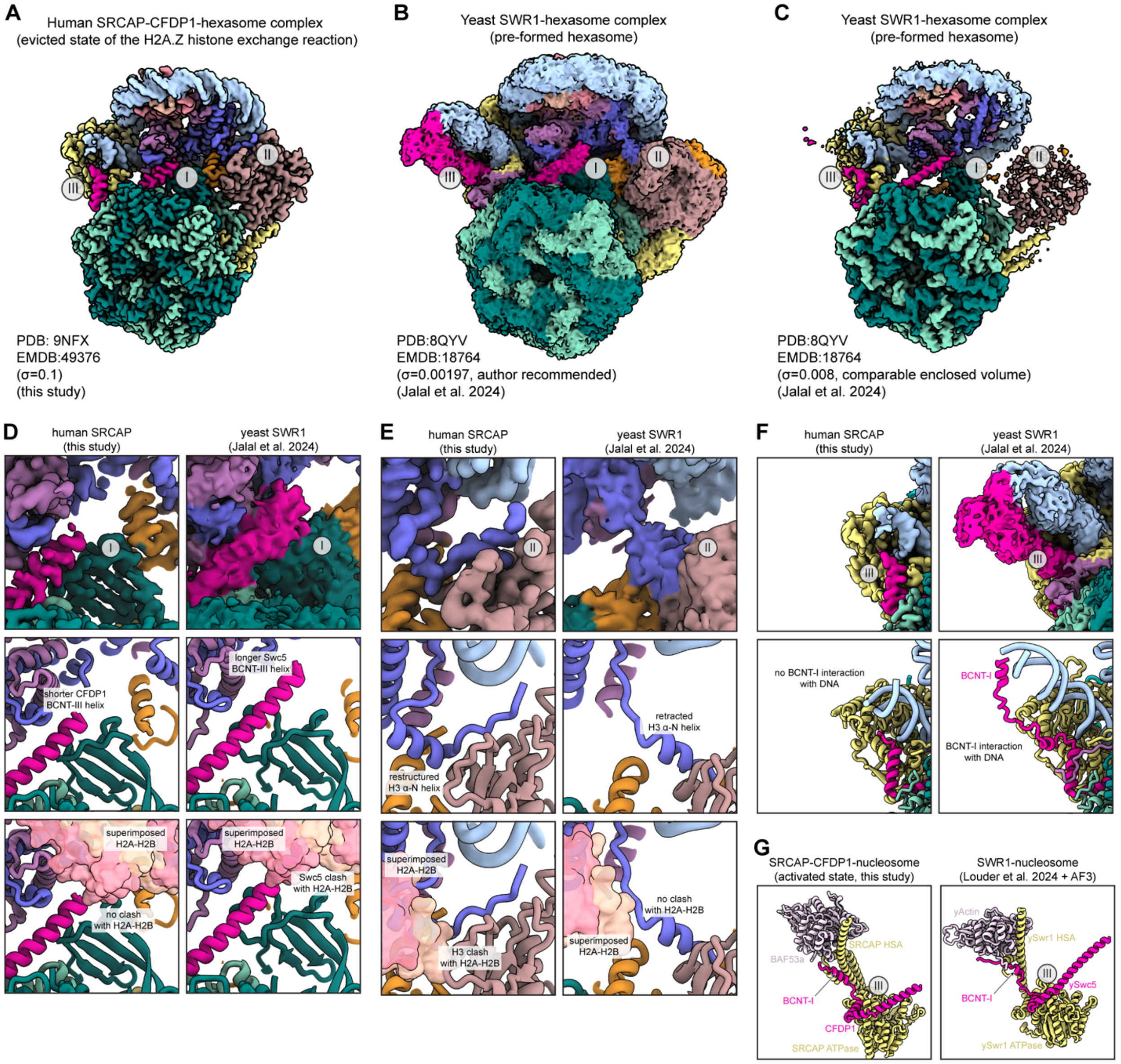

Fig. S38. Comparison of human SRCAP-CFDP1-hexasome (evicted state) and yeast SWR1-hexasome (pre-formed hexasome).

(A) Cryo-EM map of human SRCAP-CFDP1-hexasome (evicted state, EMDB:49376, contour level = 0.1, colored based on PDB:9NFX, this study). (B) Cryo-EM map of yeast SWR1-hexasome (pre-formed hexasome, EMDB:18764, author recommended contour level = 0.00197, colored based on PDB:8QYV) (*46*). (C) Cryo-EM map of yeast SWR1-hexasome (pre-formed hexasome, EMDB:18764, contour level = 0.008, colored based on PDB:8QYV) (*46*). (D) Comparison of CFDP1/Swc5 BCNT-III domain (region I). The cryo-EM map (top), cryo-EM structure (middle), and structure with superimposed H2A-H2B (aligned based on canonical nucleosome, bottom) are shown. The Swc5 BCNT-III helix is longer than CFDP1 BCNT-III helix and proposed to sterically clash with nucleosomal H2A-H2B dimer (*46*). (E) Comparison of H3-ARP6 interaction (region II). The cryo-EM map (top), cryo-EM structure (middle), and structure with superimposed H2A-H2B (aligned based on canonical nucleosome, bottom) are shown. The human ARP6 restructures the H3 αN-helix to evict H2A-H2B whereas yeast Arp6 retracts the H3 αN-helix. (F) Comparison of CFDP1/Swc5 BCNT-I domain (region III). The

cryo-EM map (top) and cryo-EM structure (bottom) are shown. Swc5 BCNT-I domain is proposed to bind the unwrapped linker DNA (Jalal et al. 2024) (*46*). This is in direct contrast to previous work showing BCNT-I domain interaction with the Swr1 HSA helix (panel G), which is not observed in the hexasome state (*29*). (G) Comparison of the conserved BCNT-I domain in SRCAP and SWR1 nucleosome bound structures. In both SRCAP and SWR1, the BCNT-I domain interacts with the SRCAP/Swr1 HSA domain and BAF53a/Actin to form the Trident submodule. This submodule is only stably observed in the SRCAP-CFDP1-nucleosome activated state.

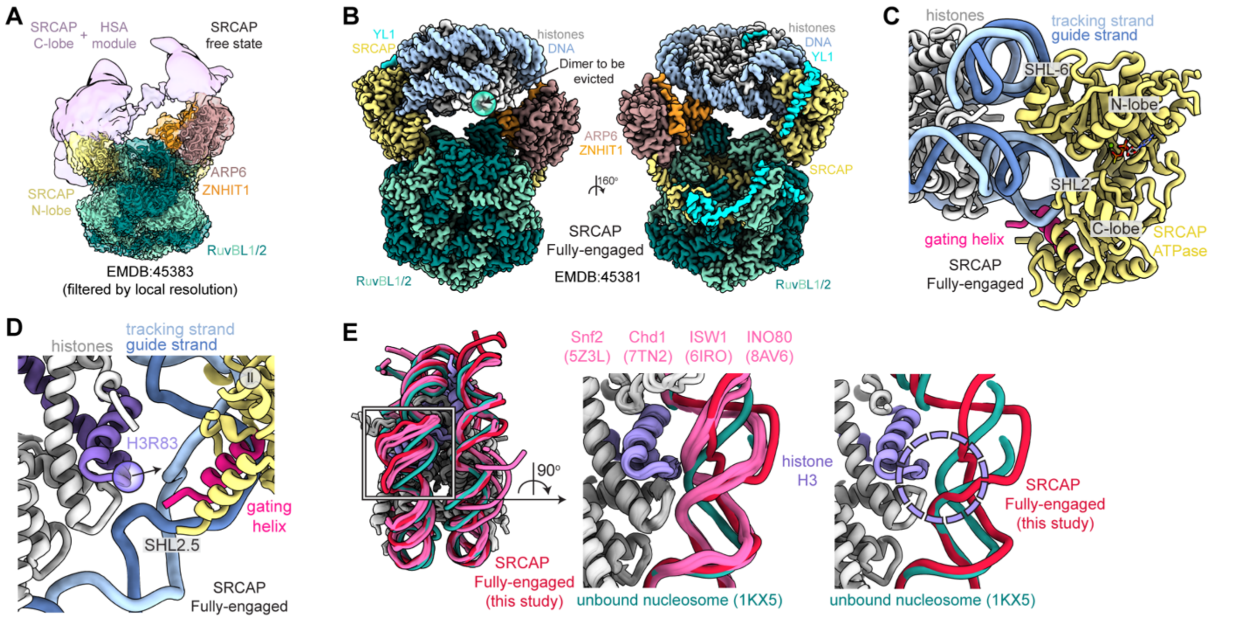

Fig. S39. Structural details of the SRCAP free and fully engaged states.
(A) Free SRCAP complex map and model at low threshold. The HSA module interaction with ARP6-ZNHIT1 is observed at lower thresholds. (B) Two views of the cryo-EM composite map of the core fully engaged SRCAP-nucleosome complex. The H2A-H2B dimer destined for eviction is labeled with a green sphere. (C) Close-up of SRCAP ATPase that binds primarily to SHL2 and forms additional contacts at SHL-6. The N- and C-lobe of the ATPase are labeled and the gating helix is colored pink. The tracking and guide strand of the nucleosome DNA are colored light and dark blue, respectively. The cryo-EM density of ATPγS-Mg^{2+} in the N-lobe nucleotide pocket is shown. Atoms are colored by elements (red: oxygen, blue: nitrogen, orange: phosphate, green: magnesium). (D) Close-up of the gating helix that inserted into the minor groove near SHL2.5 and distorts the DNA, disrupting histone H3 contacts (purple sphere). (E) Overlay of chromatin remodeler structures (Snf2, PDB: 5Z3L; Chd1, PDB: 7TN2, ISW1, PDB: 6IRO; INO80, PDB: 8AV6; all colored pink), free nucleosome (PDB: 1KX5; colored green), and SRCAP complex (this study; colored red) showing DNA distortion near SHL2.5 where the ATPase binds. SRCAP binding uniquely distorts the DNA and detaches DNA from histone H3 (colored purple).

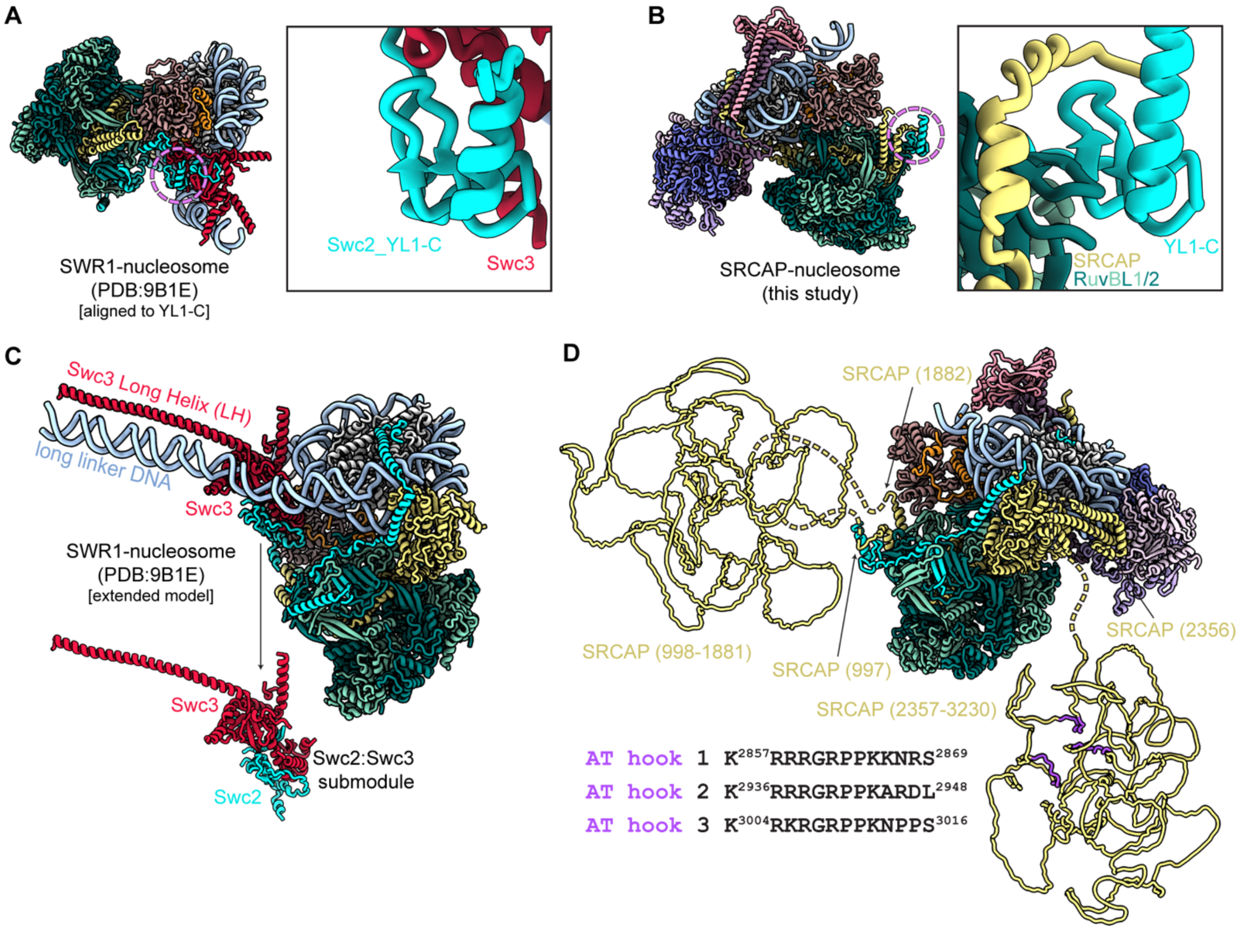

Fig. S40. Comparison of the yeast Swc2:Swc3 and human YL1:SRCAP submodules.
(A) SWR1-nucleosome structure (PDB:9B1E) and zoom-in of the Swc2:Swc3 submodule. (B) SRCAP-nucleosome structure (this study) and zoom-in of the YL1:SRCAP submodule.
(C) Extended model of SWR1-nucleosome (PDB:9B1E) highlighting the structured DNA binding elements in yeast Swc2:Swc3 submodule. Swc3 is shown as a surface representation colored according to Coulombic electrostatic potential (red: -10 kcal/(mol·e), blue: +10 kcal/(mol·e)). The yeast Swc2:Swc3 submodule is also shown in isolation (bottom). (D) Human YL1:SRCAP submodule lacks structured elements but contains a ~900 residue intrinsically disordered region (IDR). The C-terminus of SRCAP contains another ~900 residue IDR that contains three AT hooks (colored purple).

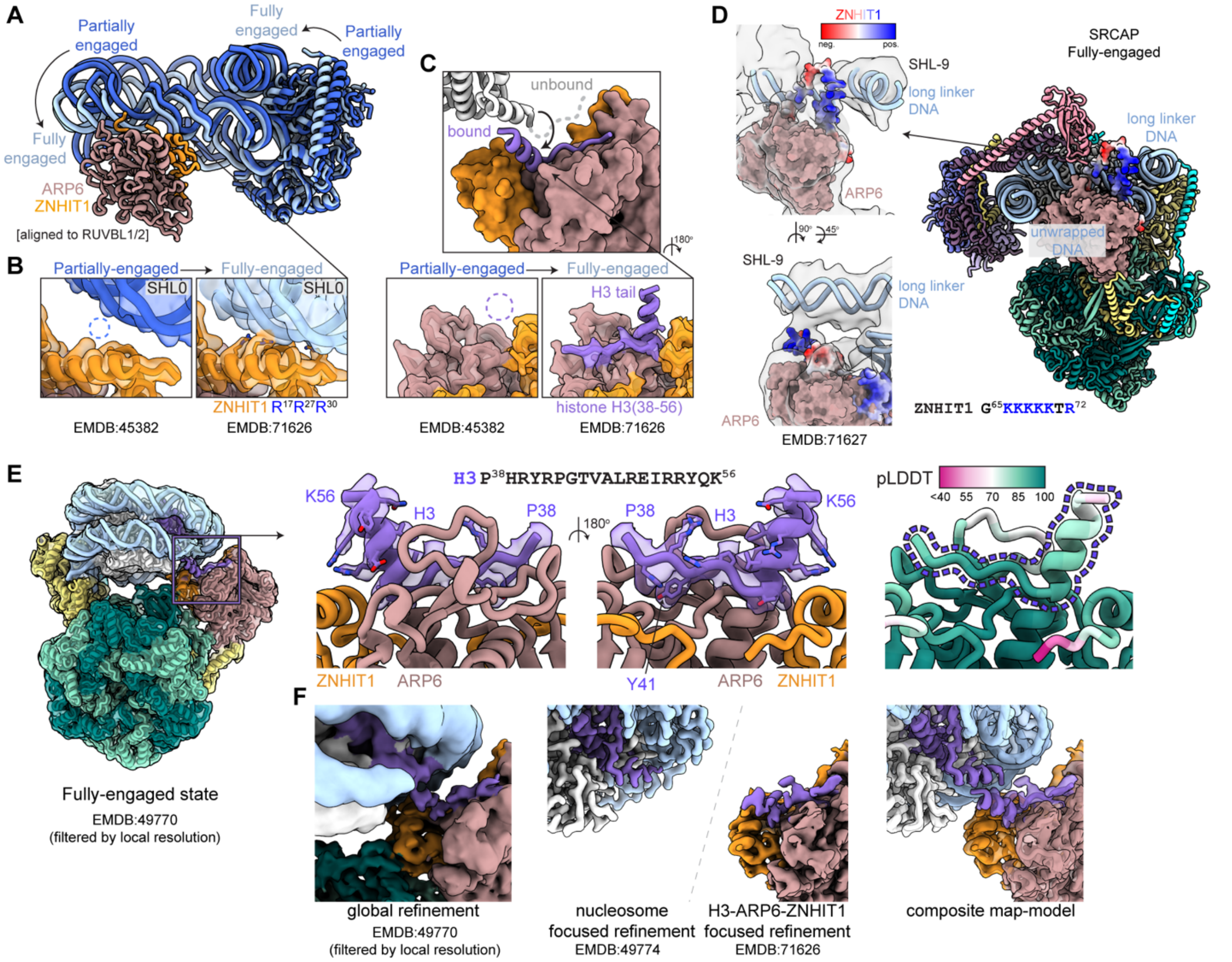

Fig. S41. Structural details of SRCAP partially engaged state and ARP6 interaction with histone H3.
(A) Isolated view of the nucleosome, ATPase, and ZNHIT1/ARP6 module showing the conformational change from partially engaged (dark blue) to fully engaged (light blue). The two states were aligned relative to the RUVBL1/2 core. (B) Zoom in views of ZNHIT1 showing dyad (SHL0) DNA binding in the fully engaged state. The cryo-EM map is shown as transparent and the model is shown as a cartoon representation. Atoms are colored by elements (blue: nitrogen). (C) Histone H3 tail binding by ARP6 is shown with unbound H3 colored white and bound H3 colored purple (top). Rotated and zoomed in views (bottom) show H3 tail binding in the fully engaged state. The cryo-EM map is shown as transparent and the model is shown as a cartoon representation. (D) The putative location of the highly positive loop of ZNHIT1 in the fully engaged state is shown. The cryo-EM map is shown transparent and ZHHIT1 is colored according to Coulombic electrostatic potential (red: -10 kcal/(mol·e), blue: +10 kcal/(mol·e)). (E) Structural details of histone H3 bound by ARP6-ZNHIT1 subunits of SRCAP. The cryo-EM map and model of bound H3 is shown. The AlphaFold2-multimer prediction used as a starting model is shown and colored by pLDDT confidence score. (F) The global refinement map, nucleosome focused refinement map, and H3-ARP6-ZNHIT1 focused refinement map show that the H3-ARP6 interaction is dynamic and unable to be fully separated within the fully engaged state particles. The composite map-model (bottom right) show the two potential positions of the N-terminal alpha-helix of H3 (bound and unbound).

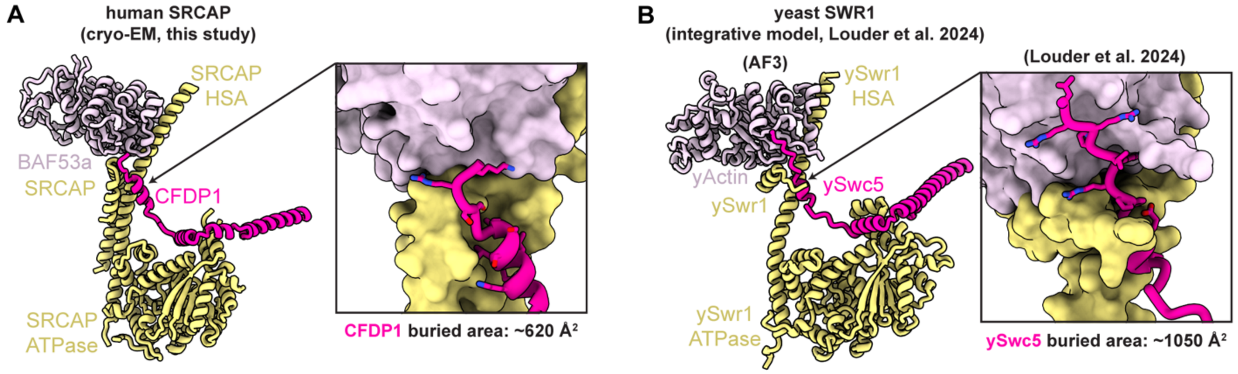

Fig. S42. Comparison of human CFPD1 and yeast Swc5 BCNT-I domain.
(A) Cryo-EM structure highlighting CFDP1 BCNT-I domain interaction with the SRCAP 'trident' submodule consisting of the SRCAP HSA (helicase-SANT-Associated) helix, SRCAP C-terminal region, and BAF53a. The inset shows a zoom in of BCNT-I domain with SRCAP and BAF53a represented as surfaces. (B) An integrative model highlighting ySwc5 BCNT-I domain interaction with the ySwr1 submodule consisting of the ySwr1 HSA (helicase-SANT-Associated) helix, ySwr1 C-terminal region, and yActin. The inset shows a zoomin of BCNT-I domain with ySwr1 and yActin represented as surfaces.

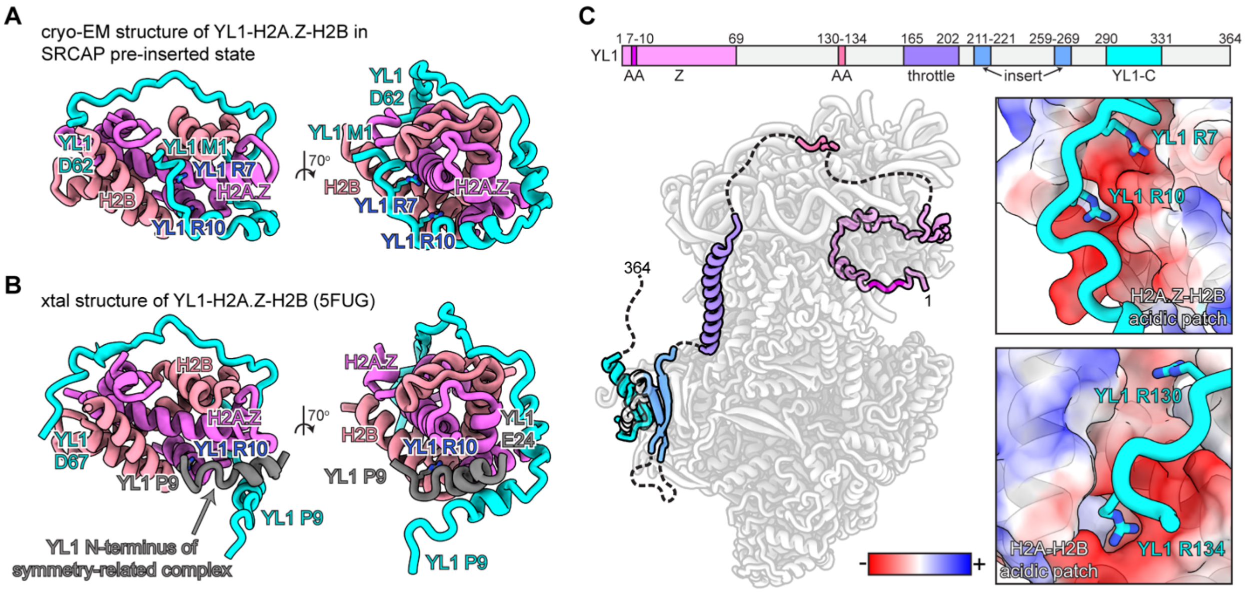

Fig. S43. Structural details of YL1.
(A) Cryo-EM structure of H2A.Z-H2B-YL1 in the preinserted state. Here, the R7 and R10 arginine anchors of YL1 interact with the acidic patch of the H2A.Z-H2B dimer. (B) X-ray crystal structure of H2A.Z-H2B-YL1 in isolation (PDB: 5FUG). Crystallization induced dimerization of YL1 residues 16-24 lead to R10 of YL1 interacting with a distinct symmetry related H2A.Z-H2B complex. (C) Domain map (top) of YL1 and structure of YL1 in the fully inserted state with important domains colored. YL1 contains a total of four arginine anchors (R7, R10, R130, R134) which interact with the H2A.Z-H2B and H2A-H2B acidic patch. The acidic patches are represented as a surface and colored according to Coulombic electrostatic potential (red: -10 kcal/(mol·e), blue: +10 kcal/(mol·e)).

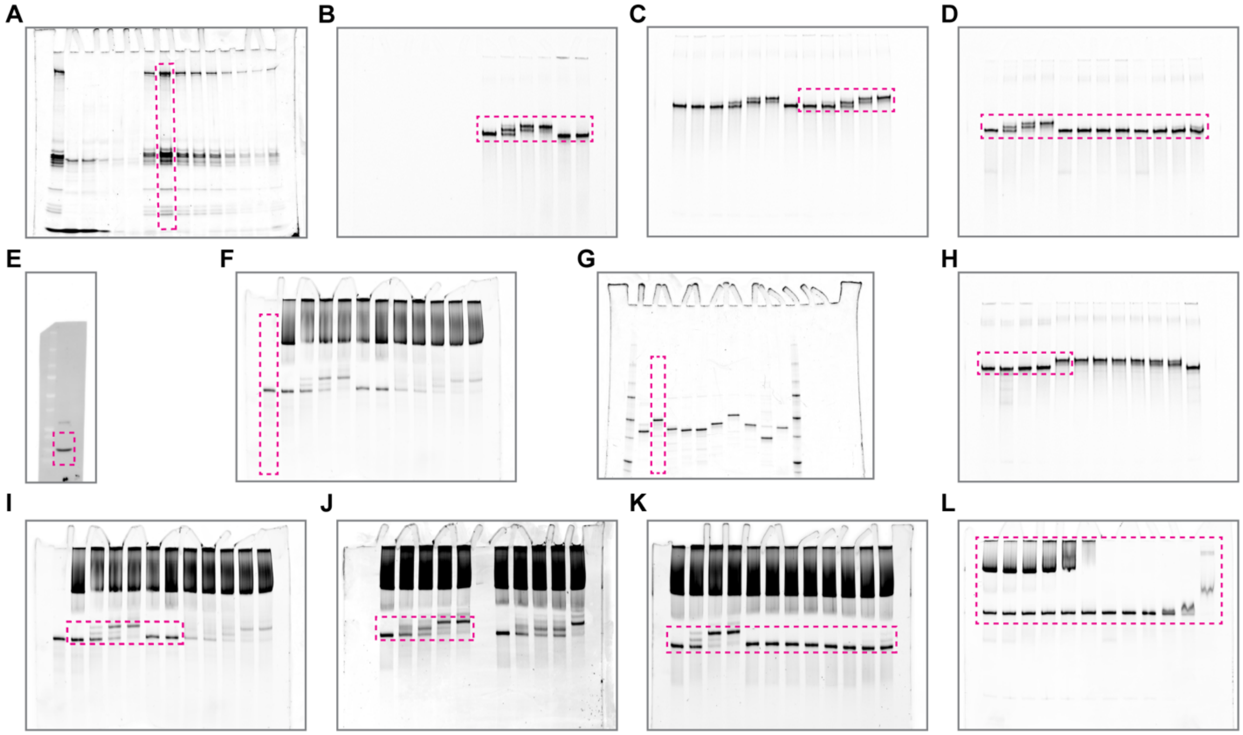

Fig. S44. Uncropped gels.

(A) Uncropped gel associated with Fig. 1B. The cropped region is indicated by the dashed box.
(B) Uncropped gel associated with Fig. 1D. The cropped region is indicated by the dashed box.
(C) Uncropped gel associated with Fig. 1E. The cropped region is indicated by the dashed box.
(D) Uncropped gel associated with Fig. 3C. The cropped region is indicated by the dashed box.
(E) Uncropped gel associated with fig. S2B. The cropped region is indicated by the dashed box.
(F) Uncropped gel associated with fig. S2C. The cropped region is indicated by the dashed box.
(G) Uncropped gel associated with fig. S2D. The cropped region is indicated by the dashed box.
(H) Uncropped gel associated with fig. S2E. The cropped region is indicated by the dashed box.
(I) Uncropped gel associated with fig. S2F. The cropped region is indicated by the dashed box.
(J) Uncropped gel associated with fig. S2G. The cropped region is indicated by the dashed box.
(K) Uncropped gel associated with fig. S2H. The cropped region is indicated by the dashed box.
(L) Uncropped gel associated with fig. S3A. The cropped region is indicated by the dashed box.

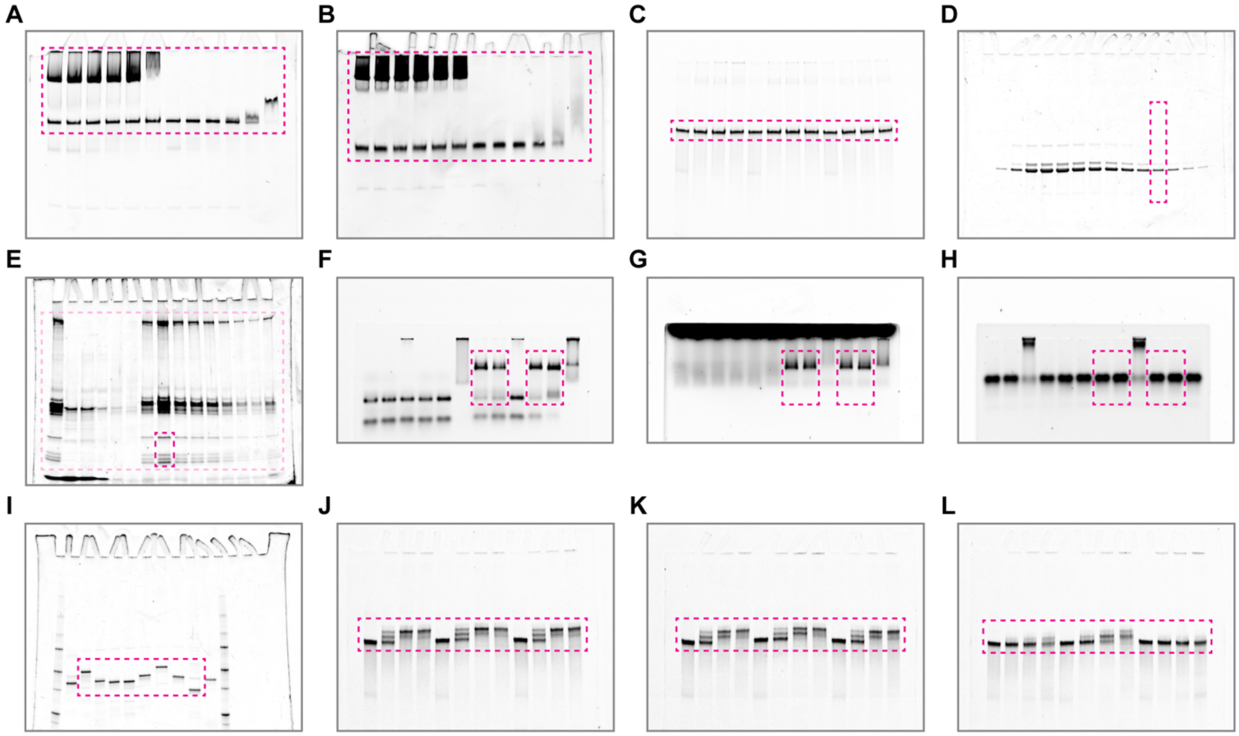

Fig. S45. Uncropped gels (continued).
(A) Uncropped gel associated with fig. S3B. The cropped region is indicated by the dashed box. (B) Uncropped gel associated with fig. S3C. The cropped region is indicated by the dashed box. (C) Uncropped gel associated with fig. S3D. The cropped region is indicated by the dashed box. (D) Uncropped gel associated with fig. S3E. The cropped region is indicated by the dashed box. (E) Uncropped gel associated with fig. S3F. The cropped region is indicated by the dashed box. (F-H) Uncropped gels associated with fig. S3G. The cropped regions are indicated by the dashed box. (I) Uncropped gel associated with fig. S27H. The cropped region is indicated by the dashed box. (J-L) Uncropped gels associated with fig. S27I. The cropped regions are indicated by the dashed box.

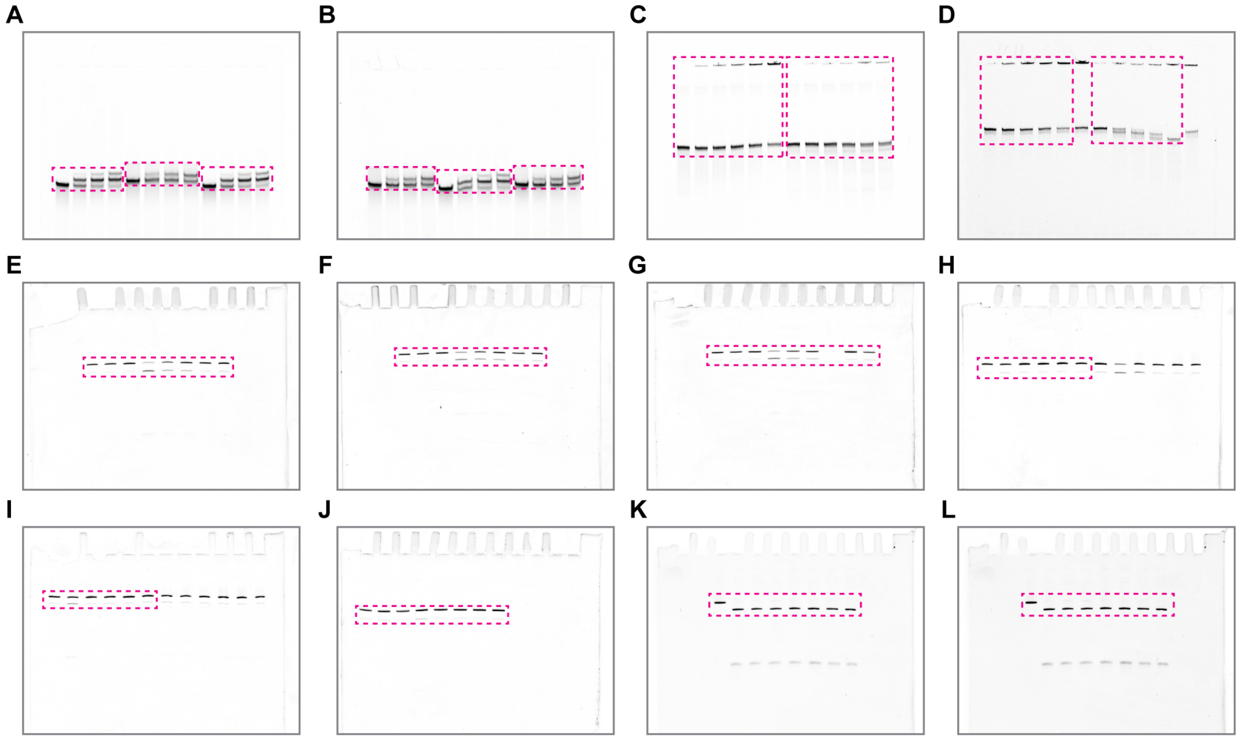

Fig. S46. Uncropped gels (continued).
(A-B) Uncropped gels associated with fig. S31C. The cropped regions are indicated by the dashed box. (C) Uncropped gel associated with fig. S37D. The cropped region is indicated by the dashed box. (D) Uncropped gel associated with fig. S37E. The cropped region is indicated by the dashed box. (E-L) Uncropped gels associated with fig. S36A-H. The cropped regions are indicated by the dashed box.

Table S1. Cryo-EM data collection, refinement and validation statistics.

	SRCAP (unbound) EMD-45383 PDB 9CA9	SRCAP-nucleosome, preengaged EMD-45384 PDB 9CAA	SRCAP-nucleosome, partially engaged EMD-45382 PDB 9CA8	SRCAP-nucleosome, fully engaged EMD-45381 PDB 9CA7
**Data collection and processing**				
Data set	Data set 1	Data set 1	Data set 1	Data set 1
Microscope	Krios G3i	Krios G3i	Krios G3i	Krios G3i
Voltage (kV)	300	300	300	300
Nominal magnification	22,500	22,500	22,500	22,500
Energy filter	none	none	none	none
Energy slit width				
Detector	K3	K3	K3	K3
Pixel size (Å)	1.025	1.025	1.025	1.025
Defocus range (μm)	-0.8 to -1.6	-0.8 to -1.6	-0.8 to -1.6	-0.8 to -1.6
Exposure time	4	4	4	4
Electron exposure (e–/Å^2)	50	50	50	50
Movies collected	20,709	20,709	20,709	20,709
**Cryo-EM Reconstruction**				
Software	RELION	RELION	RELION	RELION
Particle images (no.)	37,293	28,381	24,701	227,030
Consensus map resolution, FSC = 0.143 (Å)	3.6	4.0	3.9	3.4
**Coordinates Refinement**				
Software	PHENIX	PHENIX	PHENIX	PHENIX
Map vs. model resolution, FSC = 0.5 (Å)	3.2	3.9	3.4	2.6
Map vs. model cross-correlation	0.79	0.67	0.76	0.85
Model composition				
Chains	18	28	29	29
Non-hydrogen atoms	28,594	40,985	42,947	42,373
Protein residues	3,618	4,386	4,709	4,709
DNA residues	0	306	270	242
Ligands	AGS(1), ADP(6), Mg^{2+}(4), Zn^{2+}(2)	AGS(1), ADP(6), Mg^{2+}(5), Zn^{2+}(2)	AGS(2), ADP(6), Mg^{2+}(5), Zn^{2+}(2)	AGS(2), ADP(6), Mg^{2+}(5), Zn^{2+}(2)
Average *B* factors (Å^2)				
Protein	97	109	95	59
DNA	N/A	134	140	71
Ligand	72	88	88	48
R.m.s. deviations				
Bond lengths (Å)	0.003	0.002	0.003	0.004
Bond angles (°)	0.482	0.488	0.513	0.555
Validation				
MolProbity score	1.20	1.17	1.18	1.13
All-atom clashscore	4.12	3.86	3.91	3.38
Poor rotamers (%)	0.0	0.0	0.0	0.0
Ramachandran plot				
Favored (%)	98.0	98.1	98.2	98.1
Allowed (%)	2.0	1.9	1.8	1.9
Disallowed (%)	0.0	0.0	0.0	0.0

Table S2. Cryo-EM data collection, refinement and validation statistics.

	SRCAP-CFDP1-hexasome, evicted EMD-49376 PDB 9NFX	SRCAP-CFDP1-pre-nucleosome, preinserted EMD-49377 PDB 9NFY	SRCAP-nucleosome, fully inserted EMD-49378 PDB 9NFZ	SRCAP-nucleosome, predissociated EMD-49379 PDB 9NG0
**Data collection and processing**				
Data set	Data set 1	Data set 1	Data set 1	Data set 1
Microscope	Krios G3i	Krios G3i	Krios G3i	Krios G3i
Voltage (kV)	300	300	300	300
Nominal magnification	22,500	22,500	22,500	22,500
Energy filter	none	none	none	none
Energy slit width				
Detector	K3	K3	K3	K3
Pixel size (Å)	1.025	1.025	1.025	1.025
Defocus range (µm)	-0.8 to -1.6	-0.8 to -1.6	-0.8 to -1.6	-0.8 to -1.6
Exposure time	4	4	4	4
Electron exposure (e–/Å^2)	50	50	50	50
Movies collected	20,709	20,709	20,709	20,709
**Cryo-EM Reconstruction**				
Software	RELION	RELION	RELION	RELION
Particle images (no.)	43,233	43,233	24,589	24,877
Consensus map resolution, FSC = 0.143 (Å)	3.3	3.4	3.8	3.9
**Coordinates Refinement**				
Software	PHENIX	PHENIX	PHENIX	PHENIX
Map vs. model resolution, FSC = 0.5 (Å)	3	3.4	3.4	3.4
Map vs. model cross-correlation	0.81	0.8	0.76	0.75
Model composition				
Chains	28	30	29	29
Non-hydrogen atoms	40,354	42,380	42,020	41,804
Protein residues	4,515	4,779	4,723	4,698
DNA residues	216	216	222	220
Ligands	AGS(2), ADP(6), Mg^{2+}(5), Zn^{2+}(2)	AGS(2), ADP(6), Mg^{2+}(5), Zn^{2+}(2)	AGS(1), ADP(6), Mg^{2+}(4), Zn^{2+}(2)	AGS(1), ADP(6), Mg^{2+}(4), Zn^{2+}(2)
Average *B* factors (Å^2)				
Protein	77	79	88	89
DNA	120	115	114	118
Ligand	65	62	83	82
R.m.s. deviations				
Bond lengths (Å)	0.003	0.003	0.003	0.003
Bond angles (°)	0.589	0.553	0.550	0.554
Validation				
MolProbity score	1.20	1.18	1.11	1.12
All-atom clashscore	4.12	3.91	3.22	3.31
Poor rotamers (%)	0.0	0.0	0.0	0.0
Ramachandran plot				
Favored (%)	98.3	98.3	98.2	98.2
Allowed (%)	1.7	1.7	1.8	1.8
Disallowed (%)	0.0	0.0	0.0	0.0

Table S3. Cryo-EM data collection, refinement and validation statistics.

	SRCAP-CFDP1-nucleosome, poised EMD-49374 PDB 9NFV	SRCAP-CFDP1-nucleosome, activated EMD-49375 PDB 9NFW
**Data collection and processing**		
Data set	Data set 2	Data set 2
Microscope	Krios G3i	Krios G3i
Voltage (kV)	300	300
Nominal magnification	22,500	22,500
Energy filter	none	none
Energy slit width		
Detector	K3	K3
Pixel size (Å)	1.025	1.025
Defocus range (μm)	-0.8 to -1.6	-0.8 to -1.6
Exposure time	4.0	4.0
Electron exposure (e–/Å^2)	50	50
Movies collected	9,161	9,161
**Cryo-EM Reconstruction**		
Software	RELION	RELION
Particle images (no.)	22,651	37,606
Consensus map resolution, FSC = 0.143 (Å)	3.8	3.8
**Coordinates Refinement**		
Software	PHENIX	PHENIX
Map vs. model resolution, FSC = 0.5 (Å)	3.6	3.6
Map vs. model cross-correlation	0.75	0.75
Model composition		
Chains	30	30
Non-hydrogen atoms	43,233	43,402
Protein residues	4,787	4,801
DNA residues	252	254
Ligands	ATP(2), ADP(6), Mg^{2+}(5), Zn^{2+}(2)	ATP(2), ADP(6), Mg^{2+}(5), Zn^{2+}(2)
Average *B* factors (Å^2)		
Protein	101	101
DNA	164	167
Ligand	88	93
R.m.s. deviations		
Bond lengths (Å)	0.003	0.003
Bond angles (°)	0.582	0.542
Validation		
MolProbity score	1.13	1.11
All-atom clashscore	3.39	3.21
Poor rotamers (%)	0.0	0.0
Ramachandran plot		
Favored (%)	98.2	98.2
Allowed (%)	1.8	1.8
Disallowed (%)	0.0	0.0

Table S4. Cryo-EM data collection, refinement and validation statistics.

	SRCAP-nucleosome, preengaged [extended model] EMD-72455 PDB 9Y3E	SRCAP-nucleosome, fully engaged [extended model] EMD-71627 PDB 9Y3D	SRCAP-CFDP1-nucleosome, poised [extended model] EMD-72456 PDB 9Y3F	SRCAP-CFDP1-nucleosome, activated [extended model] EMD-72457 PDB 9Y3G	SRCAP-CFDP1-nucleosome, unwrapping [extended model] EMD-72458 PDB 9Y3H
**Data collection and processing**					
Data set	Data set 1	Data set 1	Data set 2	Data set 2	Data set 2
Microscope	Krios G3i	Krios G3i	Krios G3i	Krios G3i	Krios G3i
Voltage (kV)	300	300	300	300	300
Nominal magnification	22,500	22,500	22,500	22,500	22,500
Energy filter	none	none	none	none	none
Energy slit width					
Detector	K3	K3	K3	K3	K3
Pixel size (Å)	1.025	1.025	1.025	1.025	1.025
Defocus range (μm)	-0.8 to -1.6	-0.8 to -1.6	-0.8 to -1.6	-0.8 to -1.6	-0.8 to -1.6
Exposure time	4	4	4	4	4
Electron exposure (e–/Å^2)	50	50	50	50	50
Movies collected	20,709	20,709	9,161	9,161	9,161
**Cryo-EM Reconstruction**					
Software	RELION	RELION	RELION	RELION	RELION
Particle images (no.)	23,381	5,605	22,651	37,606	22,651
Consensus map resolution, FSC = 0.143 (Å)	5.0	7.1	4.5	4.1	4.5
**Coordinates Refinement**					
Software	PHENIX	PHENIX	PHENIX	PHENIX	PHENIX
Map vs. model resolution, FSC = 0.5 (Å)	6.8	7.5	4.4	4.2	8.8
Map vs. model cross-correlation	0.56	0.76	0.66	0.62	0.66
Model composition					
Chains	33	35	35	35	34
Non-hydrogen atoms	56,261	59,547	55,482	55,610	55,696
Protein residues	6,221	6,594	6,182	6,196	6,182
DNA residues	330	342	310	310	322
Ligands	AGS(2), ADP(6), Mg^{2+}(5), Zn^{2+}(2)	AGS(3), ADP(6), Mg^{2+}(6), Zn^{2+}(2)	ATP(3), ADP(6), Mg^{2+}(6), Zn^{2+}(2)	ATP(3), ADP(6), Mg^{2+}(6), Zn^{2+}(2)	ATP(2), ADP(6), Mg^{2+}(5), Zn^{2+}(2)
Average *B* factors (Å^2)					
Protein	449	456	318	341	455
DNA	516	587	517	560	794
Ligand	163	369	230	267	346
R.m.s. deviations					
Bond lengths (Å)	0.002	0.002	0.002	0.002	0.002
Bond angles (°)	0.526	0.536	0.545	0.540	0.558
Validation					
MolProbity score	1.13	1.13	1.13	1.07	1.09
All-atom clashscore	3.35	3.40	3.34	2.86	3.02
Poor rotamers (%)	0.0	0.0	0.0	0.0	0.0
Ramachandran plot					
Favored (%)	98.3	98.4	98.3	98.4	98.3
Allowed (%)	1.7	1.6	1.7	1.6	1.7
Disallowed (%)	0.0	0.0	0.0	0.0	0.0

Table S5. Cryo-EM data collection, refinement and validation statistics.

	**ARP6-ZnHIT submodule from SRCAP-nucleosome, fully engaged state with H3-bound ARP6 EMD-71626 PDB 9PGD**	**Trident submodule from SRCAP-nucleosome fully engaged state EMD-71625 PDB 9PGC**	**Unbound 106-N-32 nucleosome EMD-74396 PDB 9ZLA**
**Data collection and processing**			
Data set	Data set 1	Data set 1	Data set 1
Microscope	Krios G3i	Krios G3i	Krios G3i
Voltage (kV)	300	300	300
Nominal magnification	22,500	22,500	22,500
Energy filter	none	none	none
Energy slit width			
Detector	K3	K3	K3
Pixel size (Å)	1.025	1.025	1.025
Defocus range (μm)	-0.8 to -1.6	-0.8 to -1.6	-0.8 to -1.6
Exposure time	4	4	4
Electron exposure (e–/Å^2)	50	50	50
Movies collected	20,709	20,709	20,709
**Cryo-EM Reconstruction**			
Software	RELION	RELION	RELION
Particle images (no.)	227,030	57,610	93,703
Consensus map resolution, FSC = 0.143 (Å)	3.2	3.8	3.1
**Coordinates Refinement**			
Software	PHENIX	PHENIX	PHENIX
Map vs. model resolution, FSC = 0.5 (Å)	3.1	3.8	3
Map vs. model cross-correlation	0.86	0.63	0.80
Model composition			
Chains	5	7	10
Non-hydrogen atoms	4,251	9,184	12,443
Protein residues	519	1,059	774
DNA residues	0	26	306
Ligands	AGS(1), Mg^{2+}(1), Zn^{2+}(2)	AGS(2), Mg^{2+}(2)	N/A
Average *B* factors (Å^2)			
Protein	77	119	39
DNA	N/A	87	57
Ligand	61	116	N/A
R.m.s. deviations			
Bond lengths (Å)	0.006	0.004	0.003
Bond angles (°)	0.597	0.572	0.536
Validation			
MolProbity score	1.42	1.22	1.14

Table S6. Cryo-EM data associated with main figure panels.

Figure	SRCAP state	EMDB/PDB	Dataset-state
1F	Free	45383/9CA9	1-1
1F	Preengaged	45384/9CAA	1-2
1F	Fully engaged	45381/9CA7	1-4
1F	Poised	49374/9NFV	5-1
1F	Activated	49375/9NFW	5-2
1F	Unwrapping	72458/9Y3H	5-3
1F	Evicted	49376/9NFX	1-6
1F	Preinserted	49377/9NFY	1-7
1F	Fully inserted	49378/9NFZ	1-8
2A	Preengaged	45384/9CAA, 72455/9Y3E	1-2
2A	Fully engaged	45381/9CA7, 71625/9PGC	1-4
2B	Preengaged	45384/9CAA	1-2
2B	Fully engaged	45381/9CA7	1-4
2C	Preengaged	45384/9CAA	1-2
2C	Fully engaged	45381/9CA7	1-4
2D	Fully engaged	45381/9CA7, 71625/9PGC, 71627/9Y3D	1-4
2E	Fully engaged	71625/9PGC	1-4
2F	Fully engaged	71627/9Y3D	1-4
2G	Fully engaged	45381/9CA7, 71625/9PGC, 71627/9Y3D	1-4
2H	Fully engaged	45381/9CA7, 71625/9PGC, 71627/9Y3D	1-4
2I	Fully engaged	74291	1-4
3A	Poised	49374/9NFV, 72456/9Y3F	5-1
3D	Poised	49374/9NFV	5-1
3E	Poised	49374/9NFV, 72456/9Y3F	5-1
3F	Fully engaged	71625/9PGC	1-4
3F	Poised	49374/9NFV	5-1
3F	Activated	49375/9NFW	5-2
3G	Poised	49374/9NFV	5-1
3G	Activated	49375/9NFW	5-2
3H	Activated	49375/9NFW	5-2
3I	Activated	49375/9NFW, 72457/9Y3G	5-2
4A	Evicted	49376/9NFX	1-6
4B	Evicted	49376/9NFX, 49434	1-6
4C	Evicted	49376/9NFX	1-6
4D	Evicted	49376/9NFX	1-6
4E	Nucleosome	74396/9ZLA	
4E	Poised	72456/9Y3F	5-1
4E	Activated	72457/9Y3G	5-2
4E	Unwrapping	72458/9Y3H	5-3
4E	Evicted	49434	1-6
4F	Activated	49375/9NFW	5-2
4F	Evicted	49376/9NFX	1-6
4G	Partially engaged	45382/9CA8	1-3
4G	Fully engaged	45381/9CA7, 71626/9PGD	1-4
4G	Activated	49375/9NFW	5-2
4G	Evicted	49376/9NFX	1-6
5A	Fully engaged	45381/9CA7, 74291	1-4
5A	Evicted	49376/9NFX	1-6
5A	Preinserted	49377/9NFY	1-7
5A	Fully inserted	49378/9NFZ	1-8
5B	Fully engaged	45381/9CA7	1-4
5C	Preinserted	49377/9NFY	1-7
5D	Preinserted	49377/9NFY	1-7
5D	Fully inserted	49378/9NFZ	1-8
5E	Preinserted	49377/9NFY, 49439	1-7
6A	Evicted	74500	12-1
6A	Evicted	49376/9NFX	1-6
6A	Evicted	74476	11-1
6A	Evicted	74463	9-1
6A	Evicted	74464	10-1
6C	Activated	49375/9NFW, 72457/9Y3G	5-2
6C	Evicted	49376/9NFX, 49434	1-6

Table S7. Primary cryo-EM maps and models deposited.

PDB / EMDB codes	PDB entry title	Dataset-state
9CA9 / EMD-45383	Cryo-EM structure of the human SRCAP complex in the unbound state (composite structure)	1-1
9CAA / EMD-45384	Cryo-EM structure of human SRCAP-nucleosome complex in the pre-engaged state (composite structure)	1-2
9CA8 / EMD-45382	Cryo-EM structure of human SRCAP-nucleosome complex in the partially-engaged state (composite structure)	1-3
9CA7 / EMD-45381	Cryo-EM structure of human SRCAP-nucleosome complex in the fully-engaged state (composite structure)	1-4
9NFV / EMD-49374	Human SRCAP-CFDP1-nucleosome complex in the poised state of the H2A.Z histone exchange reaction (composite structure)	5-1
9NFW / EMD-49375	Human SRCAP-CFDP1-nucleosome complex in the activated state of the H2A.Z histone exchange reaction (composite structure)	5-2
9NFX / EMD-49376	Human SRCAP-CFDP1-hexasome complex in the evicted state of the H2A.Z histone exchange reaction (composite structure)	1-6
9NFY / EMD-49377	Human SRCAP-CFDP1-prenucleosome complex in the pre-inserted state of the H2A.Z histone exchange reaction (composite structure)	1-7
9NFZ / EMD-49378	Human SRCAP-nucleosome complex in the fully inserted state of the H2A.Z histone exchange reaction (composite structure)	1-8
9NG0 / EMD-49379	Human SRCAP-nucleosome complex in the predissociation state of the H2A.Z histone exchange reaction (composite structure)	1-9
9Y3E / EMD-72455	Extended cryo-EM structure of human SRCAP-nucleosome complex in the pre-engaged state	1-2
9Y3D / EMD-71627	Extended cryo-EM structure of the human SRCAP-nucleosome complex in the fully-engaged state, with H4-bound GAS41	1-4
9Y3F / EMD-72456	Extended cryo-EM structure of human SRCAP-CFDP1-nucleosome complex in the poised state	5-1
9Y3G / EMD-72457	Extended cryo-EM structure of human SRCAP-CFDP1-nucleosome complex in the activated state	5-2
9Y3H / EMD-72458	Human SRCAP-CFDP1-nucleosome complex in the unwrapping state of the H2A.Z histone exchange reaction	5-3
9PGC / EMD-71625	Trident submodule from human SRCAP-nucleosome complex in the fully-engaged state (focused refinement)	1-4
9PGD / EMD-71626	ARP6-ZNHIT1 module from fully-engaged state of SRCAP-nucleosome complex, with H3-bound ARP6 (focused refinement, filtered by local resolution)	1-4
9ZLA / EMD-74396	Unbound 106-N-32 nucleosome from SRCAP-CFDP1-nucleosome binding reaction	1-0

Table S8. Supplementary cryo-EM maps deposited.

EMDB code	EMDB entry title	Dataset-state
EMD-49786	ARP6-ZNHIT1 module from unbound SRCAP complex (focused refinement)	1-1
EMD-49785	RuvBL core from unbound SRCAP complex (focused refinement)	1-1
EMD-49784	Cryo-EM structure of human SRCAP complex in the unbound state (consensus map filtered by local resolution)	1-1
EMD-49780	Cryo-EM structure of human SRCAP-nucleosome complex in the pre-engaged state (consensus map filtered by local resolution)	1-2
EMD-49781	RuvBL core from pre-engaged state of SRCAP-nucleosome complex (focused refinement)	1-2
EMD-49782	ARP6-ZNHIT1 module from pre-engaged state of SRCAP-nucleosome complex (focused refinement)	1-2
EMD-49783	Nucleosome from pre-engaged state of SRCAP-nucleosome complex (focused refinement)	1-2
EMD-49775	Cryo-EM structure of human SRCAP-nucleosome complex in the partially-engaged state (consensus map filtered by local resolution)	1-3
EMD-49776	RuvBL core from partially-engaged state of SRCAP-nucleosome complex (focused refinement)	1-3
EMD-49777	ARP6-ZNHIT1 module from partially-engaged state of SRCAP-nucleosome complex (focused refinement)	1-3
EMD-49779	ATPase module from partially-engaged state of SRCAP-nucleosome complex (focused refinement)	1-3
EMD-49778	Nucleosome from partially-engaged state of SRCAP-nucleosome complex (focused refinement)	1-3
EMD-49770	Cryo-EM structure of human SRCAP-nucleosome complex in the fully-engaged state (consensus map filtered by local resolution)	1-4
EMD-49771	RuvBL core from fully-engaged state of SRCAP-nucleosome complex (focused refinement)	1-4
EMD-49772	ARP6-ZNHIT1 module from fully-engaged state of SRCAP-nucleosome complex (focused refinement)	1-4
EMD-49773	ATPase module from fully-engaged state of SRCAP-nucleosome complex (focused refinement)	1-4
EMD-49774	Nucleosome from fully-engaged state of SRCAP-nucleosome complex (focused refinement)	1-4
EMD-71628	Human SRCAP-nucleosome complex in the fully-engaged state, with unbound GAS41 (unmasked refinement, filtered by local resolution)	1-4
EMD-71627	Human SRCAP-nucleosome complex in the fully-engaged state, with H4-bound GAS41 (unmasked refinement, filtered by local resolution)	1-4
EMD-74291	Human SRCAP-nucleosome complex in the fully-engaged state, subclass with density for sequestered H2A.Z-H2B (unmasked refinement, filtered by local resolution)	1-4
EMD-49423	Human SRCAP-CFDP1-nucleosome complex in the poised state of the H2A.Z histone exchange reaction (consensus map filtered by local resolution)	5-1
EMD-49424	RuvBL core from poised state of SRCAP-CFDP1-nucleosome complex (focused refinement)	5-1
EMD-49425	Arp6-ZnHIT1 module from poised state of SRCAP-CFDP1-nucleosome complex (focused refinement)	5-1
EMD-49426	ATPase module from poised state of SRCAP-CFDP1-nucleosome complex (focused refinement)	5-1
EMD-49427	Nucleosome from poised state of SRCAP-CFDP1-nucleosome complex (focused refinement)	5-1
EMD-74416	Trident submodule from human SRCAP-CFDP1-nucleosome complex in the poised state (focused refinement)	5-1
EMD-49428	Human SRCAP-CFDP1-nucleosome complex in the activated state of the H2A.Z histone exchange reaction (consensus map filtered by local resolution)	5-2
EMD-49429	RuvBL core from activated state of SRCAP-CFDP1-nucleosome complex (focused refinement)	5-2
EMD-49430	Arp6-ZnHIT1 module from activated state of SRCAP-CFDP1-nucleosome complex (focused refinement)	5-2
EMD-49431	ATPase module from activated state of SRCAP-CFDP1-nucleosome complex (focused refinement)	5-2
EMD-49432	Nucleosome from activated state of SRCAP-CFDP1-nucleosome complex (focused refinement)	5-2
EMD-49434	Human SRCAP-CFDP1-hexasome complex in the evicted state of the H2A.Z histone exchange reaction (consensus map filtered by local resolution)	1-6,1-7
EMD-49435	RuvBL core from evicted state of SRCAP-CFDP1-hexasome complex (focused refinement)	1-6,1-7
EMD-49436	Arp6-ZnHIT1 module from evicted state of SRCAP-CFDP1-hexasome complex (focused refinement)	1-6,1-7
EMD-49437	ATPase module from evicted state of SRCAP-CFDP1-hexasome complex (focused refinement)	1-6,1-7
EMD-49438	Hexasome from evicted state of SRCAP-CFDP1-hexasome complex (focused refinement)	1-6
EMD-74393	ATPase module from evicted state of SRCAP-CFDP1-hexasome complex, subclass with density for CFDP1 FAGE domain (focused refinement)	1-6
EMD-49439	Prenucleosome from pre-inserted state of SRCAP-CFDP1-prenucleosome complex (focused refinement)	1-7
EMD-74337	Prenucleosome from pre-inserted state of SRCAP-CFDP1-prenucleosome complex, subclass with alternative dimer position B (focused refinement)	1-7
EMD-74338	Prenucleosome from pre-inserted state of SRCAP-CFDP1-prenucleosome complex, subclass with alternative dimer position C (focused refinement)	1-7
EMD-49440	Human SRCAP-nucleosome complex in the fully inserted state of the H2A.Z histone exchange reaction (consensus map filtered by local resolution)	1-8
EMD-49441	RuvBL core from fully inserted state of SRCAP-nucleosome complex (focused refinement)	1-8
EMD-49442	Arp6-ZnHIT1 module from fully inserted state of SRCAP-nucleosome complex (focused refinement)	1-8
EMD-49443	ATPase module from fully inserted state of SRCAP-nucleosome complex (focused refinement)	1-8
EMD-49444	Nucleosome from fully inserted state of SRCAP-nucleosome complex (focused refinement)	1-8
EMD-49445	Human SRCAP-nucleosome complex in the pre-dissociation state of the H2A.Z histone exchange (consensus map filtered by local resolution)	1-9
EMD-49446	RuvBL core from pre-dissociation state of SRCAP-nucleosome complex (focused refinement)	1-9
EMD-49447	Arp6-ZnHIT1 module from pre-dissociation state of SRCAP-nucleosome complex (focused refinement)	1-9
EMD-49448	ATPase module from pre-dissociation state of SRCAP-nucleosome complex (focused refinement)	1-9
EMD-49449	Nucleosome from pre-dissociation state of SRCAP-nucleosome complex (focused refinement)	1-9
EMD-74463	Evicted state of the human SRCAP-CFDP1-hexasome complex formed in the presence of ADP-BeFx (consensus map filtered by local resolution)	9-1
EMD-74464	Evicted state of the human SRCAP-CFDP1-hexasome complex formed in the presence of ADP-Vi (consensus map filtered by local resolution)	10-1
EMD-74476	Evicted state of the human SRCAP-CFDP1-hexasome complex formed in the presence of AMP-PNP (composite structure)	11-1
EMD-74500	Evicted state of the human SRCAP-CFDP1-hexasome complex formed in the presence of ATP (composite structure)	12-1

Table S8. (continued) Supplementary cryo-EM maps deposited.

EMDB code	EMDB entry title	Dataset-state
EMD-74556	Evicted state of the human SRCAP-CFDP1-hexasome complex formed in the presence of ATP (consensus map filtered by local resolution)	12-1
EMD-74557	RuvBL core from evicted state of SRCAP-CFDP1-hexasome complex formed in the presence of ATP (focused refinement)	12-1
EMD-74558	Arp6-ZnHIT1 module from evicted state of SRCAP-CFDP1-hexasome complex formed in the presence of ATP (focused refinement)	12-1
EMD-74559	ATPase module from evicted state of SRCAP-CFDP1-hexasome complex formed in the presence of ATP (focused refinement)	12-1
EMD-74560	Hexasome from evicted state of SRCAP-CFDP1-hexasome complex formed in the presence of ATP (focused refinement)	
EMD-74551	Evicted state of the human SRCAP-CFDP1-hexasome complex formed in the presence of AMP-PNP (consensus map filtered by local resolution)	11-1
EMD-74552	RuvBL core from evicted state of SRCAP-CFDP1-hexasome complex formed in the presence of AMP-PNP (focused refinement)	11-1
EMD-74553	Arp6-ZnHIT1 module from evicted state of SRCAP-CFDP1-hexasome complex formed in the presence of AMP-PNP (focused refinement)	11-1
EMD-74554	ATPase module from evicted state of SRCAP-CFDP1-hexasome complex formed in the presence of AMP-PNP (focused refinement)	11-1
EMD-74555	Hexasome from evicted state of SRCAP-CFDP1-hexasome complex formed in the presence of AMP-PNP (focused refinement)	11-1
EMD-74624	Activated state of the human SRCAP-CFDP1-nucleosome complex formed in the presence of ATP-gamma-S (composite map)	1-5
EMD-74619	Activated state of the human SRCAP-CFDP1-nucleosome complex formed in the presence of ATP-gamma-S (consensus map filtered by local resolution)	1-5
EMD-74620	RuvBL core from activated state of SRCAP-CFDP1-nucleosome complex formed in the presence of ATP-gamma-S (focused refinement)	1-5
EMD-74621	Arp6-ZnHIT1 module from activated state of SRCAP-CFDP1-nucleosome complex formed in the presence of ATP-gamma-S (focused refinement)	1-5
EMD-74622	ATPase module from activated state of SRCAP-CFDP1-nucleosome complex formed in the presence of ATP-gamma-S (focused refinement)	1-5
EMD-74623	Nucleosome from activated state of SRCAP-CFDP1-nucleosome complex formed in the presence of ATP-gamma-S (focused refinement)	1-5
EMD-74644	Fully-engaged state of the human SRCAP-nucleosome complex formed in the presence of ATP-gamma-S and absence of CFDP1 (composite map)	3-1
EMD-74639	Fully-engaged state of the human SRCAP-nucleosome complex formed in the presence of ATP-gamma-S and absence of CFDP1 (consensus map filtered by local resolution)	3-1
EMD-74640	RuvBL core from fully-engaged state of the human SRCAP-nucleosome complex formed in the presence of ATP-gamma-S and absence of CFDP1 (focused refinement)	3-1
EMD-74641	ARP6-ZNHIT1 module from fully-engaged state of the human SRCAP-nucleosome complex formed in the presence of ATP-gamma-S and absence of CFDP1 (focused refinement)	3-1
EMD-74642	ATPase module from fully-engaged state of the human SRCAP-nucleosome complex formed in the presence of ATP-gamma-S and absence of CFDP1 (focused refinement)	3-1
EMD-74643	Nucleosome from fully-engaged state of the human SRCAP-nucleosome complex formed in the presence of ATP-gamma-S and absence of CFDP1 (focused refinement)	3-1
EMD-74670	Fully-engaged state of the human SRCAP-nucleosome complex formed in the presence of AMP-PNP and absence of CFDP1 (composite map)	4-1
EMD-74664	Fully-engaged state of the human SRCAP-nucleosome complex formed in the presence of AMP-PNP and absence of CFDP1 (consensus map filtered by local resolution)	4-1
EMD-74665	RuvBL core from fully-engaged state of the human SRCAP-nucleosome complex formed in the presence of AMP-PNP and absence of CFDP1 (focused refinement)	4-1
EMD-74666	ARP6-ZNHIT1 module from fully-engaged state of the human SRCAP-nucleosome complex formed in the presence of AMP-PNP and absence of CFDP1 (focused refinement)	4-1
EMD-74667	ATPase module from fully-engaged state of the human SRCAP-nucleosome complex formed in the presence of AMP-PNP and absence of CFDP1 (focused refinement)	4-1
EMD-74668	Nucleosome from fully-engaged state of the human SRCAP-nucleosome complex formed in the presence of AMP-PNP and absence of CFDP1 (focused refinement)	4-1
EMD-74685	Poised state of the human SRCAP-CFDP1(delta284-299)-nucleosome mutant complex formed in the presence of ATP-gamma-S (composite map)	8-1
EMD-74678	Poised state of the human SRCAP-CFDP1(delta284-299)-nucleosome mutant complex formed in the presence of ATP-gamma-S (consensus map filtered by local resolution)	8-1
EMD-74680	RUVBL core from poised state of the human SRCAP-CFDP1(delta284-299)-nucleosome mutant complex formed in the presence of ATP-gamma-S (focused refinement)	8-1
EMD-74681	ARP6-ZNHIT1 module from poised state of the human SRCAP-CFDP1(delta284-299)-nucleosome mutant complex formed in the presence of ATP-gamma-S (focused refinement)	8-1
EMD-74683	ATPase module from poised state of the human SRCAP-CFDP1(delta284-299)-nucleosome mutant complex formed in the presence of ATP-gamma-S (focused refinement)	8-1
EMD-74684	Nucleosome from poised state of the human SRCAP-CFDP1(delta284-299)-nucleosome mutant complex formed in the presence of ATP-gamma-S (focused refinement)	8-1
EMD-74698	Fully inserted state of the human SRCAP-nucleosome complex formed in the presence of ATP (composite map)	12-2
EMD-74692	Fully inserted state of the human SRCAP-nucleosome complex formed in the presence of ATP (consensus map filtered by local resolution)	12-2
EMD-74694	RUVBL core from fully inserted state of the human SRCAP-nucleosome complex formed in the presence of ATP (focused refinement)	12-2
EMD-74695	ARP6-ZNHIT1 module from fully inserted state of the human SRCAP-nucleosome complex formed in the presence of ATP (focused refinement)	12-2
EMD-74696	ATPase module from fully inserted state of the human SRCAP-nucleosome complex formed in the presence of ATP (focused refinement)	12-2
EMD-74697	Nucleosome from fully inserted state of the human SRCAP-nucleosome complex formed in the presence of ATP (focused refinement)	12-2
EMD-74736	Poised state of the human SRCAP-CFDP1-nucleosome complex formed in the presence of ADP (consensus map filtered by local resolution)	7-1
EMD-74735	Activated state of the human SRCAP-CFDP1-nucleosome complex formed in the presence of ADP (consensus map filtered by local resolution)	7-2

Movie S1.
Mechanism of nucleosome engagement by SRCAP.

Movie S2.
CFDP1 binding and conformational activation of the SRCAP-nucleosome complex.

Movie S3.
Coordinated nucleosome unwrapping and H2A-H2B dimer eviction by the SRCAP-CFDP1 holoenzyme.

Movie S4.
The YL1 subunit chaperones the H2A.Z-H2B dimer into the nucleosome in coordination with nucleosome unwrapping.

Movie S5.
Full H2A.Z-H2B insertion to reassemble histone octamer is linked to CFDP1 dissociation.

Movie S6.
Mechanism of H2A.Z exchange by SRCAP.

REFERENCES

1. W. K. M. Lai, B. F. Pugh, Understanding nucleosome dynamics and their links to gene expression and DNA replication. *Nat. Rev. Mol. Cell Biol.* **18**, 548–562 (2017).

2. M. H. Hauer, S. M. Gasser, Chromatin and nucleosome dynamics in DNA damage and repair. *Genes Dev.* **31**, 2204–2221 (2017).

3. G. Arents, R. W. Burlingame, B. C. Wang, W. E. Love, E. N. Moudrianakis, The nucleosomal core histone octamer at 3.1 A resolution: A tripartite protein assembly and a left-handed superhelix. *Proc. Natl. Acad. Sci. U.S.A.* **88**, 10148–10152 (1991).

4. K. Luger, A. W. Mäder, R. K. Richmond, D. F. Sargent, T. J. Richmond, Crystal structure of the nucleosome core particle at 2.8 Å resolution. *Nature* **389**, 251–260 (1997).

5. C. R. Clapier, J. Iwasa, B. R. Cairns, C. L. Peterson, Mechanisms of action and regulation of ATP-dependent chromatin-remodelling complexes. *Nat. Rev. Mol. Cell Biol.* **18**, 407–422 (2017).

6. S. Eustermann, A. B. Patel, K.-P. Hopfner, Y. He, P. Korber, Energy-driven genome regulation by ATP-dependent chromatin remodellers. *Nat. Rev. Mol. Cell Biol.* **25**, 309–332 (2024).

7. L. Yan, Z. Chen, A unifying mechanism of DNA translocation underlying chromatin remodeling. *Trends Biochem. Sci.* **45**, 217–227 (2020).

8. G. Mizuguchi, X. Shen, J. Landry, W.-H. Wu, S. Sen, C. Wu, ATP-driven exchange of histone H2AZ variant catalyzed by SWR1 chromatin remodeling complex. *Science* **303**, 343–348 (2004).

9. B. D. Giaimo, F. Ferrante, A. Herchenröther, S. B. Hake, T. Borggrefe, The histone variant H2A.Z in gene regulation. *Epigenetics Chromatin* **12**, 37 (2019).

10. S. Martire, L. A. Banaszynski, The roles of histone variants in fine-tuning chromatin organization and function. *Nat. Rev. Mol. Cell Biol.* **21**, 522–541 (2020).

11. F. G. Ghiraldini, D. Filipescu, E. Bernstein, Solid tumours hijack the histone variant network. *Nat. Rev. Cancer* **21**, 257–275 (2021).

12. Y. Colino-Sanguino, S. J. Clark, F. Valdes-Mora, The H2A.Z-nucleosome code in mammals: Emerging functions. *Trends Genet.* **38**, 273–289 (2022).

13. D. D. Ruhl, J. Jin, Y. Cai, S. Swanson, L. Florens, M. P. Washburn, R. C. Conaway, J. W. Conaway, J. C. Chrivia, Purification of a human SRCAP complex that remodels chromatin by incorporating the histone variant H2A.Z into nucleosomes. *Biochemistry* **45**, 5671–5677 (2006).

14. M. M. Wong, L. K. Cox, J. C. Chrivia, The Chromatin Remodeling Protein, SRCAP, Is Critical for Deposition of the Histone Variant H2A.Z at Promoters*. *J. Biol. Chem.* **282**, 26132–26139 (2007).

15. Y. Yang, L. Zhang, C. Xiong, J. Chen, L. Wang, Z. Wen, J. Yu, P. Chen, Y. Xu, J. Jin, Y. Cai, G. Li, HIRA complex presets transcriptional potential through coordinating depositions of the histone variants H3.3 and H2A.Z on the poised genes in mESCs. *Nucleic Acids Res.* **50**, 191–206 (2022).

16. A. Tollenaere, E. Ugur, S. D. Longa, C. Deluz, D. Assenheimer, J. C. M. Gebardt, H. Leonhardt, D. M. Suter, Mechanisms of gene regulation by SRCAP and H2A.Z. *Nat. Commun.* **17**, 3560 (2026).

17. J. Yu, F. Sui, F. Gu, W. Li, Z. Yu, Q. Wang, S. He, L. Wang, Y. Xu, Structural insights into histone exchange by human SRCAP complex. *Cell Discov.* **10**, 15 (2024).

18. W.-H. Wu, S. Alami, E. Luk, C.-H. Wu, S. Sen, G. Mizuguchi, D. Wei, C. Wu, Swc2 is a widely conserved H2AZ-binding module essential for ATP-dependent histone exchange. *Nat. Struct. Mol. Biol.* **12**, 1064–1071 (2005).

19. W.-H. Wu, C.-H. Wu, A. Ladurner, G. Mizuguchi, D. Wei, H. Xiao, E. Luk, A. Ranjan, C. Wu, N terminus of Swr1 binds to histone H2AZ and provides a platform for subunit assembly in the chromatin remodeling complex. *J. Biol. Chem.* **284**, 6200–6207 (2009).

20. L. Sun, E. Luk, Dual function of Swc5 in SWR remodeling ATPase activation and histone H2A eviction. *Nucleic Acids Res.* **45**, 9931–9946 (2017).

21. Y. Huang, L. Sun, L. Pierrakeas, L. Dai, L. Pan, E. Luk, Z. Zhou, Role of a DEF/Y motif in histone H2A-H2B recognition and nucleosome editing. *Proc. Natl. Acad. Sci. U.S.A.* **117**, 3543–3550 (2020).

22. T. Nobukuni, M. Kobayashi, A. Omori, S. Ichinose, T. Iwanaga, I. Takahashi, K. Hashimoto, S. Hattori, K. Kaibuchi, Y. Miyata, T. Masui, S. Iwashita, An Alu-linked repetitive sequence corresponding to 280 amino acids is expressed in a novel bovine protein, but not in its human homologue. *J. Biol. Chem.* **272**, 2801–2807 (1997).

23. T. G. Diekwisch, F. Marches, A. Williams, X. Luan, Cloning, gene expression, and characterization of CP27, a novel gene in mouse embryogenesis. *Gene* **235**, 19–30 (1999).

24. G. Messina, E. Celauro, M. T. Atterrato, E. Giordano, S. Iwashita, P. Dimitri, The Bucentaur (BCNT) protein family: A long-neglected class of essential proteins required for chromatin/chromosome organization and function. *Chromosoma* **124**, 153–162 (2015).

25. G. Messina, M. T. Atterrato, Y. Prozzillo, L. Piacentini, A. Losada, P. Dimitri, The human Cranio Facial Development Protein 1 (Cfdp1) gene encodes a protein required for the maintenance of higher-order chromatin organization. *Sci. Rep.* **7**, 45022 (2017).

26. E. Luk, A. Ranjan, P. C. Fitzgerald, G. Mizuguchi, Y. Huang, D. Wei, C. Wu, Stepwise histone replacement by SWR1 requires dual activation with histone H2A.Z and canonical nucleosome. *Cell* **143**, 725–736 (2010).

27. A. Ranjan, G. Mizuguchi, P. C. FitzGerald, D. Wei, F. Wang, Y. Huang, E. Luk, C. L. Woodcock, C. Wu, Nucleosome-free region dominates histone acetylation in targeting SWR1 to promoters for H2A.Z replacement. *Cell* **154**, 1232–1245 (2013).

28. O. Willhoft, M. Ghoneim, C.-L. Lin, E. Y. D. Chua, M. Wilkinson, Y. Chaban, R. Ayala, E. A. McCormack, L. Ocloo, D. S. Rueda, D. B. Wigley, Structure and dynamics of the yeast SWR1-nucleosome complex. *Science* **362**, eaat7716 (2018).

29. R. K. Louder, G. Park, Z. Ye, J. S. Cha, A. M. Gardner, Q. Lei, A. Ranjan, E. Höllmüller, F. Stengel, B. F. Pugh, C. Wu, Molecular basis of global promoter sensing and nucleosome capture by the SWR1 chromatin remodeler. *Cell* **187**, 6849–6864.e18 (2024).

30. X. Liang, S. Shan, L. Pan, J. Zhao, A. Ranjan, F. Wang, Z. Zhang, Y. Huang, H. Feng, D. Wei, L. Huang, X. Liu, Q. Zhong, J. Lou, G. Li, C. Wu, Z. Zhou, Structural basis of H2A.Z recognition by SRCAP chromatin-remodeling subunit YL1. *Nat. Struct. Mol. Biol.* **23**, 317–323 (2016).

31. C. M. Latrick, M. Marek, K. Ouararhni, C. Papin, I. Stoll, M. Ignatyeva, A. Obri, E. Ennifar, S. Dimitrov, C. Romier, A. Hamiche, Molecular basis and specificity of H2A.Z-H2B recognition and deposition by the histone chaperone YL1. *Nat. Struct. Mol. Biol.* **23**, 309–316 (2016).

32. J. Hong, H. Feng, F. Wang, A. Ranjan, J. Chen, J. Jiang, R. Ghirlando, T. S. Xiao, C. Wu, Y. Bai, The catalytic subunit of the SWR1 remodeler is a histone chaperone for the H2A.Z-H2B dimer. *Mol. Cell* **53**, 498–505 (2014).

33. M. F. Poyton, X. A. Feng, A. Ranjan, Q. Lei, F. Wang, J. S. Zarb, R. K. Louder, G. Park, M. H. Jo, J. Ye, S. Liu, T. Ha, C. Wu, Coordinated DNA and histone dynamics drive accurate histone H2A.Z exchange. *Sci. Adv.* **8**, eabj5509 (2022).

34. X. Liu, M. Li, X. Xia, X. Li, Z. Chen, Mechanism of chromatin remodelling revealed by the Snf2-nucleosome structure. *Nature* **544**, 440–445 (2017).

35. X. Xia, X. Liu, T. Li, X. Fang, Z. Chen, Structure of chromatin remodeler Swi2/Snf2 in the resting state. *Nat. Struct. Mol. Biol.* **23**, 722–729 (2016).

36. J. P. Armache, N. Gamarra, S. L. Johnson, J. D. Leonard, S. Wu, G. J. Narlikar, Y. Cheng, Cryo-EM structures of remodeler-nucleosome intermediates suggest allosteric control through the nucleosome. *eLife* **8**, e46057 (2019).

37. L. Farnung, M. Ochmann, P. Cramer, Nucleosome-CHD4 chromatin remodeler structure maps human disease mutations. *eLife* **9**, e56178 (2020).

38. J. Yuan, K. Chen, W. Zhang, Z. Chen, Structure of human chromatin-remodelling PBAF complex bound to a nucleosome. *Nature* **605**, 166–171 (2022).

39. L. Yan, H. Wu, X. Li, N. Gao, Z. Chen, Structures of the ISWI-nucleosome complex reveal a conserved mechanism of chromatin remodeling. *Nat. Struct. Mol. Biol.* **26**, 258–266 (2019).

40. M. Li, X. Xia, Y. Tian, Q. Jia, X. Liu, Y. Lu, M. Li, X. Li, Z. Chen, Mechanism of DNA translocation underlying chromatin remodelling by Snf2. *Nature* **567**, 409–413 (2019).

41. L. Farnung, S. M. Vos, C. Wigge, P. Cramer, Nucleosome–Chd1 structure and implications for chromatin remodelling. *Nature* **550**, 539–542 (2017).

42. I. M. Nodelman, S. Das, A. M. Faustino, S. D. Fried, G. D. Bowman, J.-P. Armache, Nucleosome recognition and DNA distortion by the Chd1 remodeler in a nucleotide-free state. *Nat. Struct. Mol. Biol.* **29**, 121–129 (2022).

43. F. Kunert, F. J. Metzner, J. Jung, M. Höpfler, S. Woike, K. Schall, D. Kostrewa, M. Moldt, J.-X. Chen, S. Bantele, B. Pfander, S. Eustermann, K.-P., Hopfner, Structural mechanism of extranucleosomal DNA readout by the INO80 complex. *Sci. Adv.* **8**, eadd3189 (2022).

44. R. K. Suto, M. J. Clarkson, D. J. Tremethick, K. Luger, Crystal structure of a nucleosome core particle containing the variant histone H2A.Z. *Nat. Struct. Biol.* **7**, 1121–1124 (2000).

45. A. Obri, K. Ouararhni, C. Papin, M.-L. Diebold, K. Padmanabhan, M. Marek, I. Stoll, L. Roy, P. T. Reilly, T. W. Mak, S. Dimitrov, C. Romier, A. Hamiche, ANP32E is a histone chaperone that removes H2A.Z from chromatin. *Nature* **505**, 648–653 (2014).

46. A. S. B. Jalal, P. Girvan, E. Y. D. Chua, L. Liu, S. Wang, E. A. McCormack, M. T. Skehan, C. L. Knight, D. S. Rueda, D. B. Wigley, Stabilization of the hexasome intermediate during histone exchange by yeast SWR1 complex. *Mol. Cell* **84**, 3871–3884.e9 (2024).

47. C.-L. Lin, Y. Chaban, D. M. Rees, E. A. McCormack, L. Ocloo, D. B. Wigley, Functional characterization and architecture of recombinant yeast SWR1 histone exchange complex. *Nucleic Acids Res.* **45**, 7249–7260 (2017).

48. A. Ranjan, F. Wang, G. Mizuguchi, D. Wei, Y. Huang, C. Wu, H2A histone-fold and DNA elements in nucleosome activate SWR1-mediated H2A.Z replacement in budding yeast. *eLife* **4**, e06845 (2015).

49. R. K. Singh, J. Fan, N. Gioacchini, S. Watanabe, O. Bilsel, C. L. Peterson, Transient kinetic analysis of SWR1C-catalyzed H2A.Z deposition unravels the impact of nucleosome dynamics and the asymmetry of histone exchange. *Cell Rep.* **27**, 374–386.e4 (2019).

50. F. Liu, A. Putnam, E. Jankowsky, ATP hydrolysis is required for DEAD-box protein recycling but not for duplex unwinding. *Proc. Natl. Acad. Sci. U.S.A.* **105**, 20209–20214 (2008).

51. F. Liu, A. A. Putnam, E. Jankowsky, DEAD-box helicases form nucleotide-dependent, long-lived complexes with RNA. *Biochemistry* **53**, 423–433 (2014).

52. R. Ren, S. Ghassabi Kondalaji, G. D. Bowman, The Chd1 chromatin remodeler forms long-lived complexes with nucleosomes in the presence of ADP·BeF3- and transition state analogs. *J. Biol. Chem.* **294**, 18181–18191 (2019).

53. J. M. Kim, P. Visanpattanasin, V. Jou, S. Liu, X. Tang, Q. Zheng, K. Y. Li, J. Snedeker, L. D. Lavis, T. Lionnet, C. Wu, Single-molecule imaging of chromatin remodelers reveals role of ATPase in promoting fast kinetics of target search and dissociation from chromatin. *eLife* **10**, e69387 (2021).

54. B. C. Tilly, G. E. Chalkley, J. A. van der Knaap, Y. M. Moshkin, T. W. Kan, D. H. Dekkers, J. A. Demmers, C. P. Verrijzer, In vivo analysis reveals that ATP-hydrolysis couples remodeling to SWI/SNF release from chromatin. *eLife* **10**, e69424 (2021).

55. S. Sainsbury, C. Bernecky, P. Cramer, Structural basis of transcription initiation by RNA polymerase II. *Nat. Rev. Mol. Cell Biol.* **16**, 129–143 (2015).

56. R. K. Louder, Y. He, J. R. López-Blanco, J. Fang, P. Chacón, E. Nogales, Structure of promoter-bound TFIID and model of human pre-initiation complex assembly. *Nature* **531**, 604–609 (2016).

57. E. Nogales, R. K. Louder, Y. He, Structural insights into the eukaryotic transcription initiation machinery. *Annu. Rev. Biophys.* **46**, 59–83 (2017).

58. S. Buratowski, S. Hahn, L. Guarente, P. A. Sharp, Five intermediate complexes in transcription initiation by RNA polymerase II. *Cell* **56**, 549–561 (1989).

59. S. P. Solow, L. Lezina, P. M. Lieberman, Phosphorylation of TFIIA stimulates TATA binding protein-TATA interaction and contributes to maximal transcription and viability in yeast. *Mol. Cell. Biol.* **19**, 2846–2852 (1999).

60. T. Høiby, H. Zhou, D. J. Mitsiou, H. G. Stunnenberg, A facelift for the general transcription factor TFIIA. *Biochim. Biophys. Acta* **1769**, 429–436 (2007).

61. C. Schrenk, V. Fetz, C. Vallet, C. Heiselmayer, E. Schröder, A. Hensel, A. Hahlbrock, D. Wünsch, D. Goesswein, C. Bier, N. Habtemichael, G. Schneider, R. H. Stauber, S. K. Knauer, TFIIA transcriptional activity is controlled by a "cleave-and-run" Exportin-1/Taspase 1-switch. *J. Mol. Cell Biol.* **10**, 33–47 (2018).

62. S. Iwashita, T. Suzuki, T. Yasuda, K. Nakashima, T. Sakamoto, T. Kohno, I. Takahashi, T. Kobayashi, Y. Ohno-Iwashita, S. Imajoh-Ohmi, S.-Y. Song, N. Dohmae, Mammalian Bcnt/Cfdp1, a potential epigenetic factor characterized by an acidic stretch in the disordered N-terminal and Ser250 phosphorylation in the conserved C-terminal regions. *Biosci. Rep.* **35**, e00228 (2015).

63. P. V. Hornbeck, B. Zhang, B. Murray, J. M. Kornhauser, V. Latham, E. Skrzypek, PhosphoSitePlus, 2014: Mutations, PTMs and recalibrations. *Nucleic Acids Res.* **43**, D512–D520 (2015).

64. S. M. Abmayr, T. Yao, T. Parmely, J. L. Workman, Preparation of nuclear and cytoplasmic extracts from mammalian cells. *Curr. Protoc. Mol. Biol.* doi.org/10.1002/0471142727.mb1201s75 (2006).

65. P. N. Dyer, R. S. Edayathumangalam, C. L. White, Y. Bao, S. Chakravarthy, U. M. Muthurajan, K. Luger, Reconstitution of nucleosome core particles from recombinant histones and DNA. *Methods Enzymol.* **375**, 23–44 (2004).

66. T. Juven-Gershon, S. Cheng, J. T. Kadonaga, Rational design of a super core promoter that enhances gene expression. *Nat. Methods* **3**, 917–922 (2006).

67. P. T. Lowary, J. Widom, New DNA sequence rules for high affinity binding to histone octamer and sequence-directed nucleosome positioning. *J. Mol. Biol.* **276**, 19–42 (1998).

68. B. Yariv, E. Yariv, A. Kessel, G. Masrati, A. B. Chorin, E. Martz, I. Mayrose, T. Pupko, N. Ben-Tal, Using evolutionary data to make sense of macromolecules with a "face-lifted" ConSurf. *Protein Sci.* **32**, e4582 (2023).

69. H. Ashkenazy, S. Abadi, E. Martz, O. Chay, I. Mayrose, T. Pupko, N. Ben-Tal, ConSurf 2016: An improved methodology to estimate and visualize evolutionary conservation in macromolecules. *Nucleic Acids Res.* **44**, W344–W350 (2016).

70. M. Landau, I. Mayrose, Y. Rosenberg, F. Glaser, E. Martz, T. Pupko, N. Ben-Tal, ConSurf 2005: The projection of evolutionary conservation scores of residues on protein structures. *Nucleic Acids Res.* **33**, W299–W302 (2005).

71. J. Jumper, R. Evans, A. Pritzel, T. Green, M. Figurnov, O. Ronneberger, K. Tunyasuvunakool, R. Bates, A. Žídek, A. Potapenko, A. Bridgland, C. Meyer, S. A. A. Kohl, A. J. Ballard, A. Cowie, B. Romera-Paredes, S. Nikolov, R. Jain, J. Adler, T. Back, S. Petersen, D. Reiman, E. Clancy, M. Zielinski, M. Steinegger, M. Pacholska, T. Berghammer, S. Bodenstein, D. Silver, O. Vinyals, A. W. Senior, K. Kavukcuoglu, P. Kohli, D. Hassabis, Highly accurate protein structure prediction with AlphaFold. *Nature* **596**, 583–589 (2021).

72. M. Mirdita, K. Schütze, Y. Moriwaki, L. Heo, S. Ovchinnikov, M. Steinegger, ColabFold: Making protein folding accessible to all. *Nat. Methods* **19**, 679–682 (2022).

73. R. Evans, M. O'Neill, A. Pritzel, N. Antropova, A. Senior, T. Green, A. Žídek, R. Bates, S. Blackwell, J. Yim, O. Ronneberger, S. Bodenstein, M. Zielinski, A. Bridgland, A. Potapenko,

A. Cowie, K. Tunyasuvunakool, R. Jain, E. Clancy, P. Kohli, J. Jumper, D. Hassabis, Protein complex prediction with AlphaFold-Multimer. bioRxiv 463034 [Preprint] (2021); https://doi.org/10.1101/2021.10.04.463034.

74. F. Madeira, N. Madhusoodanan, J. Lee, A. Eusebi, A. Niewielska, A. R. N. Tivey, R. Lopez, S. Butcher, The EMBL-EBI Job Dispatcher sequence analysis tools framework in 2024. *Nucleic Acids Res.* **52**, W521–W525 (2024).

75. D. N. Mastronarde, Automated electron microscope tomography using robust prediction of specimen movements. *J. Struct. Biol.* **152**, 36–51 (2005).

76. D. Kimanius, L. Dong, G. Sharov, T. Nakane, S. H. W. Scheres, New tools for automated cryo-EM single-particle analysis in RELION-4.0. *Biochem. J.* **478**, 4169–4185 (2021).

77. S. Q. Zheng, E. Palovcak, J.-P. Armache, K. A. Verba, Y. Cheng, D. A. Agard, MotionCor2: Anisotropic correction of beam-induced motion for improved cryo-electron microscopy. *Nat. Methods* **14**, 331–332 (2017).

78. K. Zhang, Gctf: Real-time CTF determination and correction. *J. Struct. Biol.* **193**, 1–12 (2016).

79. R. Henderson, A. Sali, M. L. Baker, B. Carragher, B. Devkota, K. H. Downing, E. H. Egelman, Z. Feng, J. Frank, N. Grigorieff, W. Jiang, S. J. Ludtke, O. Medalia, P. A. Penczek, P. B. Rosenthal, M. G. Rossmann, M. F. Schmid, G. F. Schröder, A. C. Steven, D. L. Stokes, J. D. Westbrook, W. Wriggers, H. Yang, J. Young, H. M. Berman, W. Chiu, G. J. Kleywegt, C. L. Lawson, Outcome of the first electron microscopy validation task force meeting. *Structure* **20**, 205–214 (2012).

80. A. Punjani, J. L. Rubinstein, D. J. Fleet, M. A. Brubaker, cryoSPARC: Algorithms for rapid unsupervised cryo-EM structure determination. *Nat. Methods* **14**, 290–296 (2017).

81. A. Rohou, N. Grigorieff, CTFFIND4: Fast and accurate defocus estimation from electron micrographs. *J. Struct. Biol.* **192**, 216–221 (2015).

82. R. Sanchez-Garcia, J. Gomez-Blanco, A. Cuervo, J. M. Carazo, C. O. S. Sorzano, J. Vargas, DeepEMhancer: A deep learning solution for cryo-EM volume post-processing. *Commun. Biol.* **4**, 874 (2021).

83. P. Emsley, B. Lohkamp, W. G. Scott, K. Cowtan, Features and development of Coot. *Acta Crystallogr. D Biol. Crystallogr.* **66**, 486–501 (2010).

84. T. I. Croll, ISOLDE: A physically realistic environment for model building into low-resolution electron-density maps. *Acta Crystallogr. D Struct. Biol.* **74**, 519–530 (2018).

85. E. C. Meng, T. D. Goddard, E. F. Pettersen, G. S. Couch, Z. J. Pearson, J. H. Morris, T. E. Ferrin, UCSF ChimeraX: Tools for structure building and analysis. *Protein Sci.* **32**, e4792 (2023).

86. D. Liebschner, P. V. Afonine, M. L. Baker, G. Bunkóczi, V. B. Chen, T. I. Croll, B. Hintze, L. W. Hung, S. Jain, A. J. McCoy, N. W. Moriarty, R. D. Oeffner, B. K. Poon, M. G. Prisant, R. J. Read, J. S. Richardson, D. C. Richardson, M. D. Sammito, O. V. Sobolev, D. H. Stockwell, T. C. Terwilliger, A. G. Urzhumtsev, L. L. Videau, C. J. Williams, P. D. Adams, Macromolecular structure determination using X-rays, neutrons and electrons: Recent developments in Phenix. *Acta Crystallogr. D Struct. Biol.* **75**, 861–877 (2019).

87. P. V. Afonine, B. P. Klaholz, N. W. Moriarty, B. K. Poon, O. V. Sobolev, T. C. Terwilliger, P. D. Adams, A. Urzhumtsev, New tools for the analysis and validation of cryo-EM maps and atomic models. *Acta Crystallogr. D Struct. Biol.* **74**, 814–840 (2018).

88. C. J. Williams, J. J. Headd, N. W. Moriarty, M. G. Prisant, L. L. Videau, L. N. Deis, V. Verma, D. A. Keedy, B. J. Hintze, V. B. Chen, S. Jain, S. M. Lewis, W. B. Arendall III, J. Snoeyink, P. D. Adams, S. C. Lovell, J. S. Richardson, D. C. Richardson, MolProbity: More and better reference data for improved all-atom structure validation. *Protein Sci.* **27**, 293–315 (2018).

89. J. Abramson, J. Adler, J. Dunger, R. Evans, T. Green, A. Pritzel, O. Ronneberger, L. Willmore, A. J. Ballard, J. Bambrick, S. W. Bodenstein, D. A. Evans, C.-C. Hung, M. O'Neill, D. Reiman, K. Tunyasuvunakool, Z. Wu, A. Žemgulytė, E. Arvaniti, C. Beattie, O.

Bertolli, A. Bridgland, A. Cherepanov, M. Congreve, A. I. Cowen-Rivers, A. Cowie, M. Figurnov, F. B. Fuchs, H. Gladman, R. Jain, Y. A. Khan, C. M. R. Low, K. Perlin, A. Potapenko, P. Savy, S. Singh, A. Stecula, A. Thillaisundaram, C. Tong, S. Yakneen, E. D. Zhong, M. Zielinski, A. Žídek, V. Bapst, P. Kohli, M. Jaderberg, D. Hassabis, J. M. Jumper, Accurate structure prediction of biomolecular interactions with AlphaFold 3. *Nature* **630**, 493–500 (2024).

90. T. D. Goddard, C. C. Huang, E. C. Meng, E. F. Pettersen, G. S. Couch, J. H. Morris, T. E. Ferrin, UCSF ChimeraX: Meeting modern challenges in visualization and analysis. *Protein Sci.* **27**, 14–25 (2018).

91. C.-C. Hsu, J. Shi, C. Yuan, D. Zhao, S. Jiang, J. Lyu, X. Wang, H. Li, H. Wen, W. Li, X. Shi, Recognition of histone acetylation by the GAS41 YEATS domain promotes H2A.Z deposition in non-small cell lung cancer. *Genes Dev.* **32**, 58–69 (2018).

92. H. J. Cho, H. Li, B. M. Linhares, E. Kim, J. Ndoj, H. Miao, J. Grembecka, T. Cierpicki, GAS41 Recognizes Diacetylated Histone H3 through a Bivalent Binding Mode. *ACS Chem. Biol.* **13**, 2739–2746 (2018).

93. M. Kikuchi, S. Morita, M. Goto, M. Wakamori, K. Katsura, K. Hanada, M. Shirouzu, T. Umehara, Elucidation of binding preferences of YEATS domains to site-specific acetylated nucleosome core particles. *J. Biol. Chem.* **298**, 102164 (2022).

94. M. Kikuchi, S. Takase, T. Konuma, K. Noritsugu, S. Sekine, T. Ikegami, A. Ito, T. Umehara, GAS41 promotes H2A.Z deposition through recognition of the N terminus of histone H3 by the YEATS domain. *Proc. Natl. Acad. Sci. U.S.A.* **120**, e2304103120 (2023).

95. M.-E. Lalonde, X. Cheng, J. Côté, Histone target selection within chromatin: An exemplary case of teamwork. *Genes Dev.* **28**, 1029–1041 (2014).

96. M. R. Marunde, H. A. Fuchs, J. M. Burg, I. K. Popova, A. Vaidya, N. W. Hall, E. N. Weinzapfel, M. J. Meiners, R. Watson, Z. B. Gillespie, H. F. Taylor, L. Mukhsinova, U. C. Onuoha, S. A. Howard, K. Novitzky, E. T. McAnarney, K. Krajewski, M. W. Cowles, M. A.

Cheek, Z.-W. Sun, B. J. Venters, M.-C. Keogh, C. A. Musselman, Nucleosome conformation dictates the histone code. *eLife* **13**, e78866 (2024).

97. A. Patil, A. R. Strom, J. A. Paulo, C. K. Collings, K. M. Ruff, M. K. Shinn, A. Sankar, K. S. Cervantes, T. Wauer, J. D. St Laurent, G. Xu, L. A. Becker, S. P. Gygi, R. V. Pappu, C. P. Brangwynne, C. Kadoch, A disordered region controls cBAF activity via condensation and partner recruitment. *Cell* **186**, 4936–4955.e26 (2023).

98. R. S. Greenberg, H. K. Long, T. Swigut, J. Wysocka, Single amino acid change underlies distinct roles of H2A.Z subtypes in human syndrome. *Cell* **178**, 1421–1436.e24 (2019).

99. G. A. Armeev, A. S. Kniazeva, G. A. Komarova, M. P. Kirpichnikov, A. K. Shaytan, Histone dynamics mediate DNA unwrapping and sliding in nucleosomes. *Nat. Commun.* **12**, 2387 (2021).
